# Metabolic labelling of cholesteryl glucosides in *Helicobacter pylori* reveals how the uptake of human lipids enhances bacterial virulence†
†Electronic supplementary information (ESI) available: General reagents and instruments of chemical synthesis, supplementary schemes, synthetic procedures, supplementary spectra data of selected compounds, supplementary methods, supplementary figures and supplementary tables. See DOI: 10.1039/c6sc00889e


**DOI:** 10.1039/c6sc00889e

**Published:** 2016-06-03

**Authors:** Hau-Ming Jan, Yi-Chi Chen, Yu-Yin Shih, Yu-Chen Huang, Zhijay Tu, Arun B. Ingle, Sheng-Wen Liu, Ming-Shiang Wu, Jacquelyn Gervay-Hague, Kwok-Kong Tony Mong, Yet-Ran Chen, Chun-Hung Lin

**Affiliations:** a Institute of Biological Chemistry , Academia Sinica , No. 128 Academia Road Section 2, Nan-Kang , Taipei , 11529 , Taiwan . Email: chunhung@gate.sinica.edu.tw; b Institute of Biochemical Sciences and Department of Chemistry , National Taiwan University , Taipei , 10617 , Taiwan; c Agriculture Biotechnology Research Center , Academia Sinica , Taipei , 11529 , Taiwan; d Division of Gastroenterology , Department of Internal Medicine , National Taiwan University Hospital , Taipei , 10002 , Taiwan; e Department of Chemistry , University of California , Davis , CA 95618 , USA; f Department of Applied Chemistry , National Chiao-Tung University , Hsin-Chu 300 , Taiwan

## Abstract

*Helicobacter pylori* infects approximately half of the human population and is the main cause of various gastric diseases. This pathogen is auxotrophic for cholesterol, which it converts upon uptake to various cholesteryl α-glucoside derivatives, including cholesteryl 6′-acyl and 6′-phosphatidyl α-glucosides (CAGs and CPGs). Owing to a lack of sensitive analytical methods, it is not known if CAGs and CPGs play distinct physiological roles or how the acyl chain component affects function. Herein we established a metabolite-labelling method for characterising these derivatives qualitatively and quantitatively with a femtomolar detection limit. The development generated an MS/MS database of CGds, allowing for profiling of all the cholesterol-derived metabolites. The subsequent analysis led to the unprecedented information that these bacteria acquire phospholipids from the membrane of epithelial cells for CAG biosynthesis. The resulting increase in longer or/and unsaturated CAG acyl chains helps to promote lipid raft formation and thus delivery of the virulence factor CagA into the host cell, supporting the idea that the host/pathogen interplay enhances bacterial virulence. These findings demonstrate an important connection between the chain length of CAGs and the bacterial pathogenicity.

## Abstract 2

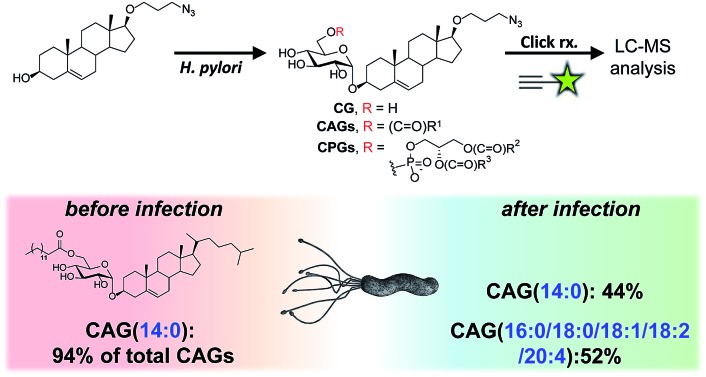
 *Helicobacter pylori* infects approximately half of the human population and is the main cause of various gastric diseases.

## Introduction

Infecting ∼50% of the world population, *Helicobacter pylori* causes chronic gastritis, which is asymptomatic in the majority of carriers but is considered a major risk factor for the development of gastric and duodenal ulcers and gastric malignancies, including mucosa-associated lymphoid tissue lymphoma and gastric adenocarcinoma.^1^ This bacterium has an exceptional ability to persist and establish chronic infection, unlike other Gram-negative bacterial pathogens, and represents a highly successful pathogen because it is capable of evading, subverting and manipulating the host's immune system.^2^,^3^ For example, cytotoxin-associated gene A (CagA)^4^ is an *H. pylori* virulence factor that enters host cells *via* type IV secretion system (T4SS)-mediated translocation.^5^ CagA then undergoes tyrosine phosphorylation by c-Src family kinases,^6^,^7^ and the phosphorylated CagA then activates several cellular signalling processes, including those leading to cancer development.^8^,^9^ Interestingly, the translocation process is not restricted to just the transfer from *H. pylori* to the host. The pathogen also receives molecules such as l-fucose^10^ and cholesterol^3^,^11^,^12^ from the host for various purposes.

*H. pylori* assimilates cholesterol into its membrane by taking up cholesterol from the medium or from epithelial cells of the stomach.^13^ Upon uptake, the bacterial cells modify the cholesterol by α-glucosylation. Specifically, the glucosyltransferase encoded by *hp0421* catalyses the transfer of glucose to the 3β-hydroxyl group of cholesterol, yielding cholesteryl α-d-glucopyranoside (CG). An acyl or phosphatidyl group is then attached to the O6′ of glucose in CG to produce cholesteryl-6′-*O*-acyl-α-d-glucopyranoside (CAG) or cholesteryl-6′-*O*-phosphatidyl-α-d-glucopyranoside (CPG), respectively.^14^,^15^ These cholesteryl glucoside derivatives (CGds) have been shown to be essential for several physiological activities, such as antibiotic resistance,^12^ CagA translocation,^16^ the prevention of phagocytosis by macrophages^3^ and the activation of invariant natural killer T cells.^17^,^18^ CAG and CPG are further diversified by the addition of different fatty acid chains to the acyl and phosphatidyl groups, respectively. Owing to limitations in the purification and analysis, typically these CGds are examined collectively instead of being separated and compared to determine whether the acyl or phosphatidyl chain plays a role in an activity of interest.

Herein, we developed a specific metabolite-tagging method to facilitate the purification and characterisation of *H. pylori* CGds. Based on ultrahigh-performance liquid chromatography mass spectrometry (UPLC-MS), this method not only allowed the structural determination and quantitative characterisation of all CGds, but also offered a great advantage in detection sensitivity and the isolation of various CGds that are difficult to obtain synthetically or by other means. Most importantly, the resulting analysis demonstrated the incorporation of the host phospholipids led to a significant increase in CAGs containing longer or/and unsaturated fatty acid chains. This change was further found to promote the translocation and subsequent phosphorylation of CagA through the enhanced formation of lipid rafts, demonstrating an interactive strategy to enhance bacterial virulence.

## Results

### Synthesis and evaluation of the tagged metabolites

**Fig. 1:**
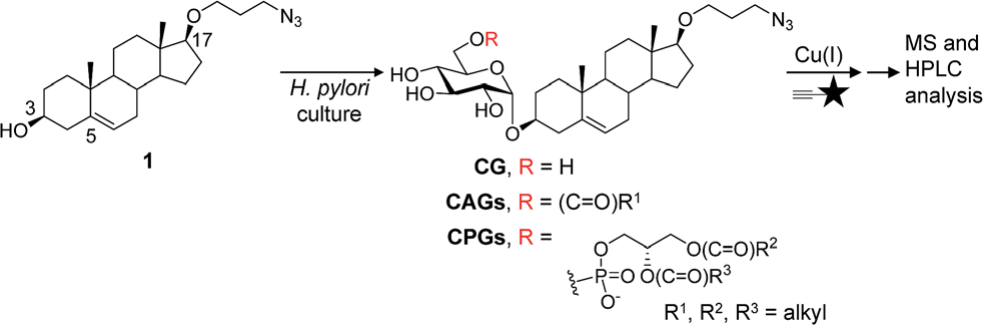
Schematic of the metabolic labelling of *H. pylori* CGds. 17β-([3′′-Azidopropoxy)-5-androsten-3β-ol (**1**; an azide-containing analogue of cholesterol) was added to *H. pylori* culture and incorporated into the biosynthetic pathway to produce various CGd analogues. After harvest and Folch extraction, these analogues were further reacted with a fluorescent alkyne (
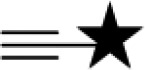
) *via* Cu(i)-catalysed 1,3-dipolar cycloaddition to obtain the conjugated products, which were then analysed by HPLC and MS.

Our metabolic labelling approach utilises azide-containing cholesterol analogues in bacterial cell culture (Fig. 1). After the cells were harvested, lipids were extracted and subjected to click chemistry (1,3-dipolar cycloaddition) with a fluorescently-tagged alkyne. We first examined the cholesterol analogues that are comparable with cholesterol in terms of biosynthetic incorporation *in vivo*. We synthesised three azide-containing cholesterol analogues, namely 17β-([3′′-azidopropoxy)-5-androsten-3β-ol (compound **1**), 17β-([6′′-azidohexanoxy)-5-androsten-3β-ol (**2**) and 17β-([9′′-azidononanoxyl)-5-androsten-3β-ol (**3**), as shown in the ESI Scheme 1.† These analogues all have the same four fused rings as cholesterol, but differ in the azide-containing side chain introduced at O17β. To evaluate if the three analogues were suitable for incorporation into the biosynthetic pathway, the cholesteryl α-glucosyltransferase gene *hp0421* was cloned and the protein was then overexpressed and purified as previously reported.^15^ Steady-state kinetics analysis (Table S1†)^19^,^20^ demonstrated that the catalytic efficiency (*k*_cat_/*K*_m_) values of compounds **1–3** are slightly smaller than that of cholesterol, being in the range of 84–59% that of cholesterol. **1** appeared to be the best among the three and was thus selected for the further investigations. Moreover, four other cholesterol analogues (the structures of which are shown in Table S1†) displayed much lower affinity for the enzyme, including ergosterol (13% that of cholesterol),^15^ stigmasterol (7%),^15^ 22-NBD-cholesterol (not detectable) and 25-NBD-cholesterol (not detectable); with the last two compounds representing fluorescent analogues widely used for imaging studies.^21^–^23^ The side chain at C17 appeared to be critical to the enzymatic glucosylation.

Compound **1** was evaluated by thin-layer chromatography and phospholipid composition analyses^24^ to confirm that it was equivalent to cholesterol in the biosynthesis of CGds (Fig. S1†). The latter examined the levels of several phospholipids in addition to CPG. The results indicated that **1** and cholesterol yielded similar percentages of the major phospholipids in *H. pylori*, including phosphatidylethanolamine (PE), cardiolipin and phosphatidylglycerol.

We considered two factors when preparing suitable alkyne-containing fluorescent compounds for the click chemistry, namely reactivity (potential yield) and stability. Two fluorophore-possessing alkynes were designed and synthesised, such as 4-*N*-methylamino-1,8-napthalimidopropyne (MAN) and 4-*N*,*N*-dimethylamino-1,8-naphthalimidopropyne (DAN)^25^ (ESI Scheme 2†). Both MAN and DAN, as well as several commercially available fluorescent dyes (Fig. S2B†), were examined by Cu[I]-catalysed click chemistry. The reaction yield and stability of MAN were higher than those of DAN and the other fluorescent dyes (Fig. S2†).

### Structural determination and quantification of the CGds

**Fig. 2:**
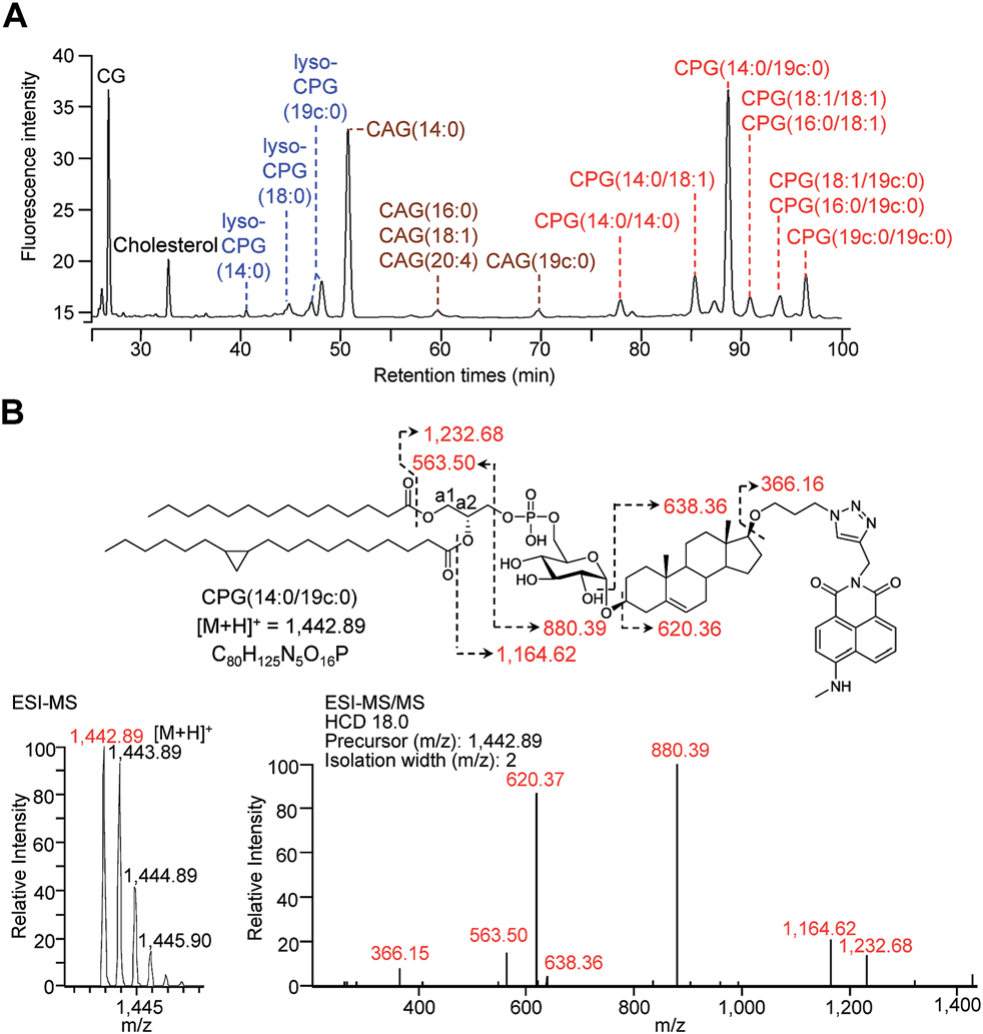
HPLC separation and identification of various fluorophore-labelled CGds. (A) HPLC chromatogram of various labelled CGds, including cholesterol, CG, CAGs, CPGs and lyso-CPGs. Compounds were detected by fluorescence (*λ*_ex_ = 428, *λ*_em_ = 528 nm). (B) Molecular structure and MS analysis of CPG(14 : 0/19c : 0). The parent ion was identified by ESI-MS and the fragmented ions by ESI-MS/MS. The corresponding structures and molecular weights are indicated by dashed arrows and numbers.

For the metabolic labelling of CGds, *H. pylori* was treated with **1** and grown under microaerobic conditions at 37 °C. After 4 days, the bacterial cells were collected and subjected to Folch extraction.^26^ The extracts were reacted with MAN in the presence of tris(benzyltriazolylmethyl)amine, sodium ascorbate and CuSO_4_ for 1 h. The resulting compounds were separated by high-performance liquid chromatography and detected by fluorescence (HPLC-fluorescence; C18-reversed-phase column; *λ*_ex_ = 428 nm, *λ*_em_ = 528 nm) (Fig. 2A). The CGds were also identified by UPLC-MS in the product ion scan mode (Fig. 2B, S3 and Table S2†).^27^ HPLC-fluorescence analysis revealed fluorophore-conjugated analogues of cholesterol and CG plus three major groups of compounds, namely CAGs, CPGs and lyso-CPGs (these fluorophore-conjugated analogues of CAGs, CPGs and lyso-CPGs derived from **1** are hereafter referred to as CAGs, CPGs and lyso-CPGs, respectively, unless otherwise specified). These compounds appeared in three groups in the HPLC chromatogram due to differences in polarity. The structure of each compound was identified by MS and confirmed by tandem MS (MS/MS). The errors for the precursor *m*/*z* and for relative isotope abundance obtained by UPLC-MS were <5 ppm and 5%, respectively (Table S2†). For instance, CPG(14 : 0/19c : 0) (see Fig. 2B for the molecular structure) consists of two esterified fatty acids : myristic acid (14 : 0) and another (19c : 0) that contains a cyclopropane at C9 of the acyl chain.^28^,^29^ In the MS/MS analysis of CPG(14 : 0/19c : 0), the predicted product ions for the functional groups of the fatty acids, phosphate, glucose, steroid, triazole and 4-*N*-methylamino-1,8-napthalimide (the fluorophore) were observed (Fig. 2B). The MS and corresponding MS/MS data for the other CGds are provided in Fig. S3.† We also performed ^1^H, ^13^C or/and ^31^P NMR analysis of CPG(14 : 0/19c : 0) and CAG(14 : 0) (ESI† spectra data 1, 2 and 3) to confirm that the phosphatidyl group is indeed attached to the O6 of glucose and that C1 of the glucose forms an α-linkage with O3 of the steroid.^30^ The phospholipases A1 and A2 (PLA1 and PLA2) were applied in the UPLC-MS analysis^31^,^32^ to assign where the two fatty acid chains of CPG(14 : 0/19c : 0) are attached to the phosphatidyl glycerol. The fatty acids (14 : 0) and (19c : 0) were found to be specifically linked to the a1- and a2-positions, respectively (Fig. S4A–C†). It was noted that acyl migration occurred after cleavage of CPG(14 : 0/19c : 0) at a1 using PLA1, *i.e.* the majority of the remaining ester at the a2 position moved to the a1 position *via* an intramolecular transfer (Fig. S4D–F†).^33^ Additionally, lyso-CPGs were found to exist as a mixture of regioisomers, with the acyl chain attached to either the a1 or a2 position (Fig. S4G†). These were designated as 2-lyso-CPG(19c : 0) or 1-lyso-CPG(19c : 0), respectively, indicating that the ester at a2 or a1 is hydrolyzed.

**Fig. 3:**
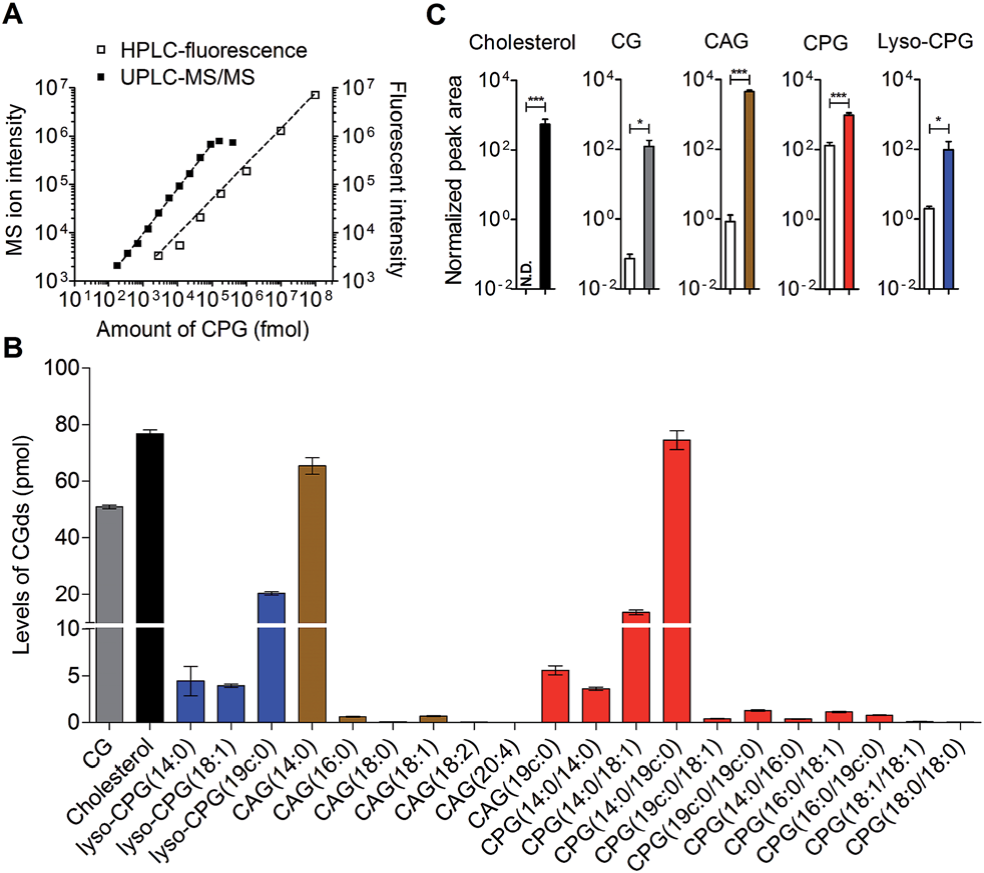
Quantitative analysis of the labelled CGds. (A) Calibration curves of CPG(14 : 0/19c : 0) based on HPLC-fluorescence (*λ*_ex_ = 428, *λ*_em_ = 528 nm) and UPLC-MS/MS operated in the product ion scan mode with a specific fragment selected for extracted ion chromatography quantification. The dynamic ranges of UPLC-MS/MS and HPLC-fluorescence were 0.088–100 pmol and 2.8 pmol–100 nmol per 10^6^ bacteria, respectively. (B) Levels of various CGds per 10^6^ bacteria as analysed by UPLC-MS/MS. (C) Enhanced ionization efficiency of fluorophore-labelled CGds in the mass spectrometry. *H. pylori* cells were pretreated with cholesterol (white bar) or compound **1** (grey bar) for two days. After harvest, the cholesterol-fed cells were extracted and directly analysed by UPLC-MS, whereas the **1**-pretreated cells were extracted, labelled with MAN by click reaction and then analysed by UPLC-MS. The normalised peak area was determined by the peak area of CGds divided by the peak area of the internal standard (phosphoinositol). N.D., not detected, denotes a ratio of signal to noise of below three. Data shown were from three biological replicates. Error bars represent the standard deviations. All the statistically significant differences are indicated with asterisks; *P < 0.05, **P < 0.01, or ***P < 0.001 based on student's *t* test.

HPLC-fluorescence and UPLC-MS/MS were employed to quantify the levels of CGds in *H. pylori*. The former was based on the integration of signals in the LC chromatogram, whereas the latter relied on the peak area of selected specific fragments for the extracted ion chromatograms from using the Orbitrap UPLC-MS system operated in the product ion scan mode. Table S3† shows the *m*/*z* of the designated target and fragmented ions as well as the collision modes/energies. The chromatograms (Fig. S5†) show the selected ion chromatograms of all the CGds at *m*/*z* 366.16 or 620.36, which represent the *m*/*z* of the fragmented ions (daughter ions) shown in Table S3.† To quantify the CGds and determine the sensitivity of detection, CPG(14 : 0/19c : 0) was purified to >99% purity (determined by HPLC-fluorescence and UPLC-MS) and used to establish calibration curves (Fig. 3A) in a linear range between 2.8 pmol and 100 nmol per 10^6^ bacteria for HPLC-fluorescence analysis, and between 0.088 and 100 pmol for UPLC-MS/MS. The calibration curves of CG, CAG(14 : 0), CPG(14 : 0/19c : 0) and lyso-CPG(19c : 0) using UPLC-MS/MS and HPLC-fluorescence are also shown in Fig. S6.† In comparison with HPLC-fluorescence, UPLC-MS/MS was found to be more suitable for measuring the CGds in very low abundance (<5 pmol per 10^6^ bacteria), such as CAG(18 : 0), CAG(18 : 2), CPG(14 : 0/16 : 0) and CPG(18 : 0/18 : 0) (Fig. 3B). The detection sensitivity in UPLC-MS/MS for the fluorophore-conjugated derivatives (0.088 pmol) was significantly enhanced relative to the unconjugated analogues, which is attributable to the dominant protonation of the incorporated fluorophore. In particular, whereas the native CG and CAGs have no charge, the fluorophore-conjugated analogues had 100- and 10 000-fold enhanced intensities, respectively (Fig. 3C).

### Uptake of human phospholipids for CAG biosynthesis in *H. pylori*

**Fig. 4:**
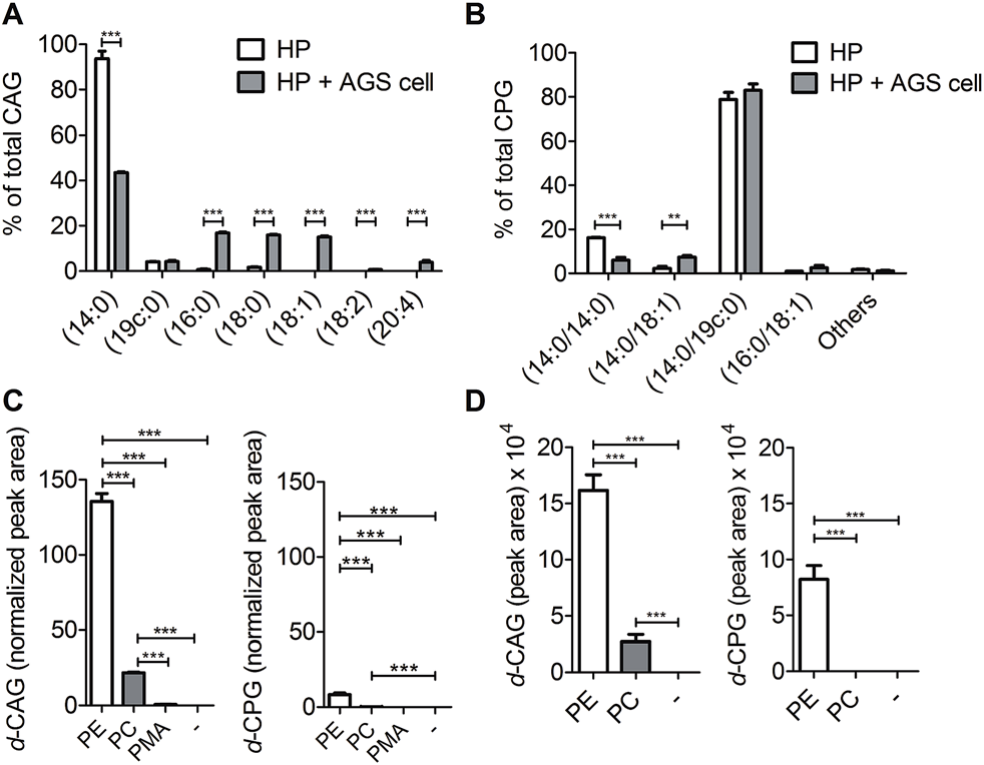
Utilisation of human lipids for CAG and CPG biosynthesis in *H. pylori*. Relative levels of (A) CAGs and (B) CPGs containing various acyl chains. *H. pylori* (HP; standard type, 26695) was incubated with or without human AGS cells for 6 h, and then CAGs and CPGs were extracted from the cultures. “Others” includes the minor CPGs, including CPG(18 : 0/19c : 0), CPG(19c : 0/19c : 0), CPG(18 : 0/18 : 0), CPG(16 : 0/18 : 1) and CPG(18 : 1/18 : 1). (C) Relative levels of deuterium-labelled CAG and CPG in the culture of *H. pylori*. *H. pylori* was treated with deuterium-labelled phosphatidylethanolamine (PE) or phosphatidylcholine (PC) or palmitic acid (PMA) for 7 h and then subjected to extensive washes to remove the unabsorbed compound. Both PE and PC contained deuterium-labelled palmitic acid (16 : 0) and unlabelled oleic acid (18 : 1). PMA contained deuterium-labelled palmitic acid (16 : 0). The levels of deuterium-labelled CAG and CPG (namely *d*-CAG and *d*-CPG, respectively), were quantified by UPLC-MS/MS and calculated based on the abundance of spiked internal standard (phosphoinositol). (D) Incorporation of deuterium-labelled fatty acids from phospholipids into CAGs and CPGs. AGS cells were treated with deuterium-labelled PE or PC for 1 h and then infected with *H. pylori* (26695) for 6 h. PE and PC contained deuterium-labelled palmitic acid (16 : 0) and unlabelled oleic acid (18 : 1). The levels of deuterium-labelled CAGs and CPGs (*d*-CAG and *d*-CPG, respectively) were determined by the area of the product ion signals in the UPLC-MS spectra. Data shown are from three biological replicates. Error bars represent the standard deviations. All statistically significant differences are indicated with asterisks; **P < 0.01 or ***P < 0.001 based on Student's *t* test.

We next applied these labelling and analysis methods to compare the lipid composition of CAGs and CPGs between *H. pylori* cultured alone and co-cultures of *H. pylori* with AGS cells (a human gastric cancer cell line; *i.e. H. pylori*-infected AGS cells). Two major CAGs were observed in the bacterial cells cultured alone (Fig. 4A), namely CAG(14 : 0) (93.7%) and CAG(19c : 0) (4.0%), in agreement with the fact that these two fatty acids are dominant in *H. pylori*.^14^ In contrast, the co-cultured *H. pylori* generated significant amounts of CAGs containing longer or/and unsaturated acyl chains, such as CAG(16 : 0), CAG(18 : 0), CAG(18 : 1), CAG(18 : 2) and CAG(20 : 4) (Fig. 4A and S7A†). These lipid chains rarely exist in *H. pylori* cultures, suggesting that they may have originated from the human cells. Contrary to prediction, there was no obvious difference in the lipid compositions or percentages of CPGs between the bacterial culture and the co-culture (Fig. 4B and S7B†).

**Fig. 5:**
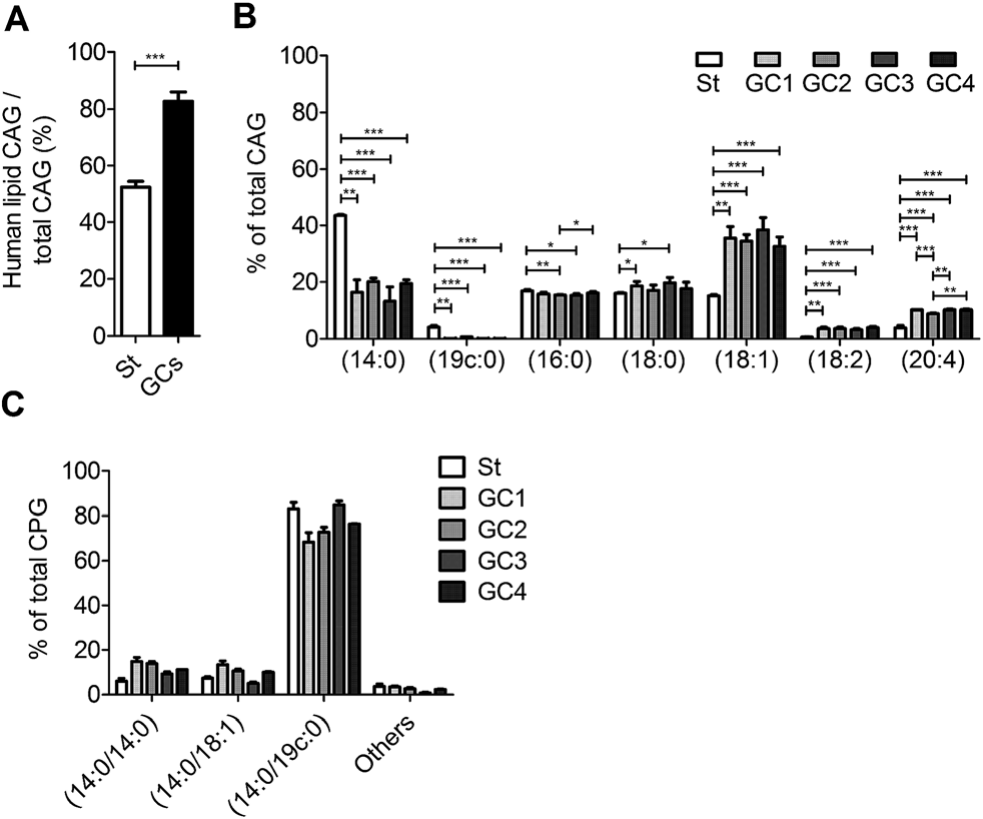
Utilisation of human lipids for CAG biosynthesis in standard type *H. pylori* and in clinical strains from patients with gastric cancer. Standard type *H. pylori* (St; 26695) and clinical isolates (GC1–GC4) were cultured with AGS cells, and the amounts of CAGs containing different fatty acid chains were measured by UPLC-MS. (A) Ratio of human-lipid-containing CAGs to total CAGs. (B) Relative levels of CAGs containing different acyl chains. Human-lipid-containing CAGs are composed of CAG(16 : 0), CAG(18 : 0), CAG(18 : 1), CAG(18 : 2) and CAG(20 : 4). Total CAGs also include CAG(14 : 0) and CAG(19c : 0), which are predominant in *H. pylori*. (C) Relative levels of CPGs containing different fatty acid chains in *H. pylori* 26695 (St) and the clinical strains isolated from patients with gastric cancer (GC1–CG4). “Others” represents the total of minor CPGs, including CPG(18 : 0/19c : 0), CPG(19c : 0/19c : 0), CPG(18 : 0/18 : 0), CPG(16 : 0/18 : 1) and CPG(18 : 1/18 : 1). Data shown are from three biological replicates. Error bars represent the standard deviations. All statistically significant differences are indicated with asterisks; *P < 0.05, **P < 0.01, or ***P < 0.001 based on Student's *t* test.

We further examined the CAG and CPG compositions when the AGS cells were co-cultured with different strains of *H. pylori*, including the standard type (26695) and four clinical strains isolated from patients with gastric cancer (GC1–GC4). UPLC-MS analysis indicated that on average 83% of the total CAGs obtained from the clinical isolates had incorporated human fatty acid chains, including (16 : 0), (18 : 0), (18 : 1), (18 : 2) and (20 : 4). In contrast, on average only 52% of CAGs from *H. pylori* 26 695 showed such incorporation (Fig. 5A). In particular, the GC1–GC4 strains contained a higher content of CAG(18 : 1), CAG(18 : 2) and CAG(20 : 4) (Fig. 5B), which have one or more double bonds. There was no obvious difference in the lipid composition of CPGs among any of these *H. pylori* strains (Fig. 5C).

To search for a possible precursor in the biosynthesis, we first treated *H. pylori* cultures with deuterium-labelled PE, phosphatidylcholine (PC) or palmitic acid (16 : 0) for 7 h to examine the incorporation of deuterium-labelled acyl chains in CAGs and CPGs. PE was found to be the major precursor in the biosynthesis of CAGs and CPGs, although PC was also incorporated into CAGs to a small extent (Fig. 4C). Next, AGS cells were pretreated with **1** for 24 h, and incubated with deuterium-labelled PE or PC (the two major phospholipids in human cells) for 1 h. Because it is important to rule out the possibility of adsorbing any lipids from culture dishes during the *H. pylori* infection process, we extensively washed the phospholipid-pretreated AGS cells with phosphate-buffered saline (PBS) and then transferred the cells to new plates. The incorporation of each phospholipid into AGS cells was monitored by UPLC-MS. The cells were subsequently infected with *H. pylori* 26695 for 6 h and then analysed by UPLC-MS to determine the proportion of deuterium-labelled CAGs and CPGs. The analysis supported the previous observation and demonstrated PE to be the primary source for the acyl chains of CAGs and CPGs (Fig. 4D), and also showed that the bacteria can obtain phospholipids from the host cell for the synthesis of CAGs and CPGs. CPGs appeared to have a much lower level of incorporation of the host phospholipids than the CAGs (Fig. S7C and D†).

### Enhanced CagA translocation and lipid raft formation by CAGs containing longer acyl chains

**Fig. 6:**
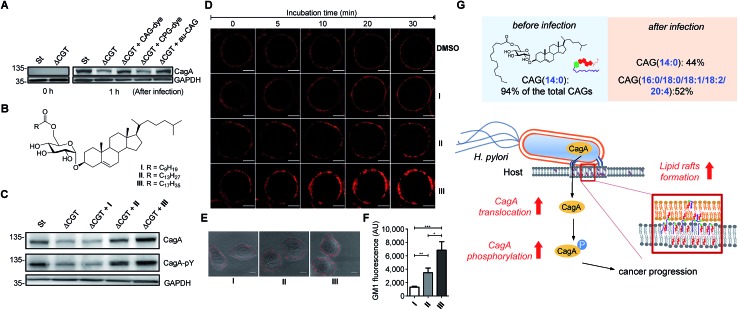
Extent of CagA translocation and lipid raft formation under various conditions. (A) CagA translocation in AGS cells cultured with *H. pylori* 26695 (St), a cholesterol glucosyltransferase-deficient strain (ΔCGT), or ΔCGT pretreated for 30 min with fluorescently-labelled CAG(14 : 0) (CAG-dye), fluorescently-labelled CPG(14 : 0/19c : 0) (CPG-dye) or authentic CAG(14 : 0) (au-CAG). Translocated CagA was detected by immunoblotting before and 1 h after infection. (B) Structures of CAGs containing an O6′ ester of decanoic acid (**I**), myristic acid (**II**) and stearic acid (**III**). **I** carries the shortest fatty acid chain; while **II** and **III** carry the most abundant fatty acid chains in *H. pylori* and humans, respectively. (C) Effects of CAGs with different acyl chains on CagA translocation and CagA tyrosine phosphorylation as detected by immunoblotting after the co-culture of AGS cells with *H. pylori* strains for 4 h as described in (A). (D) Time-lapse images of AGS cells treated with CAGs (**I**, **II** or **III**). Lipid rafts (GM1) in AGS cells were first labelled with Alexa Fluor 594-conjugated cholera toxin subunit β, followed by treatment with the indicated CAGs (10 : 0, 14 : 0 or 18 : 0) at 37 °C. Time-lapse images were collected under a Zeiss LSM 510 confocal microscope at 0, 5, 10, 20 and 30 min. Scale bars, 5 μm. (E) Representative confocal immunofluorescence images for AGS cells that were first treated with CAGs (**I**, **II** or **III**) for 30 min prior to the labelling of GM1. Scale bars, 10 μm. (F) Quantitation of the fluorescent intensities measured in (E). At least 40 GM1-positive AGS cells from each treatment were scored for the quantitative analysis using ImageJ software. Statistical significance was evaluated using Student's *t*-test (*P < 0.05, **P < 0.01 or ***P < 0.001). (G) The figure demonstrates how the uptake of human lipids enhances bacterial virulence. The resulting uptake altered the bacterial CAG compositions (especially CAGs containing longer or/and unsaturated fatty acid chains), leading to a higher level of CagA translocation and lipid raft formation.

Because we were able to isolate individual CAGs and CPGs, we further investigated the degree of influence of each derivative on the physiological activities. In the study of CagA translocation, we compared *H. pylori* 26695 to ΔCGT (a cholesterol glucosyltransferase-deficient strain (prepared as previously reported^16^)) and to ΔCGT pretreated for 30 min with either authentic CAG(14 : 0) (synthesised as previously reported^34^), fluorophore-labelled CAG(14 : 0) or fluorophore-labelled CPG(14 : 0/19c : 0) (Fig. 6A). CAG(14 : 0) and CPG(14 : 0/19c : 0) are the most abundant CAG and CPG in *H. pylori*, respectively. AGS cells were infected with the *H. pylori* strains for 1 h, and then the cell extracts were immunoblotted with a CagA-specific monoclonal antibody. There was no obvious difference between pretreatment with labelled and authentic CAG, indicating that fluorophore-conjugated CGd analogues are comparable to the corresponding native CGds and could be purified individually for further investigation. Additionally, CAG was found, for the first time, to promote a higher degree of CagA translocation than CPG (Fig. 6B). The result with authentic CAG was similar to that of the positive control (*H. pylori* 26695), indicating that CAGs are likely responsible for the observed activity among the mixture of CGds.

We further investigated the effect of chain length on the activity of CAGs. ΔCGT was pretreated for 30 min with three CAGs of increasing chain length (CAG(10 : 0), CAG(14 : 0) and CAG(18 : 0); Fig. 6B) and used to infect AGS cells. Analysis of CagA translocation and the subsequent tyrosine phosphorylation^35^ demonstrated that the acyl chain length of CAGs indeed plays a critical role in these activities (Fig. 6C). The degree of CagA translocation and the corresponding phosphorylation increased with the increasing chain length.

In addition, we examined whether the chain length of CAGs correlates with lipid raft formation. Time-lapse fluorescence microscopy was performed to monitor the mobilisation of ganglioside GM1-associated rafts after each CAG was added to the AGS cells. GM1 in the AGS cells was labelled with Alexa-594-cholera toxin prior to treatment with CAG(10 : 0), CAG(14 : 0) or CAG(18 : 0). We found a time-dependent increase in fluorescence intensity for all three CAGs, and further found that CAG(18 : 0) induced the greatest accumulation of GM1 (Fig. 6D and ESI movies†). Furthermore, when AGS cells were treated with CAG(10 : 0), CAG(14 : 0) or CAG(18 : 0) for 30 min and then labelled with GM1, the GM1 fluorescence intensity increased with increasing chain length (Fig. 6E and F). Taken together, these results suggest that CAGs with incorporated human fatty acids enhance the formation of lipid rafts in the membrane, which may lead to higher levels of CagA translocation and subsequent phosphorylation.

## Discussion

Click chemistry-based tagging methods have been prevalently used in metabolite analysis. Our development appears to provide several advantages. First, the introduced fluorophore MAN is readily protonated in solution, significantly enhancing the ionisation level in mass spectrometry (10^2^ to 10^5^-fold, depending on the type of CGds). The combined mass spectrometric detection and enhanced ionisation of tagged metabolites made it possible to reach the detection limit at the femtomolar level. Second, MAN was found to display a much higher stability in comparison with several commercial fluorescent dyes (Fig. S2B†). The beneficial features of high sensitivity and stability support the idea that MAN should be useful for other profiling studies. Third, conventional analysis (*e.g.* 2D thin-layer chromatography) is usually entangled with contamination from phospholipids, explaining the reason why the relevant isolation or purification becomes troublesome. In contrast, our development makes the entire process more robust and it takes 2–3 weeks to obtain a single cholestyl glucosde derivative at the mg level, including 7–10 days of culturing the bacteria. After harvest, any of CGds can be made readily available with >95% purity after the bacterial cells are subjected to extraction with organic solvents, followed by two steps of column chromatography. Furthermore, the use of azide-containing compound **1** and the fluorophore MAN is compatible for the purpose of fluorescent imaging to demonstrate how these labelled CGds are translocated to and/or distributed in the host cell.

Previous findings on the uptake of l-fucose from the host by *H. pylori* indicate that this is a beneficial strategy to acquire additional carbon and energy, as well as to evade host immune surveillance.^10^ Likewise, *H. pylori* has evolved to derive various CGds from exogenous cholesterol, for which it is auxotrophic (lacking biosynthetic machinery), but this is indispensable for its pathogenesis and virulence.^3^,^11^,^14^ In this study, by using a specific labelling method, we demonstrated that *H. pylori* also acquires phospholipids from the host for CAG biosynthesis. The acyl chain composition of *H. pylori* CAGs dramatically changed in the presence of human host cells. For instance, CAG(14 : 0) constituted 94% of the total CAGs when bacteria were cultured alone, whereas when bacteria were cultured with human cells, CAG(14 : 0) was reduced to 44% and was accompanied by CAGs containing human fatty acid chains (52%; (16 : 0), (18 : 0), (18 : 1), (18 : 2) and (20 : 4); Fig. 4A and S7A†). This alteration led to a higher level of CagA translocation, indicating that accessing human phospholipids enhances bacterial virulence by altering the bacterial CAG compositions (Fig. 6G). Interestingly, Hatakeyama *et al.* found that host phosphatidylserine is aberrantly externalised from the inner to the outer leaflet of the host plasma membrane at the site of *H. pylori* attachment, which facilitates the delivery, intracellular localisation and pathophysiological activity of CagA.^36^ This finding suggests a mechanism for the release of host phospholipids during bacterial infection.^36^

Cholesterol is an important component of cell membranes. Particularly in lipid rafts, this molecule serves as a glue to maintain the close packing of lipids and proteins. *H. pylori* CGds can be considered modified versions of cholesterol with the additional attachment of glucose, acyl and/or phosphatidyl groups. CAGs, CPGs and lyso-CPGs likely play different roles in membranes. Wang *et al.* reported that CagA translocation is mediated in a cholesterol-dependent manner, as the formation of CGds from host cholesterol promotes the translocation of CagA by affecting the membrane dynamics and enhancing lipid raft formation.^11^,^16^ Our study identified CAGs, but not CPGs, as being responsible for the enhanced translocation of CagA and for the formation of lipid rafts. An altered CAG composition therefore has multifaceted effects. Lipid rafts have been demonstrated to serve as the obligate structure for the entry of bacterial and viral pathogens.^37^,^38^

Our findings further demonstrate that only CAGs with longer acyl chains exert this effect to promote lipid raft formation and thus CagA translocation. This result is consistent with a number of previous reports. For instance, lipid rafts containing palmitoylated or stearoylated (C16/C18) GPI-anchored variant surface glycoprotein display a higher resistance to Triton X-100 extraction than those containing myristoylated (C14) GPI-anchored variant surface glycoprotein.^39^,^40^ Several raft-associated proteins are palmitoylated.^41^,^42^ When cytoplasmic proteins are subjected to lipidation, the presence of at least one palmitate chain, rather than the shorter myristoylate, is required for raft association.^40^,^43^ These studies support the idea that acquired host CAG(16 : 0) and CAG(18 : 0), rather than the plentiful bacterial CAG(14 : 0), assist in the formation of lipid rafts to enhance the subsequent CagA translocation.

We also demonstrated that bacterial strains obtained from patients with gastric cancer displayed a significant increase in CAGs carrying unsaturated acyl chains, *i.e.* CAG(18 : 1), CAG(18 : 2) and CAG(20 : 4). Brewster and Safran demonstrated that hybrid lipids (comprising one fully saturated and one partially unsaturated chain) serve as a surface-active component by occupying the interface between the lipid raft (facing the saturated anchor) and the less-ordered environment (facing the unsaturated chain).^44^ These reports suggest that the unsaturated chains of CAG(18 : 1), CAG(18 : 2) and CAG(20 : 4) may contribute to the raft structure by interacting with less-ordered lipids.

Steryl glycosides (SGs) are known to prevalently exist in many organisms and some of SGs are also acylated at the C6′ position of the sugar moiety, like CAGs in *H. pylori*. In general, there are two possible routes regarding enzymatic acyl transfer: one requires acyl-CoA as the donor, while the other needs a different substrate for the same purpose. The acylation of several plant steryl glucosides were found to be independent of acyl-CoA. Instead, phosphoglycerolipids or/and galactolipids (fatty acids attached to C1 and C2) were employed for the enzymatic transfer of fatty acids.^45^ In AGS cells, PE and PC are the major phospholipids. PE is also the major phospholipid in *H. pylori*. To search for a possible precursor in the biosynthesis of CAG, PE, PC and fatty acid (PMA) were individually added to the bacterial culture to examine the incorporation of exogenous acyl chains (*i.e.* deuterium-labelled acyl chains in CAG). As shown in Fig. 4C, PE was found to be the major precursor in the biosynthesis of CAG, while little or no PMA was incorporated into the CAGs. This result supports there being a lipid-dependent acyltransferase involved in the biosynthesis of CAGs, and that PE is a better substrate for this enzyme.

To explain the observed uptake of human phospholipids, it appears to be essential to understand where the discussed acyltransferase is located in *H. pylori*, since the incorporation of deuterium-labelled acyl chain primarily occurred in CAGs but not in CPGs. We propose that the CAG-related enzyme resides at the bacterial outer membrane, making the direct access of human phospholipids possible. As a matter of fact, several studies have suggested that the biosynthetic location of CAGs is indispensable for their release to the surface of host cells and for the subsequent induction of the cytokines TNF-α and INF-γ.^18^ On the other hand, the unknown phosphatidyltransferase, to convert CG to CPG, might be located at either the inner membrane or at the cytoplasm, which would hamper the access of the host phospholipids.

## Conclusions

We established a metabolite-tagging method for the robust detection of CGds at femtomolar sensitivity. The quantitative analysis of CGds indicated that these bacteria utilise phospholipids from human host cells to produce cholesteryl 6′-acyl α-glucosides (CAGs) containing longer/unsaturated fatty acid chains. Our findings demonstrate, for the first time to the best of our knowledge, that phospholipid hijack is the key to augment the pathogenicity. Because the incorporated fluorophore displayed great inherent stability and a high ionisation degree in mass spectrometry, the development should be applicable to other metabolic profiling.

## Experimental

### Metabolic labelling of CGds

*H. pylori* cells were seeded on Brucella agar plates containing **1** (50 μM) for 2–4 days. Bacterial lipids were extracted by the Folch method,^26^ redissolved in chloroform/methanol/water (5 : 4 : 1, v/v/v) and subjected to a click reaction with 0.25 mM MAN in the presence of 1.25 mM TBTA, 12.5 mM sodium ascorbate and 0.25 mM CuSO_4_ at room temperature for 1 h. The solvent was removed *in vacuo*, and the dried residue was redissolved in chloroform/methanol/water (5 : 4 : 1, v/v/v) and analysed by HPLC and UPLC-MS.

### HPLC analysis

For the experiments described in Fig. 2A, an analytical Inspire C18 column (5 μm, 4.6 × 250 mm) was used at a flow rate of 0.4 mL min^–1^. To obtain fluorophore-labelled CAG(14 : 0) and CPG(14 : 0/19c : 0) for NMR characterisation, a preparative Inspire C18 column (5 μm, 21.5 × 250 mm) was used at a flow rate of 6.5 mL min^–1^. For quantification of the CGds using HPLC-fluorescence, an analytical Inspire C18 column (5 μm, 4.6 × 250 mm) was used at a flow rate of 0.4 mL min^–1^. In all the experiments, eluent A contained 30% acetonitrile and 70% aqueous 20 mM ammonium acetate, while eluent B contained 90% isopropyl alcohol and 10% aqueous 20 mM ammonium acetate.

### UPLC-MS analysis

UPLC-MS analysis was performed on a linear ion trap-Orbitrap mass spectrometer (Orbitrap Elite; Thermo Fisher Scientific) coupled online with a UPLC system (ACQUITY UPLC; Waters). The mass spectrometer was operated in the positive electrospray ion mode set to one full FT-MS scan (*m*/*z* 200–1600; 15 000 resolution) and switched to different FT-MS product ion scans (15 000 resolution) for the different CGd precursors. The CGds were separated on a CSH C18 column (1.7 μm, 100 × 2.1 mm; Waters, USA) using gradients of LC buffer A (10 mM ammonium acetate in 40 : 60 [v/v] acetonitrile/water) and LC buffer B (10 mM ammonium acetate in 90 : 10 [v/v] isopropyl alcohol/acetonitrile) at a flow rate of 0.4 mL min^–1^. To normalise the variations in sample preparation and UPLC-MS analysis, phosphatidylinositol containing deuterium-labelled palmitic acid (16 : 0) and unlabelled oleic acid (18 : 1) was used as the internal standard and added to the bacterial lysate (10^6^ bacteria, determined by optical density at 600 nm). To determine the ionisation efficiency of the CGds with or without MAN labelling, the normalised peak area was defined as the peak area of CGds divided by the peak area of the internal standard. To quantify the MAN-labelled CGds from the bacterial lysates, the peak area of selected specific fragments for the extracted ion chromatograms was used. To establish the calibration curves of CGds, we added purified MAN-labelled cholesterol, CG, CAG(14 : 0), CPG(14 : 0/19c : 0) and Lyso-CPG(19c : 0) to the lysate from 10^6^ bacteria along with the click chemistry reactants (TBTA, sodium ascorbate and CuSO_4_). The calibration curves for quantitation were obtained by analysing the peak areas of the serially diluted standard (0.088, 0.176, 0.35, 0.7, 1.4, 2.8, 5.6, 11.25, 22.5, 45 and 90 pmol per 10^6^ bacteria) measured by UPLC-MS operated in the product ion scan mode. The amount of CGds in the bacterial lysate was determined by comparing the peak area in lysate to the standard curves. The relative isotopic abundance error was calculated as previously described.^27^

### Co-culture experiments

Co-culture experiments were performed as previously described.^16^ In the experiments described in Fig. 4A, B and 5, AGS cells (8.0 × 10^6^) were seeded in a 10 cm tissue culture dish and treated with **1** (6.2 μg mL^–1^) in Dulbecco's Modified Eagle Medium (DMEM) (Gibco, Invitrogen) at 37 °C for 16 h. AGS cells were infected with *H. pylori* (26695 or clinically isolated strains) at an MOI of 200 : 1 for 6 h and subsequently collected by centrifugation (6000 × *g*) for 5 min. CGds-containing lipids were isolated by Folch extraction and fluorescently labelled by click reaction for UPLC-MS analysis. With respect to the study shown in Fig. 4D, AGS cells (8.0 × 10^6^) were seeded in a 10 cm tissue culture dish and treated with **1** (6.2 μg mL^–1^) in DMEM at 37 °C for 16 h. To label AGS cells with deuterium-labelled phospholipid, the AGS cells were incubated with serum-free medium (Ham's F12, Invitrogen) containing 0.1 mM 1-palmitoyl(d31)-2-oleoyl-*sn*-glycero-3-phosphoethanolamine (dPOPE; 16 : 0 d31 : (18 : 1)) or 0.1 mM 1-palmitoyl(d31)-2-oleoyl-*sn*-glycero-3-phosphocholine (dPOPC; 16 : 0 d31 : (18 : 1)) (Avanti Polar Lipids, USA) at 37 °C for 1 h. It is important to wash the cells with PBS five times to remove unbound **1** and dPOPE or dPOPC. Then the cells were transferred to a new plate for another 12 h. To demonstrate that the bacterial uptake of deuterium-labelled phospholipids was from the cells rather than the phospholipids remaining on the culture dish, empty culture dishes were first treated with **1** for 16 h, treated with POPE or dPOPC for another 1 h, washed with PBS five times, and then used to culture *H. pylori* without AGS cells for 6 h. No deuterium-labelled CGds were produced by *H. pylori* under these conditions. To perform the infection experiments, unloaded or dPOPE/dPOPC-loaded AGS cells were infected with *H. pylori* (26695) at an MOI of 200 : 1 for 6 h and subsequently collected by centrifugation (6000 × *g*) for 5 min. CGds-containing lipids were isolated by Folch extraction and fluorescently labelled by click reaction for UPLC-MS analysis. For the CagA translocation experiments, AGS cells (8.0 × 10^5^) were seeded in DMEM (Gibco, Invitrogen) in a 6-well culture plate at 37 °C for 16 h. To load *H. pylori* cells with CGds, *H. pylori* cells (26695 or ΔCGT) were incubated with serum-free medium (Ham's F12) containing 30 μM of MAN-labelled CAG(14 : 0), MAN-labelled CPG(14 : 0/19c : 0) or unlabelled CAG standard at 37 °C for 30 min. The bacterial cells were then washed with PBS to remove unbound CPG or CAG and then used to infect AGS cells at an MOI of 200 : 1 for 1 h.

### Immunoprecipitation and blotting

After the infection of AGS cells with *H. pylori* (MOI of ∼200 : 1), the cells were washed three times with PBS and collected with a cell scraper. To detect the internalised CagA to AGS cells, saponin was used to lyse the AGS cells, but with care to keep the bacteria intact.^5^,^35^ The AGS cells were gently lysed with PBS containing 0.1% saponin, protease inhibitor cocktail (Calbiochem, USA) and phosphatase inhibitor cocktail (Calbiochem, USA) for 10 min at room temperature. The cell debris and intact bacteria were separated from the soluble fraction by centrifugation (6000 × *g* for 5 min), and the solute was filtered with a 0.22 μm-pore filter to obtain human cell proteins and the internalised CagA. The saponin lysis procedure was also performed on standard type *H. pylori* (26695) alone. Subsequent immunoprecipitation and blotting of CagA indicated that no CagA was detected, supporting the idea that this procedure did not release cytoplasmic proteins from *H. pylori* (data not shown). The filtrate (100 μg) was immunoprecipitated using 10 μg rabbit anti-CagA polyclonal antibody (Austral Biologicals, USA) and 30 μl Protein A Plus Agarose (Thermo scientific, USA) at 4 °C overnight. The obtained precipitates were washed three times with immunoprecipitation buffer (25 mM Tris–HCl (pH 7.4), 150 mM NaCl, 5 mM EDTA, 1% Triton X-100) and once with TBS buffer (50 mM Tris–HCl, pH 7.4, 150 mM NaCl, 1 mM CaCl_2_), then boiled in PAGE sample buffer (62.5 mM Tris–HCl, pH 6.8, 2% SDS, 10% (v/v) glycerol, 0.05% brilliant blue R) at 95 °C for 10 min, resolved by 6% SDS-PAGE and then transferred onto polyvinylidene difluoride membranes (Millipore, USA). The membranes were blocked with 5% (w/v) skimmed milk in TBS buffer containing 0.01% Tween 20 at room temperature for 1 h and incubated overnight with mouse monoclonal anti-CagA (Austral Biologicals; 1 : 2000), mouse monoclonal anti-phosphotyrosine (4G10, Millipore; 1 : 2000) or mouse monoclonal anti-GAPDH (ab9482, Abcam, UK; 1 : 5000) at 4 °C. The blots were washed and incubated with HRP-conjugated secondary antibodies (Santa Cruz Biotechnology, USA) at a 1 : 5000 dilution, and the proteins of interest were visualised using an enhanced chemiluminescence assay (WBKLS0500, Millipore). See the ESI† for additional information.

## Supplementary Material

Supplementary information
