# Supplementary material for: Metabolic labelling of cholesteryl glucosides in Helicobacter pylori reveals how the uptake of human lipids enhances bacterial virulence
Source: Chem Sci. 2016 Jun 3;7(9):6208–16. doi: 10.1039/c6sc00889e (PMC6024656; doi:10.1039/c6sc00889e)
Supplement: Supplementary file 1 [file SC-007-C6SC00889E-s001.zip › C6SC00889E.pdf]

Supplementary Information

Metabolic labeling of cholesteryl glucosides in *Helicobacter pylori* reveals how uptake of human lipids enhances bacterial virulence

*Corresponding author: Chun-Hung Lin, Institute of Biological Chemistry, Academia Sinica, No. 128 Academia Road Section 2, Nan-Kang, Taipei, 11529, Taiwan, Tel: 886-933877327, E-mail: chunhung@gate.sinica.edu.tw

Contents

General reagents and instruments of chemical synthesis..........................3
Supplementary schemes and synthetic procedures...4-9
Supplementary spectra data...10-16
Supplementary methods...17-18
Supplementary figures...19-38
Supplementary tables..39-41
Supplementary movies...42
Reference...43

General reagents and instruments of chemical synthesis

All chemicals were purchased from Acros, Fluka, Sigma or Alfa Aesar, and were used without further purification unless specified. All reactions were performed under a nitrogen atmosphere in oven-dried glassware unless indicated. Anhydrous solvents were prepared as follows: Tetrahydrofuran (THF), Ethanol (EtOH) and *N,N*-Dimethylformamide (DMF) were predried by freshly activated 4 Å Molecular Sieves and used directly. Reaction products were purified by column chromatography with Merck silica 60 (230-400 mesh). Thin layer chromatography (TLC) was used to monitor all reaction progresses by Merck silica gel 60 F_{254} analytical plates. Compounds were first measured by UV lamp and visualized by staining reagents that contained phosphomolybdic acid hydrate.

NMR spectra were recorded routinely by a Bruker AV-400 (400 MHz), AVII-500 (500 MHz), AVANCE III 600 (600 MHz), or AVANCE III 850 (850 MHz) spectrometer using tetramethylsilane (δ_H 0.00), $CDCl_3$ (δ_H 7.26) or CD_3OD [δ_H 3.31 (central line of a quintet)] as the internal standard. ^{13}C NMR spectra were recorded by a Bruker AV-400 (100 MHz), AVII-500 (125 MHz), or AVANCE III 600 (150 MHz) spectrometer with $CDCl_3$ [δ_C 77.2 (central line of a triplet)] as the internal standard. 2D NMR experiments (including ^1H-^1H COSY, ^1H-^1H TOCSY, ^1H-^{13}C HMQC, and ^1H-^{13}C HMBC) were acquired by a Bruker AVII-500 or AVANCE III 600 spectrometer. High resolution mass spectra were obtained by Bruker Bio-TOF III (ESI-TOF) or Bruker Ultraflex (MALDI-TOF/TOF) and were reported as mass/charge (m/z) with percentage of relative abundance. Optical rotations were measured at 589 nm (sodium D-line) at 25 °C with the PerkinElmer Model 341 Polarimeter. Specific rotations were reported as $[\alpha]_D^{25}$ after dividing the observed values by the sample concentration (C, in g·mL^{-1}) and the path length (l, in dm).

Supplementary scheme 1

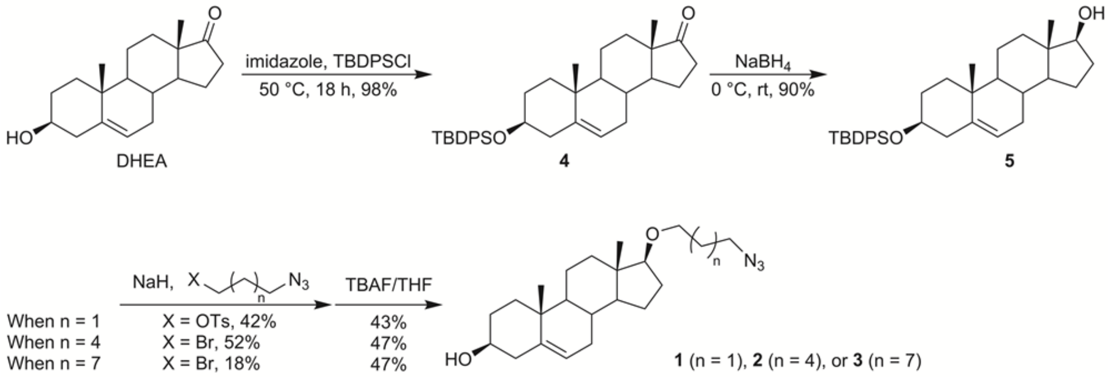

Three different azide-containing cholesterol analogues, 17β-([3"-azidopropoxy)-5-androsten-3β -ol (**1**), 17β-([6"-azidohexanoxy)-5- androsten-3β-ol (**2**) and 17β-([9"- azidononanoxyl)-5-androsten-3β-ol (**3**), were synthesized from dehydroepiandrosterone (DHEA). The only difference among the three products is the substituent length at O17. Detailed synthetic procedures were described as follows.

3β-(*tert*-Butyldiphenylsilyloxy)-5-androsten-17-one (4)

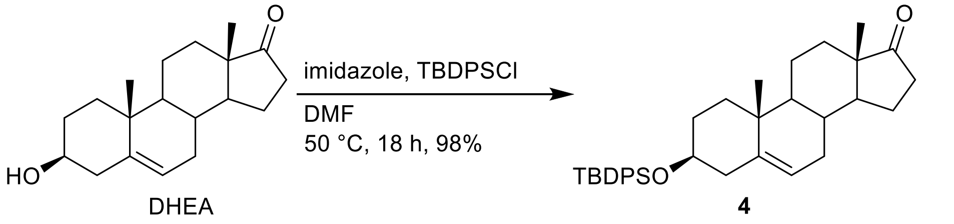

To a solution of DHEA (1.0 g, 3.46 mmol) in dry DMF (30 mL) was added imidazole (590 mg, 8.64 mmol) at 0 °C. The resulting mixture was allowed to stir at 0 °C for 15 min followed by addition of *tert*-butyldiphenylsilyl chloride (TBDPSCl) (2.2 mL, 8.64 mmol). The ice bath was removed and the reaction mixture was heated at 50 °C for 18 h. The reaction was quenched by addition of saturated aqueous NH_4Cl at 0 °C, and the resulting mixture was rigorously stirred for additional 30 min at room temperature. The solvent was removed *in vacuo* to give the dried residue that was diluted with ethyl acetate. The resulting solution was washed with water and brine sequentially, dried over $MgSO_4$, filtrated and concentrated *in vacuo* to afford the crude that was purified by recrystallization form methanol and petroleum ether (40-60 °C) to yield the desired compound **4** (1.79 g, 98%) as a white solid. TLC (ethyl acetate/hexanes = 25/75, v/v): R_f= 0.85; $[\alpha]_D^{25}$ = 30.7 (*c* = 0.053, $CHCl_3$); ^{1}H NMR (400 MHz, $CDCl_3$) δ 7.65-7.67 (m, 4H, Ar*H*), 7.35-7.38 (m, 6H, Ar*H*), 5.13 (d, *J* = 5.0 Hz, 1H, H-6), 3.47-3.54 (m, 1H, H-3), 1.05 (s, 9H, $(CH_3)_3$C-Si), 1.00 (s, 3H, C*H*$_3$), 0.88 (s, 3H, C*H*$_3$); ^{13}C NMR (100 MHz, $CDCl_3$) δ 221.4, 141.8, 135.0, 129.7, 127.9, 120.6, 73.3, 52.0, 50.4, 47.7, 42.7, 37.4, 36.8, 36.0, 32.0, 31.6, 31.0, 27.2, 26.8, 22.1, 20.5, 19.7, 19.4, 13.7; HRMS (*m/z*): [M+H]· calcd. for $C_{35}H_{47}O_2Si^+$, 527.3340; found, 527.3328.

3β-(*tert*-Butyldiphenylsilyloxy)-17β-hydroxy-5-androstene (5)

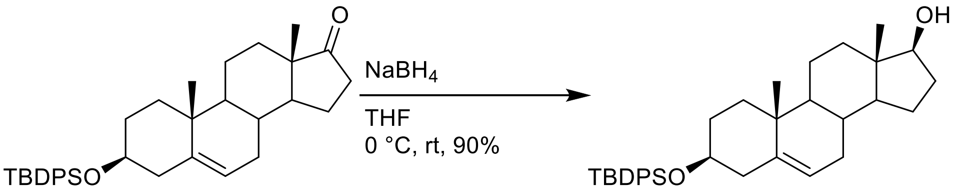

To a solution of compound **4** (1.0 g, 1.90 mmol) in methanol (28 mL) was added sodium borohydride (109 mg, 2.88 mmol) at 0 °C. The reaction mixture was stirred for an additional 30 min at room temperature. The resulting mixture was quenched by addition of water at 0 °C with rigorously stirring. The solvent was removed *in vacuo* to give dried residue that was diluted with ethyl acetate. The resulting mixture was washed with water and brine sequentially, dried over $MgSO_4$, filtrated and concentrated *in vacuo* to afford the crude that was purified by recrystallization form methanol at room temperature to yield the desired compound **5** (903 mg, 90%) as a white solid. TLC (ethyl acetate/hexanes = 25/75, v/v): R_f= 0.5; $[\alpha]_D^{25}$ = 39.0 (*c* = 0.053, $CHCl_3$); ^{1}H NMR (400 MHz, $CDCl_3$) δ 7.65-7.67 (m, 4H, Ar*H*), 7.35-7.38 (m, 6H, Ar*H*), 5.11 (d, *J* = 4.97 Hz, 1H, H-6), 3.57 (t, *J* = 9.04 Hz, 1H, H-17) 3.57-3.47 (m, 1H, H-3), 1.06 (s, 9H, $(CH_3)_3$C-Si), 0.99 (s, 3H, C*H*$_3$), 0.71 (s, 3H, C*H*$_3$); ^{13}C NMR (100 MHz, $CDCl_3$) δ 141.5, 135.9, 134.9, 129.6, 127.6, 121.0, 81.9, 77.4, 73.3, 51.5, 50.3, 42.8, 42.7, 37.4, 36.7, 32.1, 31.6, 30.5, 27.2, 23.6, 20.8, 20.8, 19.6, 19.3, 11.1; HRMS (*m/z*): [M+H]· calcd. for $C_{35}H_{49}O_2Si^+$, 529.3496; found, 529.3478. The stereochemistry of C-17 in compound **5** was determined by the NOESY spectrum shown as follows. (A) Structure of compound 5. (B) NOESY spectrum of **5**. The arrow designates the cross-peak between H-17 and H-14.

A

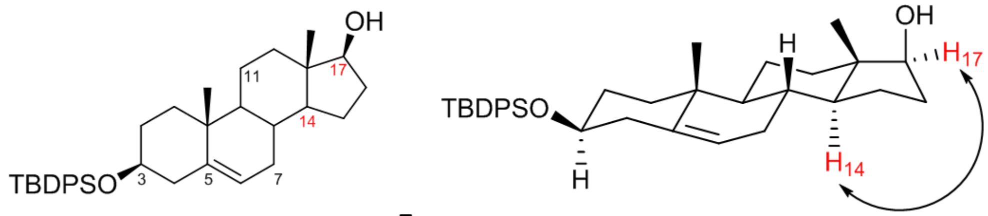
OH
17
11
14
TBDPSO
3
5
7
H
H
TBDPSO
H_{17}
H_{14}

5

B

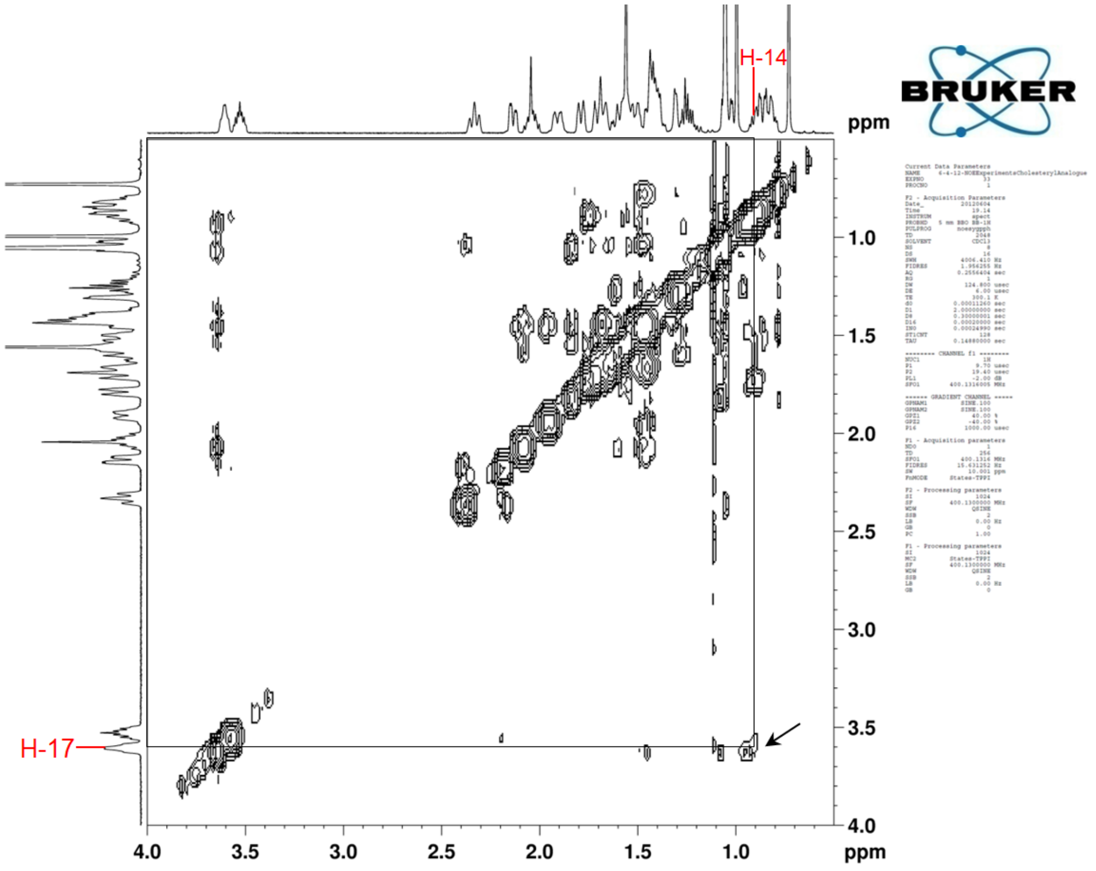
H-14
H-17
ppm
1.0
1.5
2.0
2.5
3.0
3.5
4.0
4.0
3.5
3.0
2.5
2.0
1.5
1.0
ppm

17β-(3’-azidopropoxy)-5-androsten-3β-ol (1)

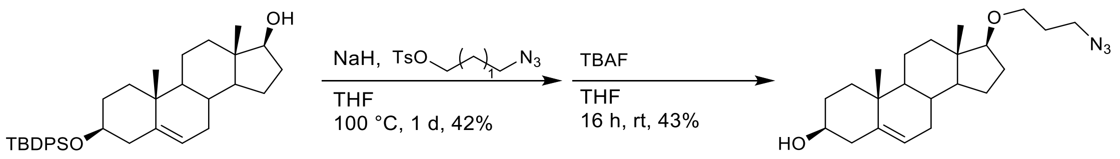

5 **1**

To a solution of dried compound **5** (1.0 g, 1.89 mmol) in THF (30 mL) was added sodium hydride (150 mg, 6.24 mmol) at room temperature. The mixture was stirred under argon at room temperature until it became a clear solution (7 h). To this clear solution was added 3-azidopropyl 4-methylbenzenesulfonate (1.45 g, 6.24 mmol). The reaction temperature was raised to 100 °C and the mixture was allowed to stir at 100 °C for 24 h. The reaction was cooled to room temperature and then quenched by addition of saturated aqueous NH_4Cl at 0 °C with rigorously stirring. Most solvent was removed *in vacuo* to afford dried residue that was diluted with dichloromethane and the resulting mixture washed with water and brine sequentially, dried over $MgSO_4$, filtrated and concentrated *in vacuo* to afford the crude that was purified by silica gel column chromatography with ether/petroleum ether (2/8, v/v) to afford the pure TBDPS-protected product. TLC (ethyl acetate/hexanes = 15/85, v/v): R_f= 0.65; The product (588.6 mg, 0.79 mmol) was then dissolved in anhydrous THF (15 mL) and treated with a solution of (*n*-Bu)$_4$NF (1.0 M solution in THF, 7.9 mL, 7.9 mmol) at room temperature, and the resulting mixture was stirred for 16 h. The reaction was quenched with saturated aqueous NH_4Cl. The solvent was removed *in vacuo* to afford the dried residue that was diluted with ethyl acetate and the resulting mixture was washed with water and brine sequentially, dried over $MgSO_4$, filtrated and concentrated *in vacuo* to afford the crude that was purified by flash column chromatography with dichloromethane/ethyl acetate (9/1, v/v) to afford pure 17β-(3’-azidopropoxy)-5-androsten-3β-ol (**1**) as a white solid (127.6 mg, 18 % in two steps). TLC (ethyl acetate/hexanes = 15/85, v/v): R_f= 0.20; $[\alpha]_D^{25}$ = −73.6 (*c* = 0.037, $CHCl_3$); ^{1}H NMR (400 MHz, $CDCl_3$) δ 5.35 (d, *J* = 5.3 Hz, 1H, H-6), 3.56-3.47 (m, 3H, H-3 + H-20a,b), 3.39 (t, *J* = 6.7 Hz, 2H, H-22a,b), 3.29 (t, *J* = 8.4 Hz, 1H, H-17), 2.33-2.19 (m, 2H, H-4a + H-4b), 2.04-1.96 (m, 2H, H-16a +H-7a), 1.94-1,84 (m, 3H, H-12a + H-11a + H-15a), 1.82 (m, 1H, H-2a), 1.83-1.78 (dd, *J* = 12.9 Hz, *J* = 6.7 Hz, 2H, H-21a,b), 1.62-1.04 (m, 8H, H-1a + H-2b + H-8 + H-16b + H-11b + H-7b + H-15b + H-12b), 1.02 (s, 3H, C19H_3), 0.99-0.86 (m, 3H, H-14 + H-1b + H-9), 0.76 (s, 3H, C18H_3); ^{13}C NMR (100 MHz, $CDCl_3$) δ 141.1, 121.6, 89.5, 72.0, 66.6, 51.8, 50.5, 48.8, 43.1, 42.5, 38.1, 37.5, 36.8, 32.0, 31.9, 31.7, 29.8, 28.2, 23.7, 21.0, 19.7, 11.7; HRMS (*m/z*): [M+H]· calcd. for $C_{22}H_{36}N_3O_2^+$, 374.2802; found, 374.2796

17β-(6'-azidohexanoxy)-5-androsten-3β-ol (2)

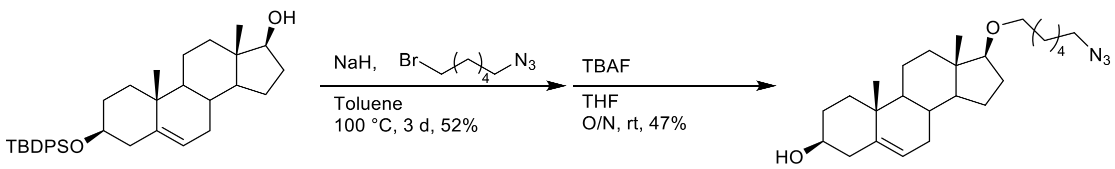

5 **2**

The preparation of **2** took two steps starting from **5** (24% total yield), which is similar to that of **1**. TLC of **2** (ethyl acetate/hexanes = 15/85, v/v): R_f= 0.20; $[\alpha]_D^{25}$ = –79.5 (c = 0.042, $CHCl_3$); ^{1}H NMR (500 MHz, $CDCl_3$) δ 5.34 (d, J = 5.2 Hz, 1H, H-6), 3.51 (m, 1H, H-3) 3.48-3.38 (m, 2H, H-20a,b), 3.28 (t, J = 8.4 Hz, 1H, H-17), 3.26 (t, J = 6.7 Hz, 2H, H-25a,b), 2.31-2.20 (m, 2H, H-4a + H-4b), 2.02-1.95 (m, 2H, H-16a +H-7a), 1.94-1.89 (m, 1H, H-12a), 1.87-1.80 (m, 3H, H-11a + H-15a + H-2a), 1.64-1.43 (m, 10H, H-21a,b + H-24a,b + H1a + H2b + H-8 + H-16b + H-11b + H-7b), 1.40-1.34 (m, 4H, H-22a,b + H-23a,b), 1.26 (m, 1H, H-15b), 1.07 (m, 1H, H12b), 1.01 (s, 3H, C19H_3), 0.98-0.88 (m, 3H, H-14 + H-1b + H-9), 0.77 (s, 3H, C18H_3); ^{13}C NMR (100 MHz, $CDCl_3$) δ 141.0, 121.5, 89.2, 71.8, 70.1, 51.8, 51.6, 50.4, 43.0, 42.4, 38.1, 37.5, 36.7, 31.9, 31.8, 31.7, 30.7, 29.9, 29.0, 28.3, 26.7, 26.0, 23.6, 20.9, 19.6, 11.7; HRMS (m/z): [M+H]· calcd. for $C_{25}H_{42}N_3O_2^+$, 416.3272; found, 416.3265

17β-(9'-azidononanoxyl)-5-androsten-3β-ol (3)

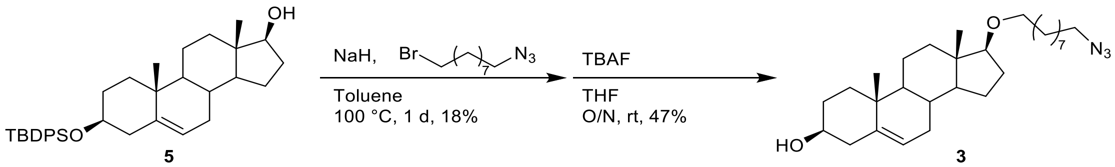

5 **3**

The preparation of **3** took two steps starting from **5** (8% total yield), which is similar to that of **1**. TLC of **3** (ethyl acetate/hexanes = 15/85, v/v): R_f= 0.20; $[\alpha]_D^{25}$ = –85.5 (c = 0.046, $CHCl_3$); ^{1}H NMR (500 MHz, $CDCl_3$) δ 5.35 (d, J = 5.2 Hz, 1H, H-6), 3.52 (m, 1H, H-3), 3.48-3.38 (m, 2H, H-20a,b), 3.28 (t, J = 8.4 Hz, 1H, H-17), 3.26 (t, J = 7.3 Hz, 2H, H-28a,b), 2.32-2.20 (m, 2H, H-4a + H-4b), 2.04-1.96 (m, 2H, H-16a +H-7a), 1.94-1.90 (m, 1H, H-12a), 1.87-1.80 (m, 3H, H-11a + H-15a + H-2a), 1.64-1.43 (m, 10H, H-21a,b + H-27a,b + H1a + H2b + H-8 + H-16b + H-11b + H-7b), 1.38-1.22 (m, 11H, H-22a,b + H-23a,b + H-24a,b + H-25a,b + H-26a,b + H15b), 1.08 (m, 1H, H12b), 1.02 (s, 3H, C19H_3), 0.99-0.87 (m, 3H, H-14 + H-1b + H-9), 0.77 (s, 3H, C18H_3); ^{13}C NMR (100 MHz, $CDCl_3$) δ 141.1, 121.7, 89.3, 72.0, 70.4, 51.9, 51.7, 50.5, 43.0, 42.5, 38.2, 37.5, 36.8, 32.0, 32.0, 31.7, 30.4, 29.6, 29.6, 29.3, 29.1, 28.4, 26.9, 26.4, 23.6, 21.0, 19.6, 11.7; HRMS (m/z): [M+H]· calcd. for $C_{28}H_{48}N_3O_2^+$, 458.3741; found, 458.3730

Supplementary scheme 2

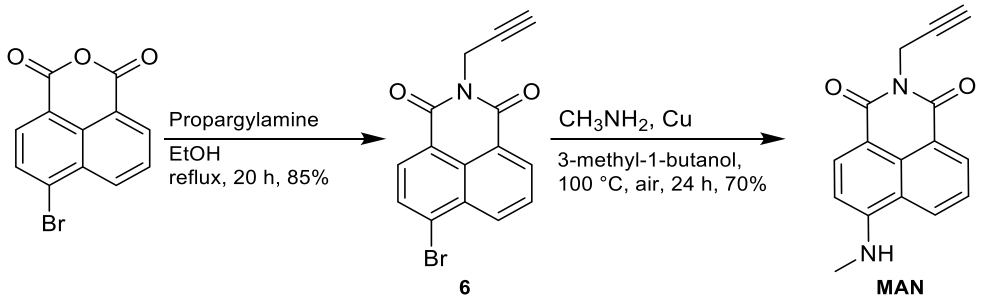

4-*N*-methylamino-1,8-napthalimidopropyne (MAN)

To a solution of 4-bromo-1,8-naphthalic anhydride (1.0 g, 3.61 mmol) in anhydrous ethanol (200 mL) was added propargylamine (0.24 g, 4.33 mmol) at room temperature. The reaction mixture was heated under a reflux condition for 20 h and then cooled down to room temperature. After filtration to remove insoluble precipitates, the filtrate was concentrated *in vacuo* to afford the crude that was purified by flash column chromatography with ethyl acetate/hexanes (in a gradient elution) to afford compound **6** as a white solid. The intermediate **6** (0.96 g, 3.07 mmol) and a catalytic amount of copper power (9.8 mg, 0.15 mmol) was added to the mixture of 30% aqueous methylamine solution (1.66 mL, 15.35 mmol) and 3-methyl-1-butanol (10 mL) in a 30 mL screwed tube at room temperature. The resulting mixture was immersed into the oil bath heated at 100 °C. After the reaction was completed, the reaction mixture was cooled down to room temperature and most of the solvent was removed *in vacuo*. The crude was diluted with ethyl acetate (20 mL) and the resulting mixture was washed with water and brine sequentially, dried over $MgSO_4$, filtrated and concentrated *in vacuo* to afford the dried residue that was purified by flash column chromatography with petroleum ether/ethyl acetate (in a gradient elution) to give the desired compound MAN (0.57 g, 60% in two steps). TLC (ethyl acetate/hexanes = 25/75, v/v): R_f= 0.30; $[\alpha]_D^{25}$ = 5.0 (*c* = 0.053, DMF); ^{1}H NMR (500 MHz, $CDCl_3$) δ 8.63 (d, *J* = 7.3 Hz, 1H, Ar-*H*), 8.54 (d, *J* = 8.3 Hz, 1H, Ar-*H*), 8.09 (dd, *J* = 8.3 Hz, 1H, Ar-*H*), 7.64 (t, *J* = 8.3 Hz, 1H, Ar-*H*), 6.73 (d, *J* = 8.3 Hz, 1H, Ar-*H*), 5.42 (m, 1H, N*H*-CH_3), 4.95 (m, 2H, N-CH_2-CCH), 3.16 (d, *J* = 5.2 Hz, 3H, NH-CH_3), 2.17 (m, 1H, N-CH_2-CC*H*); ^{13}C NMR (100 MHz, $CDCl_3$) δ 164.7, 164.1, 151.7, 135.3, 131.5, 130.0, 127.7, 124.5, 122.1, 120.4, 108.3, 103.7, 78.0, 70.3, 35.2, 30.0. HRMS (*m/z*): [M+H]· calcd. for $C_{16}H_{13}N_2O_2^+$, 265.0972; found, 265.0965

Supplementary spectra data 1

Assignment of ^1H and ^{13}C NMR spectra of fluorophore-labeled CPG

CPG(14:0/19c:0)

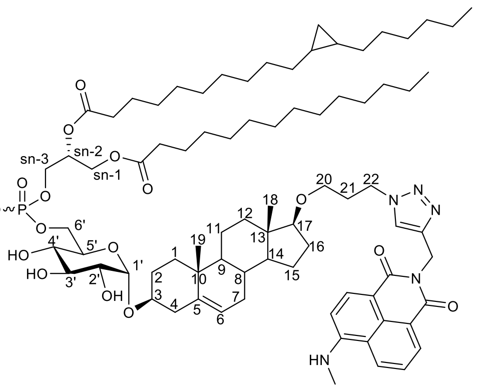

^{1}H NMR (600 MHz, $CDCl_3/CD_3OD$) δ 8.40 (d, *J* = 7.3 Hz, 1H, Ar-*H*), 8.30 (d, *J* = 8.5 Hz, 1H, Ar-*H*), 8.16 (d, *J* = 8.3 Hz, 1H, Ar-*H*), 7.50 (s, 1H, *H*-triazole), 7.42 (t, *J* = 7.7 Hz, 1H, Ar-*H*), 6.50 (d, *J* = 8.6 Hz, 1H, Ar-*H*), 5.38 (d, *J* = 14.8 Hz, 1H, CCH_2N), 5.31 (d, *J* = 14.8 Hz, 1H, CCH_2N), 5.19 (s, 1H, *H*-6), 5.12 (m, 1H, *sn*-2-C*H*), 4.88 (d, *J* = 4.1 Hz, 1H, *H*-1'), 4.30 (dd, *J* = 6.6 Hz, 7.1 Hz, 2H, H-22a,b), 4.27 (dd, *J* = 2.5 Hz, 11.8 Hz, 1H, *sn*-1-CH_{2a} glycerol), 4.18 (t, *J* = 11.0 Hz, 1H, *H*-6'a), 4.04 (dd, *J* = 6.6 Hz, 11.8 Hz, 1H, *sn*-1-CH_{2b} glycerol), 3.86 (m, 2H, *sn*-3-CH_{2a} glycerol + *sn*-3-CH_{2b} glycerol), 3.80 (t, *J* = 11.0 Hz, 1H, *H*-6'b), 3.61 (dd, *J*= 9.1, 9.7 Hz, 1H, *H*-3'), 3.55 (m, 1H, *H*-5'), 3.48 (dd, *J*= 9.7, 9.4 Hz, 1H, *H*-4'), 3.37 (dd, *J*= 4.1, 9.1 Hz, 1H, *H*-2'), 3.33 (m, 1H, *H*-3), 3.18 (m, 1H, H-20a), 3.13 (m, 1H, H-20b), 3.00-2.90 (m, 3H, *H*-17 + $NHCH_3$), 2.21-2.17 (m, 5H, *H*-4, $COCH_2C_{12}H_{25}$, $COCH_2C_{17}H_{33}$), 1.97-1.93 (m, 2H, H-21a,b), 1.84-0.55 (m, 74H, $COCH_2C_{12}H_{25}$ + $COCH_2C_8H_{16}$-C_3H_4-C_6H_{13} + *H*-1a,b, *H*-2a,b, *H*-7a,b, *H*-8, *H*-9, *H*-11a,b, *H*-12a,b, *H*-14, *H*-15a,b, *H*-16a,b, CH_3-18), 0.54-0.50 (m, 5H, cyclopropyl 2×C*H* + CH_3-19), 0.43 (ddd, *J* = 8.3, 8.3, 4.1 Hz, 1H, cyclopropyl CH_{2a}), -0.46 (ddd, *J* = 5.1, 5.1, 4.1 Hz, 1H, cyclopropyl CH_{2b}); **^{13}C NMR** (150 MHz, $CDCl_3$) δ 174.0 ($COCH_2C_{12}H_{25}$), 173.6 ($COCH_2C_{17}H_{33}$), 164.7, 164.1, 151.7, 144.0, 140.4 (*C*-5), 135.3, 131.5, 130.0, 127.7, 124.5, 124.0, 122.1, 121.8 (*C*-6), 120.4, 108.3, 103.7, 97.2 (*C*-1'), 89.2 (*C*-17), 77.9 (*C*-3), 72.8 (*C*-3'), 71.9 (*C*-2'), 71.2 (*C*-5'), 70.2 (*sn*-2-CH_2), 68.6 (*C*-4'), 65.3, 64.1 (*C*-6'), 63.6 (*sn*-3-CH_2), 62.6 (*sn*-1-CH_2), 51.1, 49.8, 47.2, 42.6, 40.0, 37.5, 36.9, 36.6, 35.2, 34.3, 34.1, 33.6, 32.0, 32.0, 31.5, 31.3, 30.3, 30.2, 30.0, 29.7, 29.7, 29.7, 29.7, 29.7, 29.7, 29.6, 29.6, 29.6, 29.6, 29.4, 29.4, 29.4, 29.4, 29.2, 29.2, 28.7, 28.7, 28.7, 24.9, 24.9, 23.2, 22.7, 19.3, 15.7 (Cyclopropyl *C*H), 15.7 (Cyclopropyl *C*H), 14.2, 14.2, 11.4, 10.9 (Cyclopropyl CH_2); **^{31}P NMR** (200 MHz, $C_3D_8O/D_2O/H_2O$ 1/0.83/0.67) δ -0.0073.

Supplementary spectra data 2

Assignment of ^{1}H and ^{13}C NMR spectra of fluorophore-labeled CAG

CAG(14:0)

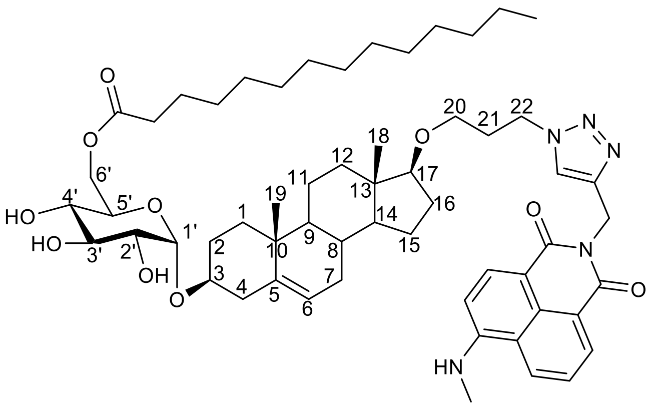

^{1}**H NMR** (500 MHz, $CDCl_3$) δ 8.46 (d, *J* = 7.1 Hz, 1H, Ar-*H*), 8.42 (d, *J* = 8.4 Hz, 1H, Ar-*H*), 7.97 (d, *J* = 8.1 Hz, 1H, Ar-*H*), 7.60 (s, 1H, *H*-triazole), 7.44 (t, *J* = 7.5 Hz, 1H, Ar-*H*), 6.62 (d, *J* = 8.4 Hz, 1H, Ar-*H*), 5.75 (q, *J* = 6.6 Hz, 4.2 Hz, 1H, N*H*CH_3), 5.49 (d, *J* = 14.5 Hz, 1H, CCH_2N), 5.45 (d, *J* = 14.5 Hz, 1H, CCH_2N), 5.32 (d, *J* = 5.0 Hz, 1H, *H*-6), 5.01 (d, *J* = 4.2 Hz, 1H, *H*-1'), 4.51 (dd, *J* = 12.3 Hz, 4.7 Hz, 1H, *H*-22a), 4.41-4.38 (m, *J* = 6.8 Hz, 2H, *H*-6a' + *H*-6'b), 4.22 (dd, *J* = 12.3 Hz, 2.6 Hz, 1H, *H*-22b), 3.84 (ddd, *J*= 9.8, 6.8, 2.1 Hz, 1H, *H*-5'), 3.72-3.69 (m, *J*= 10.3, 9.3 Hz, 1H, *H*-3'), 3.50-3.44 (m, *J*= 4.2, 10.3 Hz, 2H, *H*-2' + *H*-3), 3.38-3.28 (m, *J*= 9.3, 9.8 Hz, 3H, *H*-4' + *H*-20a,b), 3.16 (t, *J* = 8.3 Hz, 1H, *H*-17), 3.09 (m, 3H, NHCH_3), 2.36-2.33 (m, 3H, *H*-4, CO$CH_2C_{12}H_{25}$), 2.09-2.04 (m, 2H, H-21a,b), 1.84-0.55 (m, 45H, CO$CH_2C_{12}H_{25}$ + *H*-1a,b, *H*-2a,b, *H*-7a,b, *H*-8, *H*-9, *H*-11a,b, *H*-12a,b, *H*-14, *H*-15a,b, *H*-16a,b, CH_3-18), 0.68 (s, 3H, CH_3-19); ^{13}**C NMR** (150 MHz, $CDCl_3$) δ 174.7 ($COCH_2C_{12}H_{25}$), 164.7, 164.1, 151.4, 144.1, 140.6 (*C*-5), 135.1, 131.3, 129.7, 127.2, 124.5, 123.9, 122.2, 121.9 (*C*-6), 120.1, 108.8, 103.8, 97.2 (*C*-1'), 89.3 (*C*-17), 78.4 (*C*-3), 74.1 (*C*-3'), 71.9 (*C*-2'), 70.2 (*C*-4'), 70.0 (*C*-5'), 65.7, 63.6 (*C*-6'), 51.3, 50.1, 47.5, 42.8, 40.2, 37.7, 37.1, 36.7, 35.1, 34.4, 33.5, 32.1, 31.6, 31.5, 30.6, 30.3, 30.2, 29.9, 29.8, 29.8, 29.8, 29.5, 29.5, 29.5, 29.4, 29.4, 25.0, 22.9, 20.6, 19.4, 14.3, 11.6.

Supplementary spectra data 3

Determination of the structures of cholesteryl glucoside derivatives by a series of NMR experiments. CPG(14:0/19c:0) was used as a representative example to explain how we determined the structures of cholesteryl glucoside derivatives by a series of NMR experiments. The data and the corresponding assignments were in consistence with those reported by Gervay-Hague and coworkers [1].

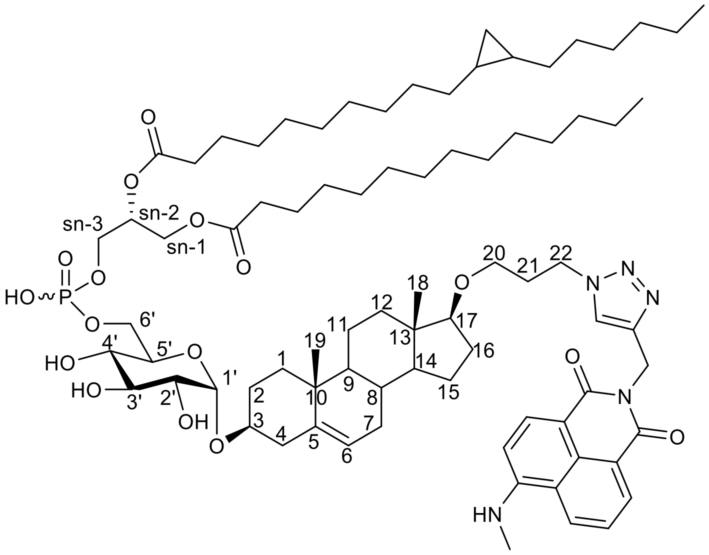

CPG(14:0/19c:0)

A. To identify six sets of protons/carbons by ^{13}C NMR, DEPT and HSQC

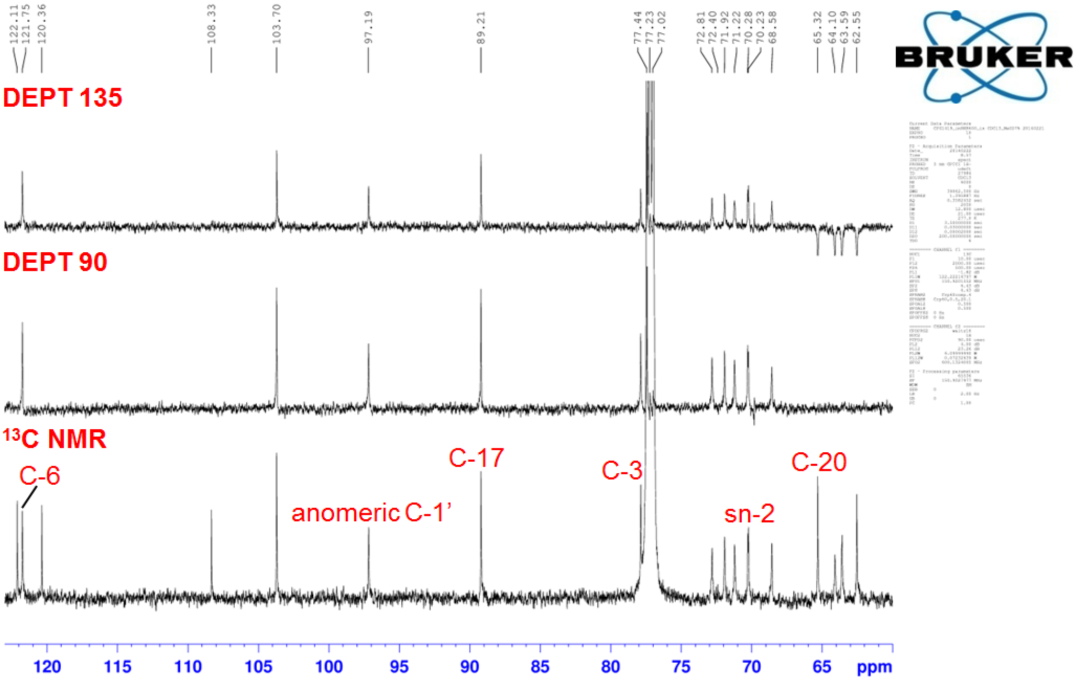

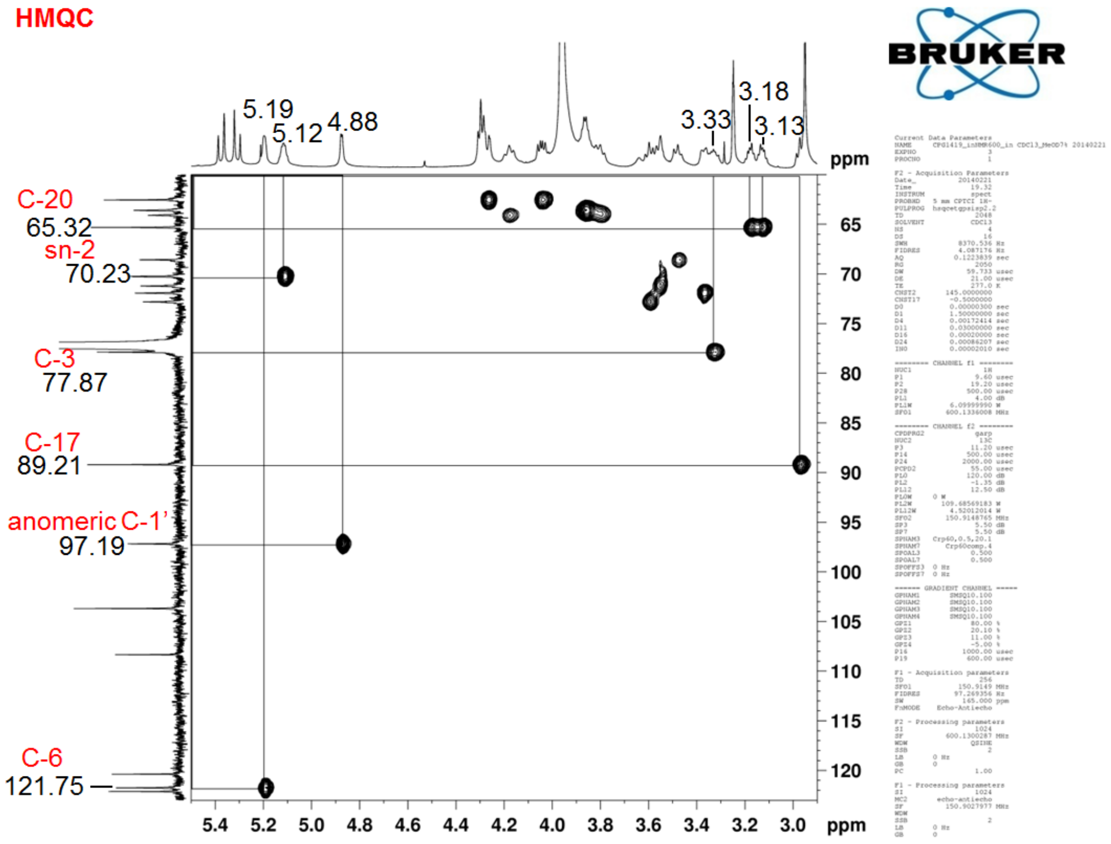

B. To correlate the H-1'/C-1' with the C-3/H-3. To correlate the H-17/C-17 with the indicated carbon/protons of linker.

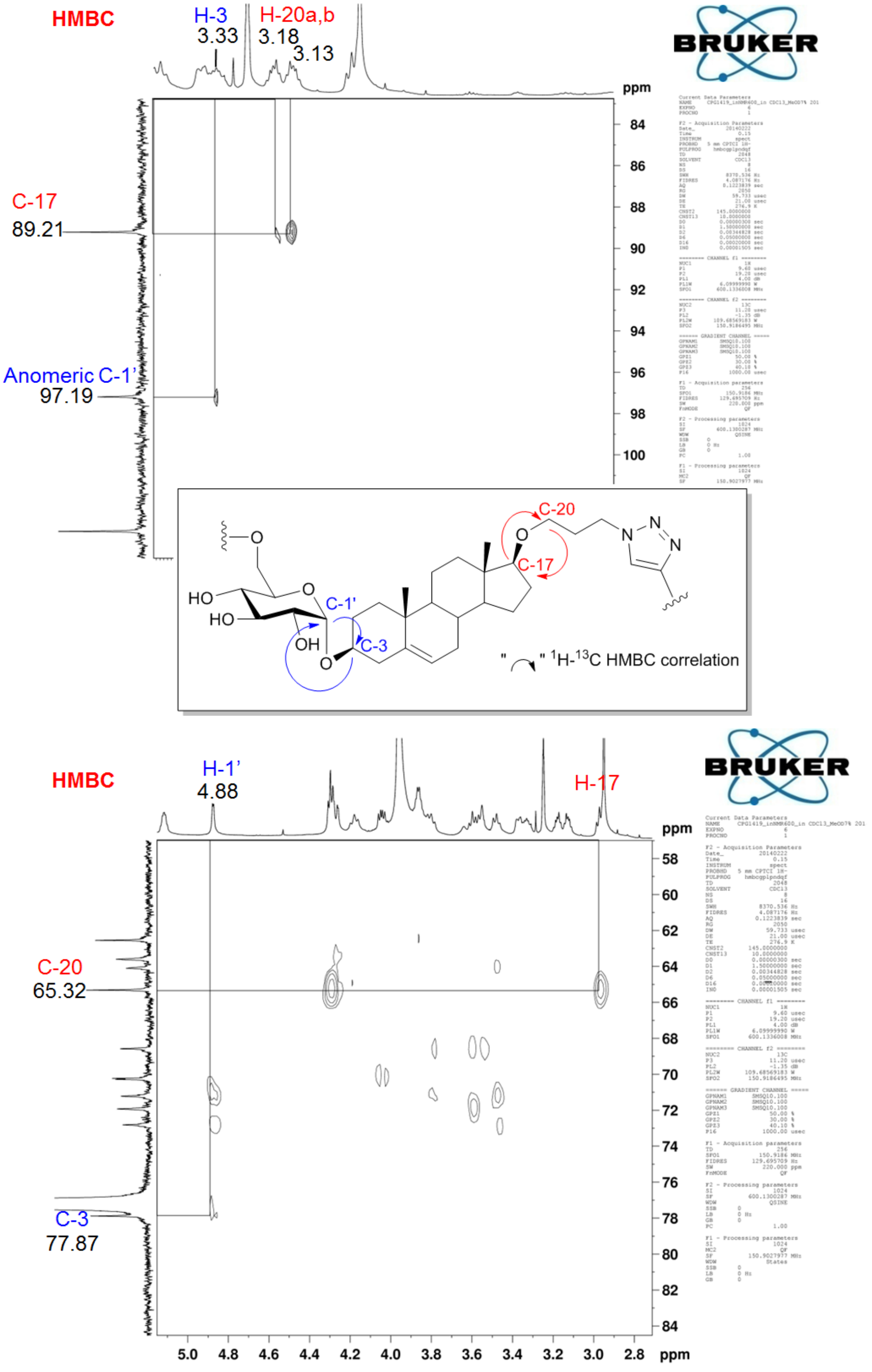

C. To identify the protons/carbons of glucose moiety and those of the linker by 2D-COSY

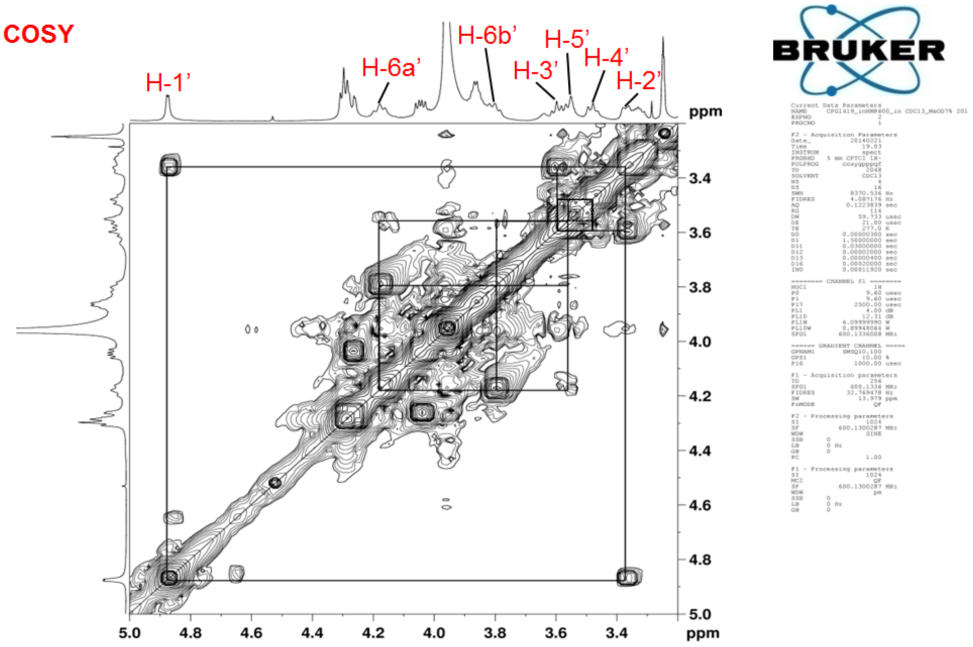

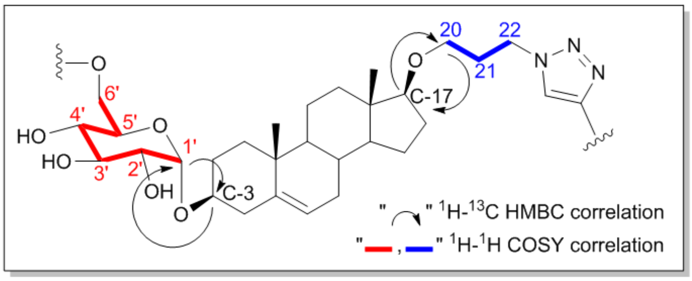

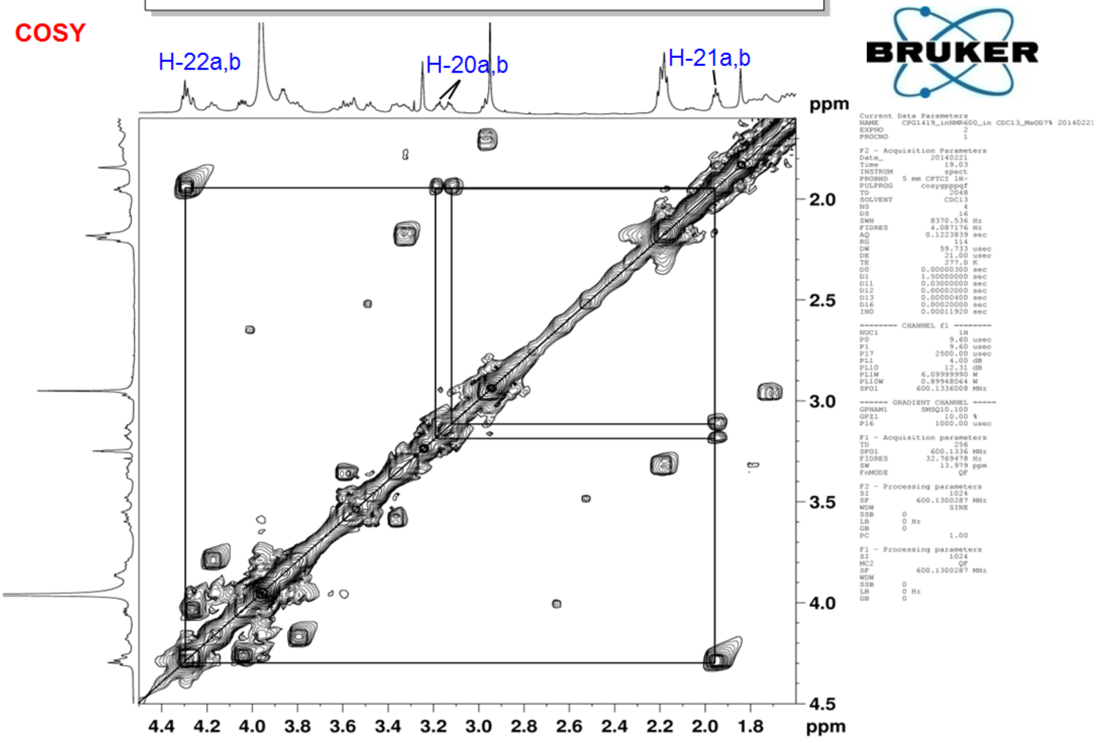

D. To identify the protons of glycerol by HMBC and COSY.

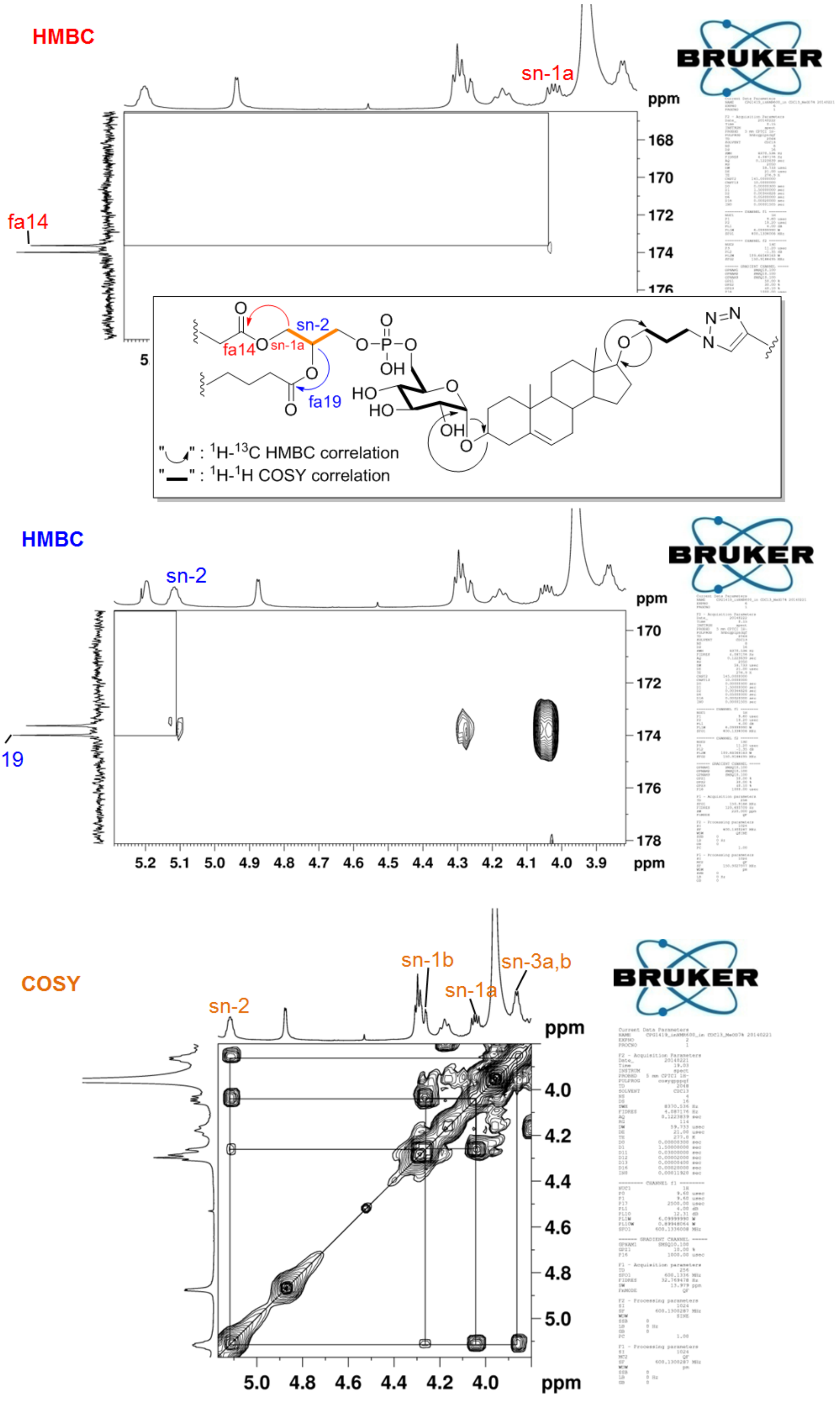

Supplementary methods

H. pylori strains and bacterial culture

The standard *H. pylori* strain 26695 (ATCC 700392) was obtained from the Bioresource Collection and Research Center, Hsinchu, Taiwan. Clinically isolated strains were obtained during endoscopic examination at National Taiwan University Hospital, Taiwan. The ΔCGT strain was constructed by insertion of the chloramphenicol resistance cassette into the cholesterol glucosyltransferase gene (*hp0421*) via homologous recombination.[2] *H. pylori* strains were cultured on Brucella agar plates (Becton Dickinson) supplemented with 5% sheep blood for 2–4 days at 37 °C under microaerophilic conditions using the Anaeropack Campylo system (Mitsubishi Gas Chemical, Tokyo, Japan).

Coupling assay for cholesterol glucosyltransferase activity

The reaction activity of cholesterol glucosyltransferase was measured by coupling with the reactions of pyruvate kinase and lactate dehydrogenase to monitor the consumption of NADH with absorption at 340 nm.[3] The activity was measured at 37 °C in a final volume of 0.1 mL containing 100 mM HEPES buffer (pH 7.5), 2.5 mM $MgCl_2$, 0.5 mM phosphoenolpyruvate (PEP), 0.5 mM NADH, 0.1% triton X-100, 2.5 U lactate dehydrogenase, 2U pyruvate kinase. Different fixed amounts of cholesterol were added. The assays were initiated upon the addition of 2 mM UDP glucose.[4]

Quantitation of phospholipids

The quantification was performed according to the improved procedures using malachite green.[5] The extracted lipids were separated by two-dimensional thin-layer chromatography (2D-TLC) with chloroform/methanol/water (7/1.6/0.2, v/v/v) as the first solvent eluent and chloroform/methanol/water (7/3/0.5, v/v/v) as the second one. Scraped spots obtained from 2D-TLC were transferred into clean glass tubes and added 0.2 mL water and 0.4 mL perchloric acid. The resulting mixtures were heated at 180 °C with sealed tubes for 30-60 min until the yellow color disappeared. After being cooled down to room temperature, the residue was added 0.2 mL water and 2 mL of working solution that contained malachite green and ammonium molybdate. The working solution was prepared according to literature [5]. The resulting mixture was stirred for 20 min and then measured for the absorption at 660 nm with a UV-Vis spectrophotometer.

Phospholipase hydrolysis

Enzymatic hydrolysis was performed as previously described with some modifications.[6,7] In the PLA1 assay, 5.7 mg MAN-labeled CPG(14:0/19c:0) dissolved in D_2O/d_9-isopropanol (2:1) was treated with 0.426% (v/v) PLA1 (*Thermomyces lanuginosus*, Sigma) in 10 mM Bis-Tris buffer, pH 6.0, containing 50 mM $CaCl_2$ for 10 min at 37 °C. In the PLA2 assay, 10 μg MAN-labeled CPG(14:0/19c:0) dissolved in D_2O/d_9-isopropanol (9:1) was treated with 200 U/mL PLA2 (honey bee venom, Sigma) in 100 mM Tris-HCl, pH 8.5, for 18 h at 37 °C. The reaction progress was monitored by HPLC. After reaction

completion, the reaction mixtures were heated for 5 min at 100 °C and analyzed by ^{31}P NMR and UPLC-MS.

^{31}P NMR spectroscopic measurements

^{31}P NMR spectroscopic measurements were performed as previously described.[8] Sample preparation of MAN-labeled lyso-CPG(19c:0) was described in the procedure of PLA1 hydrolysis. ^{31}P NMR spectra were recorded in 5 mm NMR tubes on a Bruker AVII 500 LC-NMR spectrometer operating at 202.46 MHz for ^{31}P. All measurements were performed at 25 °C and Waltz-16 composite pulse decoupling to eliminate ^{31}P-^{1}H coupling. Other NMR parameters were shown as follows: 90 ° pulse (9.9 μsec), pulse delay (2 sec) and line broadening (1 Hz). Spectra were processed using the software topspin version 3.0 (Bruker, Germany).

Live-cell imaging of AGS cells (to observe lipid rafts formation) that were pretreated with CAGs.

AGS cells were seeded onto 35 mm dish at 37 °C with 10% CO_2 for overnight. Cells were first washed with PBS for three times before being treated with 5 mM MβCD in serum-free medium for 1 h. After further PBS washes, cells were incubated with Alexa Fluor 594-conjugated cholera toxin subunit beta (Molecular Probes, USA) for 10 min, washed with PBS for three times, and then treated with 10 μM CAG (CAG(10:0, 14:0 or 18:0)) at 37 °C for 0-30 min. The images of serial optical sections (1024 × 1024 pixels) were obtained under 100× Plan-NEOFLUAR lens (laser 543 nm) at 5 min intervals using Zeiss LSM 510 confocal microscope. The live-cell images shown were representative of three independent experiments.

Supplementary figures

A

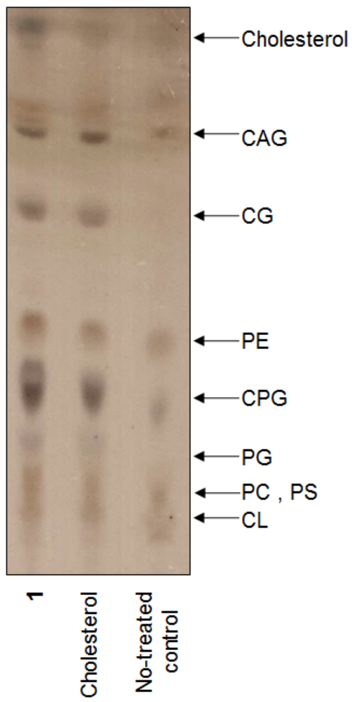

B

Phospholipid compositions in *H. pylori*

Treatment / Product	Cholesterol	**1**
PE	49.1 ± 0.3	47.0 ± 2.6
CL	42.9 ± 0.1	44.2 ± 2.4
PG	0.40 ± 0.07	0.43 ± 0.19
PS	0.52 ± 0.10	0.57 ± 0.07
PC	0.03 ± 0.01	0.05 ± 0.02
CPG	7.04 ± 0.17	7.77 ± 0.17

Fig. S1. Lipid compositions in *H. pylori* of which the bacterial culture was supplemented with cholesterol or compound **1**. (A) Analysis was carried out by thin layer chromatography (TLC). Total lipids were extracted by Folch extraction from *H. pylori* pre-treated with cholesterol or **1** for two days. The solvent used for TLC was a mixture of chloroform/methanol/water (70/16/2) in volume. The TLC was stained with α-naphthol for identification of glycolipids. (B) Comparison of phospholipid compositions obtained from the *H. pylori* culture that was supplemented with cholesterol or **1**. The compositions and relative levels of phospholipids were determined by the absorption (λ_{abs} = 660 nm) from the molybdate complexes. Phospholipids were separated by 2D thin-layer chromatography and digested with perchloric acid to obtain phosphate ion. Phosphate ion was then complexed with malachite green and ammonium molybdate to form the chromogenic molybdate complex. Values were the means of three determinations ± standard deviations. Abbreviations: PE, phosphatidylethanolamine; PG, phosphatidylglycerol; PC, phosphatidylcholine; PS, phosphatidylserine; CL, cardiolipin.

A

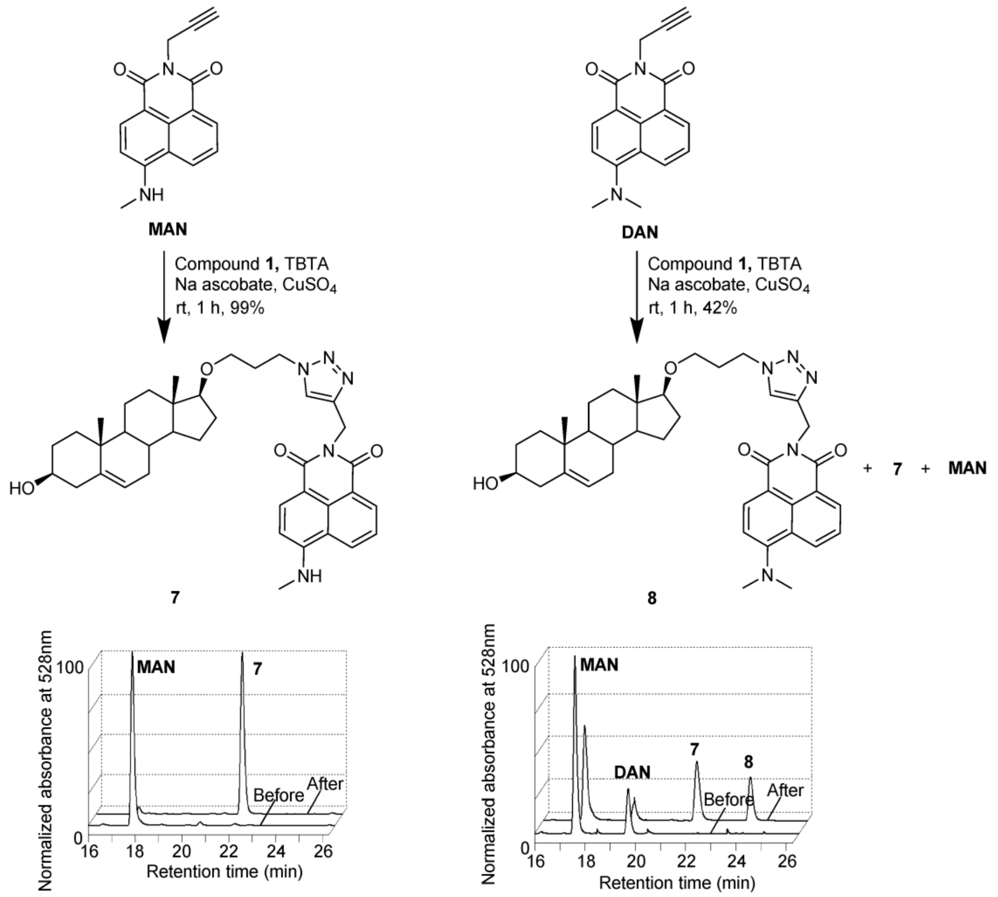

B

Fluorophore	Structure	Results
Alkyne cyanine dye 718 (Sigma)	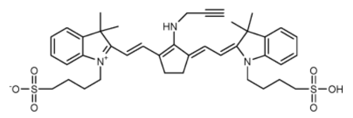	Unstable
Alkyne MegaStokes dye 608 (Sigma)	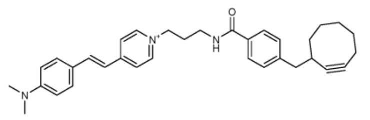	Unstable, low yield in click reaction
Acetylene Fluor 545 (Jena Bioscience)	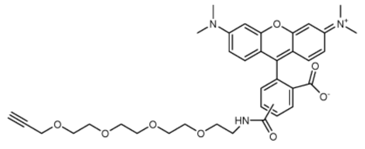	Unstable
Chromeo 642-Alkyne (Jena Bioscience)	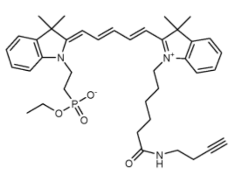	Stable, but low yield in click reaction

Fig. S2. The molecular structures of different fluorescent dyes and their results of the Cu[I]-catalyzed azide/alkyne cycloaddition. (A) MAN and DAN were reacted with **1** via Cu(I)-catalyzed 1,3-dipolar cycloaddition in the presence of tris(benzyltriazolylmethyl)amine (TBTA), sodium ascorbate and $CuSO_4$ for 1 h at room temperature. The HPLC spectra show the product analysis before and after the click reactions. Upon the HPLC analysis, compound DAN was self-degraded to MAN before performing the click reaction. (B) The molecular structures of four different purchased fluorescent dyes and their results of the Cu[I]-catalyzed azide/alkyne cycloaddition. These reaction progresses were monitored by TLC or HPLC.

Fig. S3. The molecular structures and corresponding MS and MS/MS spectra of (A) CAG(14:0); (B) CAG(18:1); (C) CAG(19c:0); (D) CAG(16:0); (E) CAG(18:0); (F) CAG(18:2); (G) CAG(20:4); (H) CPG(14:0/14:0); (I) CPG(14:0/18:1); (J) CPG(19c:0/18:1); (K) CPG(19c:0/19c:0); (L) CPG(14:0/16:0); (M) CPG(16:0/18:1); (N) CPG(16:0/19c:0); (O) CPG(18:1/18:1); (P) CPG(18:0/18:0); (Q) lyso-CPG(14:0); (R) lyso-CPG(18:1); (S) lyso-CPG(19c:0). All the parent ions and their daughter ions were detected by MS and MS/MS, respectively. In the molecular structures shown below, the dashed arrows and numbers indicate the fragmented structures and the corresponding molecular weights, respectively. CID, collision-induced dissociation; HCD, higher-energy collisional dissociation.

A

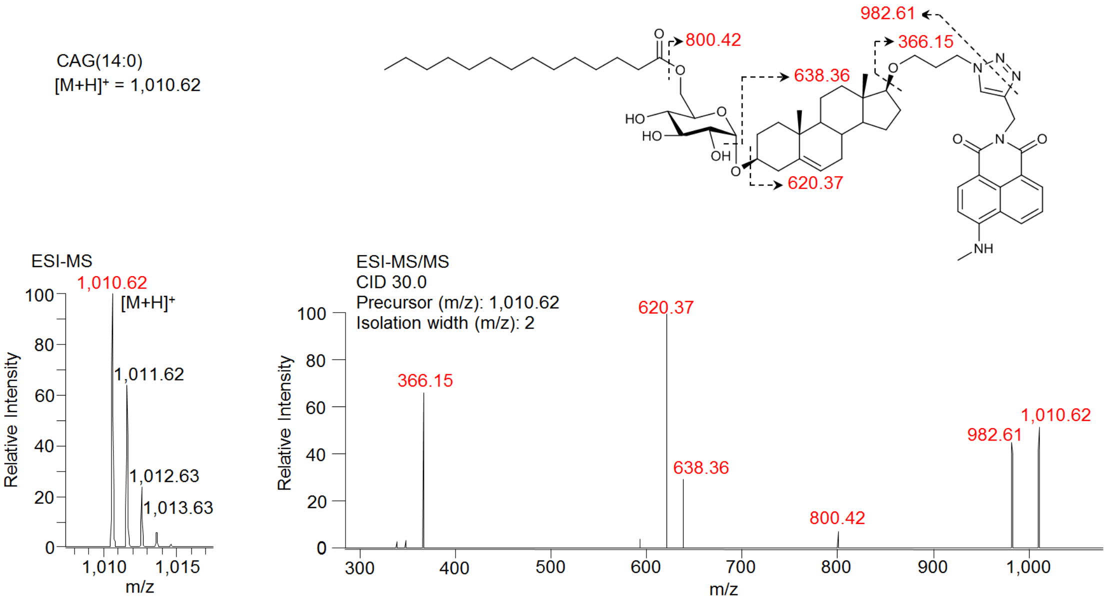

B

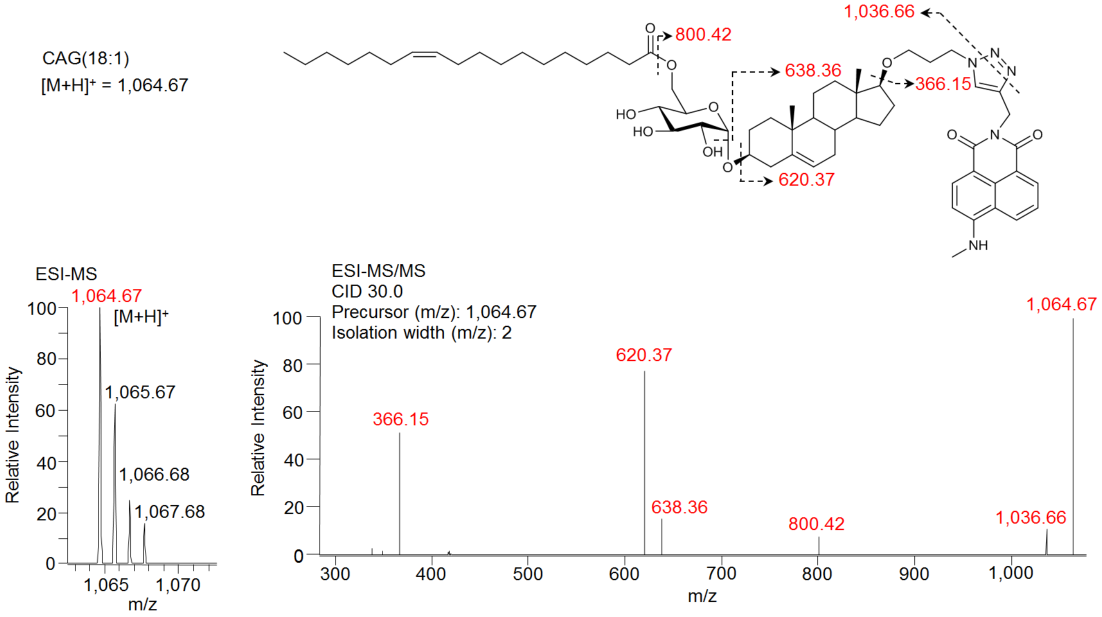
CAG(18:1)
[M+H]⁺ = 1,064.67
800.42
1,036.66
638.36
366.15
620.37
HO
HO
OH
O
N
NH
ESI-MS
1,064.67
[M+H]⁺
1,065.67
1,066.68
1,067.68
Relative Intensity
m/z
1,065
1,070
ESI-MS/MS
CID 30.0
Precursor (m/z): 1,064.67
Isolation width (m/z): 2
1,064.67
620.37
366.15
638.36
800.42
1,036.66
300
400
500
600
700
800
900
1,000

C

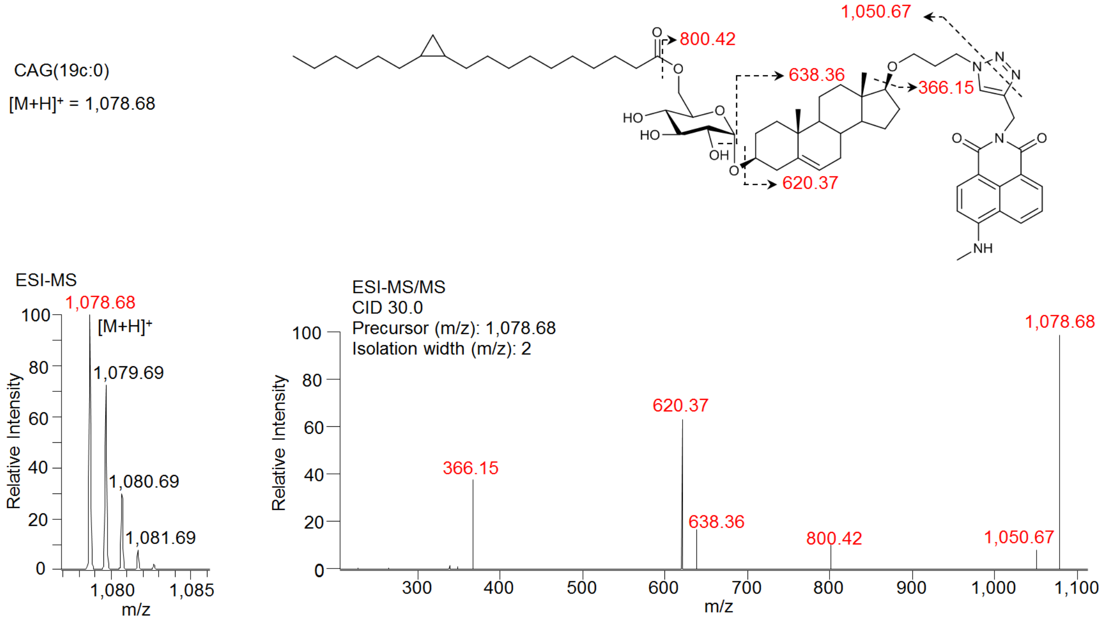
CAG(19c:0)
[M+H]⁺ = 1,078.68
800.42
1,050.67
638.36
366.15
620.37
HO
HO
OH
O
N
NH
ESI-MS
1,078.68
[M+H]⁺
1,079.69
1,080.69
1,081.69
Relative Intensity
m/z
1,080
1,085
ESI-MS/MS
CID 30.0
Precursor (m/z): 1,078.68
Isolation width (m/z): 2
1,078.68
620.37
366.15
638.36
800.42
1,050.67
300
400
500
600
700
800
900
1,000
1,100

D

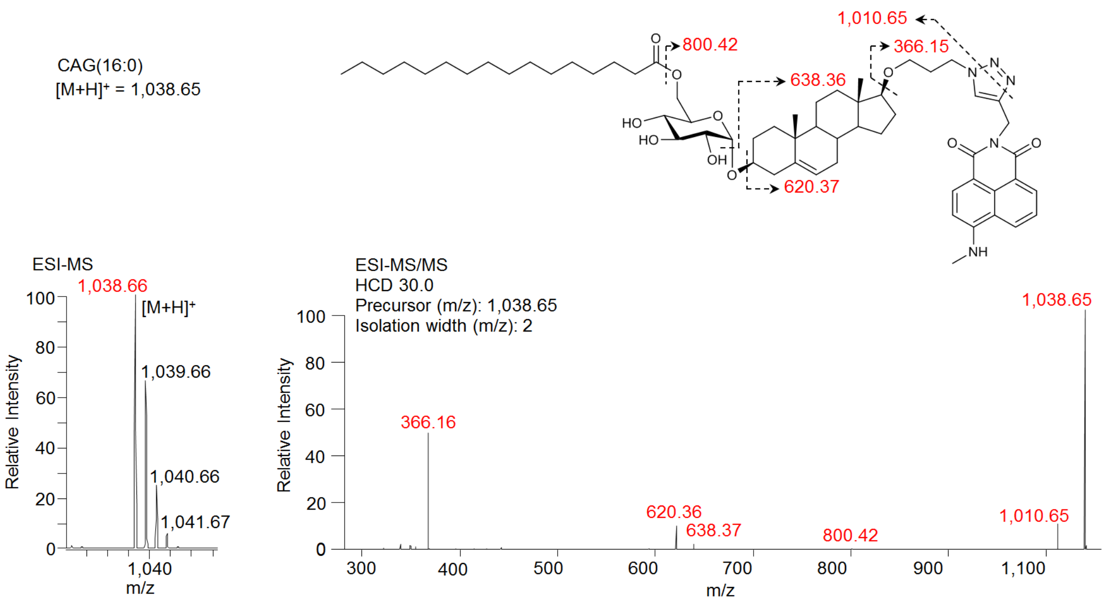
CAG(16:0)
[M+H]⁺ = 1,038.65
800.42
1,010.65
366.15
638.36
620.37
ESI-MS
1,038.66
[M+H]⁺
1,039.66
1,040.66
1,041.67
Relative Intensity
m/z
ESI-MS/MS
HCD 30.0
Precursor (m/z): 1,038.65
Isolation width (m/z): 2
1,038.65
366.16
620.36
638.37
800.42
1,010.65

E

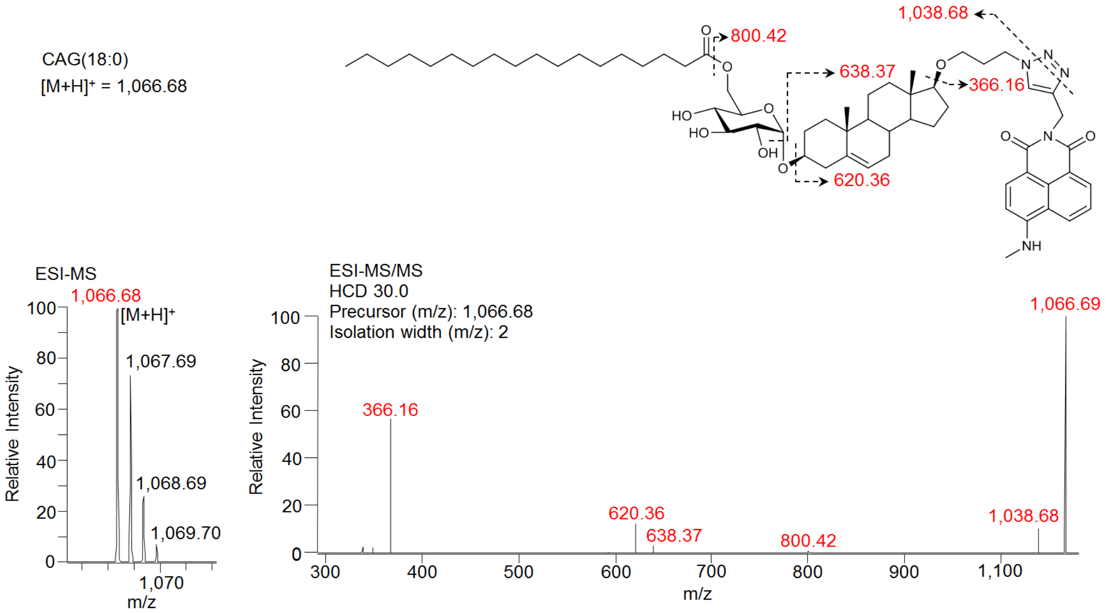
CAG(18:0)
[M+H]⁺ = 1,066.68
800.42
1,038.68
638.37
366.16
620.36
ESI-MS
1,066.68
[M+H]⁺
1,067.69
1,068.69
1,069.70
Relative Intensity
m/z
ESI-MS/MS
HCD 30.0
Precursor (m/z): 1,066.68
Isolation width (m/z): 2
1,066.69
366.16
620.36
638.37
800.42
1,038.68

F

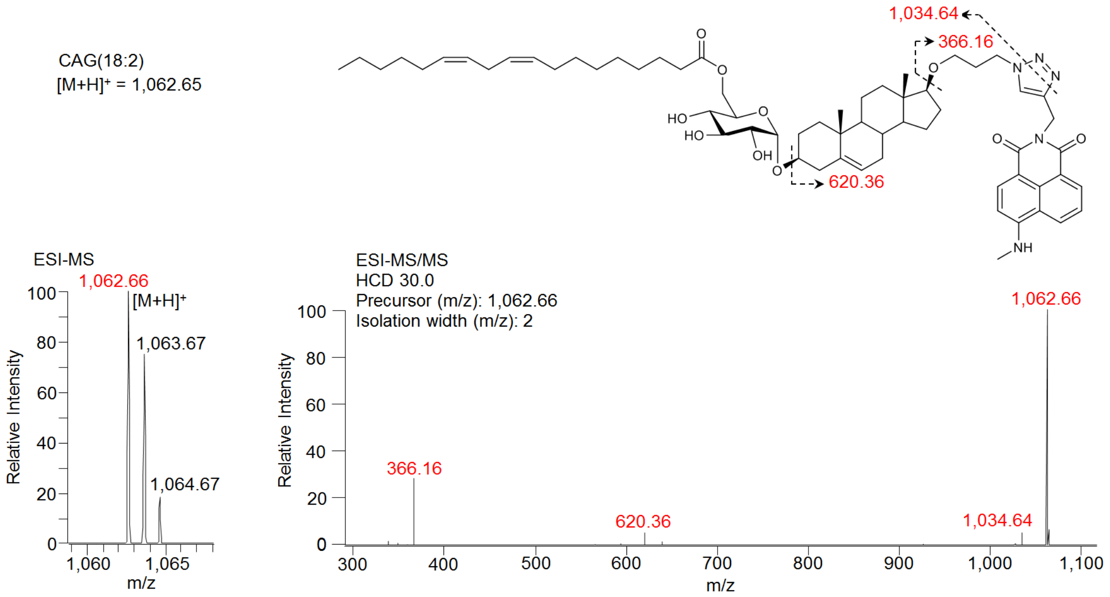
CAG(18:2)
[M+H]+ = 1,062.65
1,034.64
366.16
620.36
HO
HO
OH
O
N
NH
ESI-MS
1,062.66
[M+H]+
1,063.67
1,064.67
Relative Intensity
m/z
ESI-MS/MS
HCD 30.0
Precursor (m/z): 1,062.66
Isolation width (m/z): 2
1,062.66
366.16
620.36
1,034.64
Relative Intensity
m/z

G

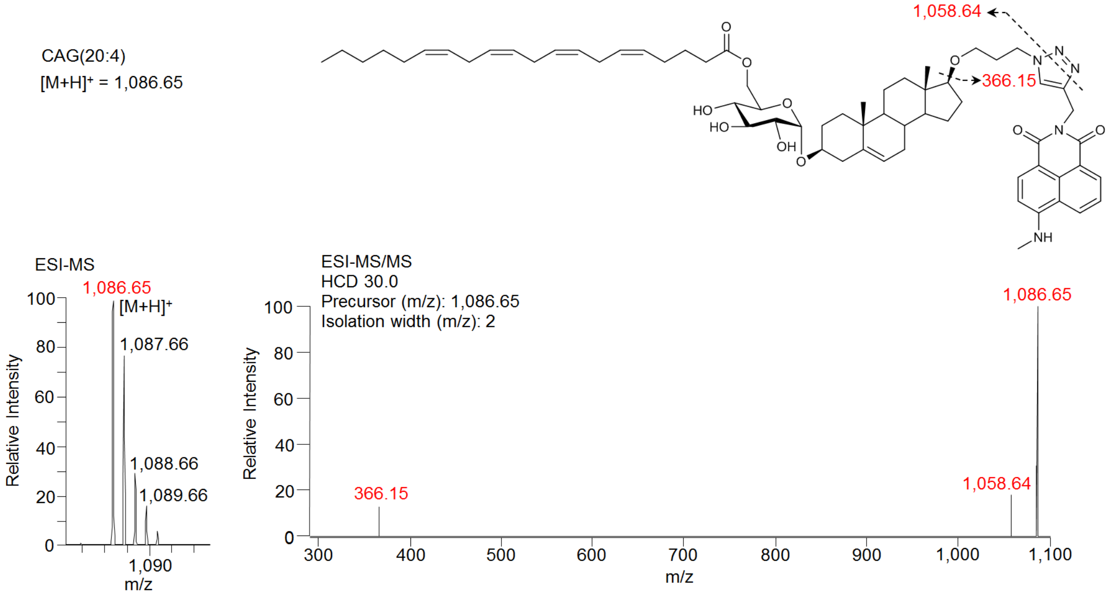
CAG(20:4)
[M+H]+ = 1,086.65
1,058.64
366.15
HO
HO
OH
O
N
NH
ESI-MS
1,086.65
[M+H]+
1,087.66
1,088.66
1,089.66
Relative Intensity
m/z
ESI-MS/MS
HCD 30.0
Precursor (m/z): 1,086.65
Isolation width (m/z): 2
1,086.65
1,058.64
366.15
Relative Intensity
m/z

H

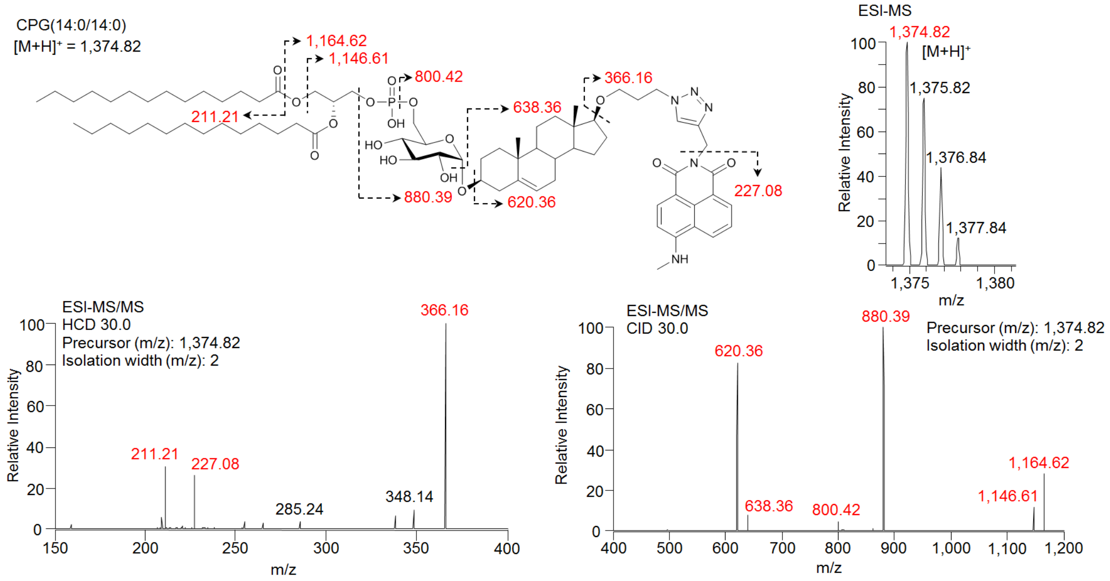

CPG(14:0/14:0)
[M+H]+ = 1,374.82
1,164.62
1,146.61
800.42
366.16
638.36
211.21
880.39
620.36
227.08
ESI-MS
1,374.82
[M+H]+
1,375.82
1,376.84
1,377.84
Relative Intensity
m/z
ESI-MS/MS
HCD 30.0
Precursor (m/z): 1,374.82
Isolation width (m/z): 2
366.16
211.21
227.08
285.24
348.14
ESI-MS/MS
CID 30.0
880.39
620.36
Precursor (m/z): 1,374.82
Isolation width (m/z): 2
1,164.62
1,146.61
638.36
800.42

I

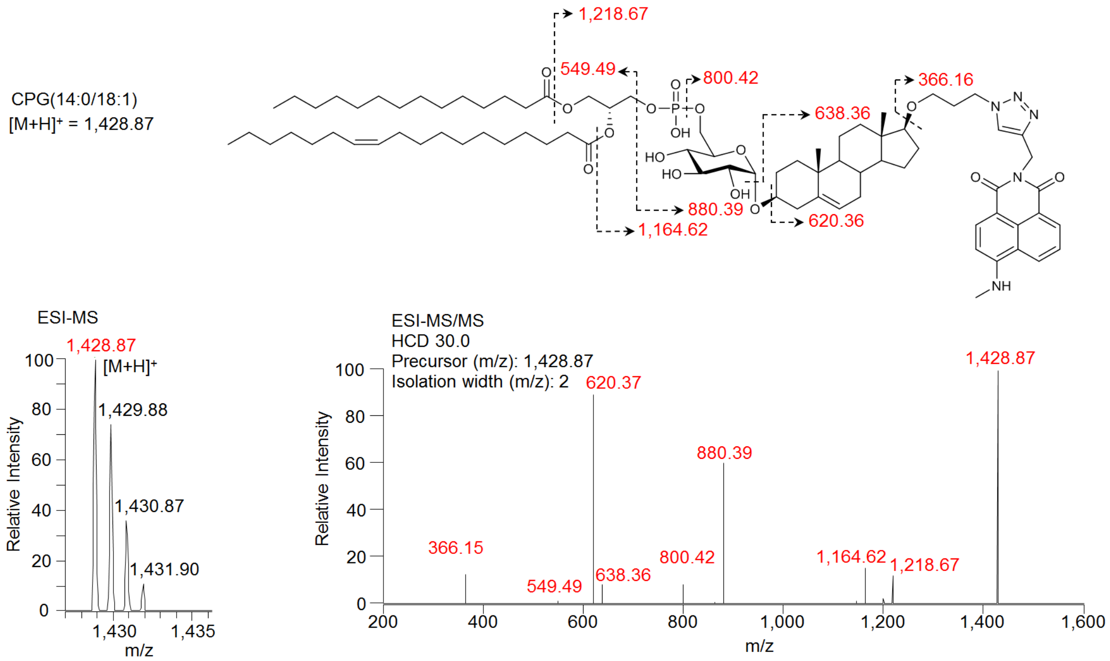

CPG(14:0/18:1)
[M+H]+ = 1,428.87
1,218.67
549.49
800.42
366.16
638.36
880.39
620.36
1,164.62
ESI-MS
1,428.87
[M+H]+
1,429.88
1,430.87
1,431.90
Relative Intensity
m/z
ESI-MS/MS
HCD 30.0
Precursor (m/z): 1,428.87
Isolation width (m/z): 2
620.37
1,428.87
880.39
366.15
549.49
638.36
800.42
1,164.62
1,218.67

J

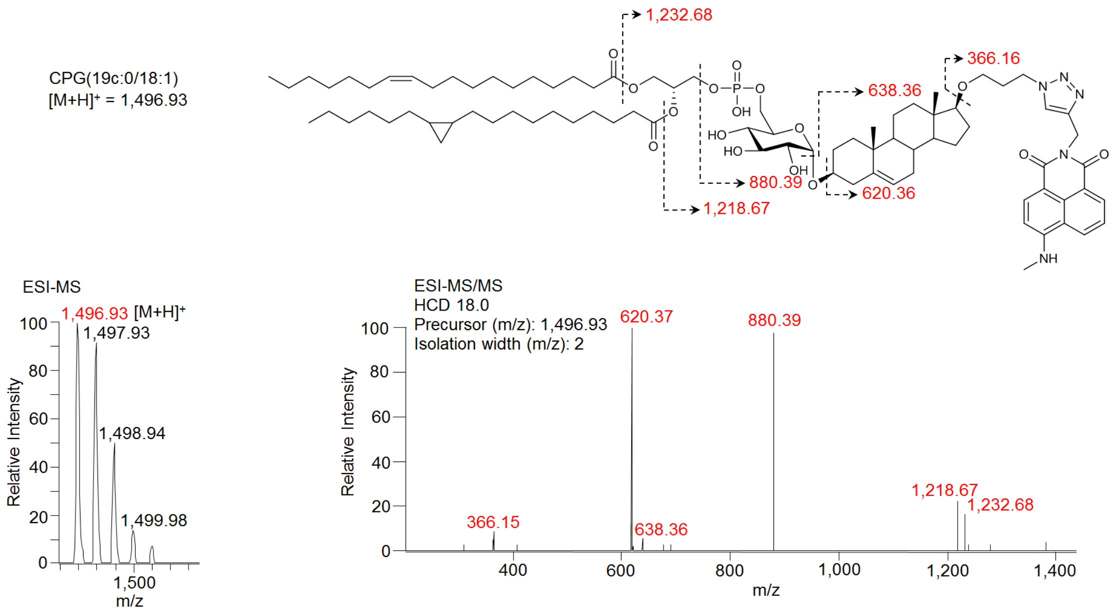
CPG(19c:0/18:1)
[M+H]+ = 1,496.93
1,232.68
366.16
638.36
880.39
620.36
1,218.67
ESI-MS
1,496.93 [M+H]+
1,497.93
1,498.94
1,499.98
Relative Intensity
m/z
ESI-MS/MS
HCD 18.0
Precursor (m/z): 1,496.93
Isolation width (m/z): 2
620.37
880.39
366.15
638.36
1,218.67
1,232.68

K

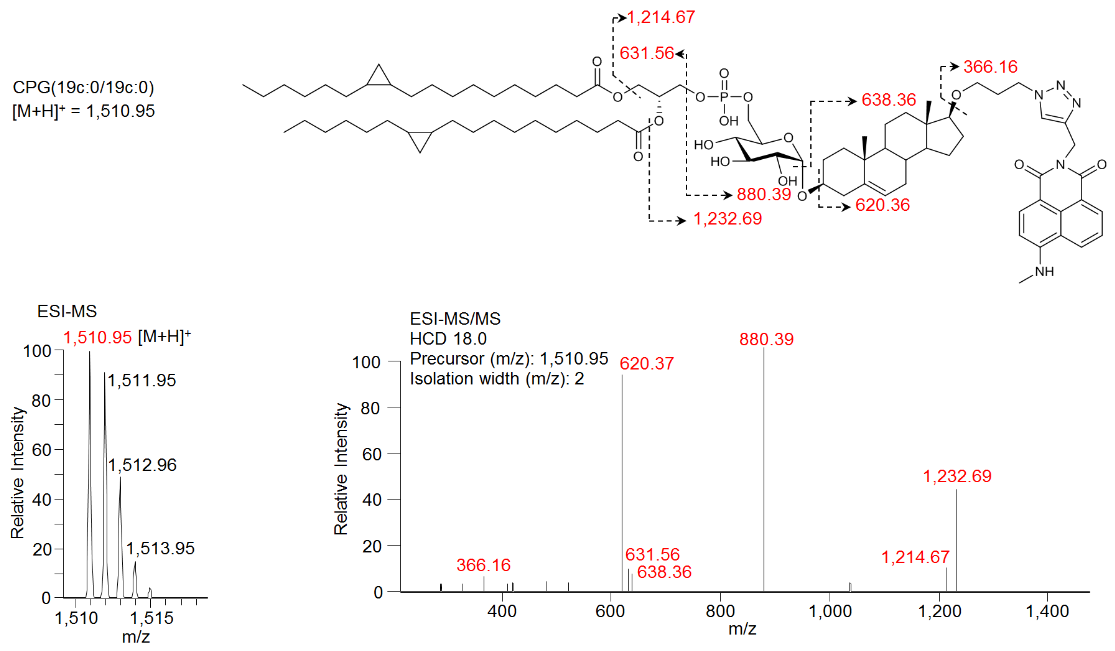
CPG(19c:0/19c:0)
[M+H]+ = 1,510.95
1,214.67
631.56
366.16
638.36
880.39
620.36
1,232.69
ESI-MS
1,510.95 [M+H]+
1,511.95
1,512.96
1,513.95
Relative Intensity
m/z
ESI-MS/MS
HCD 18.0
Precursor (m/z): 1,510.95
Isolation width (m/z): 2
620.37
880.39
1,232.69
366.16
631.56
638.36
1,214.67

L

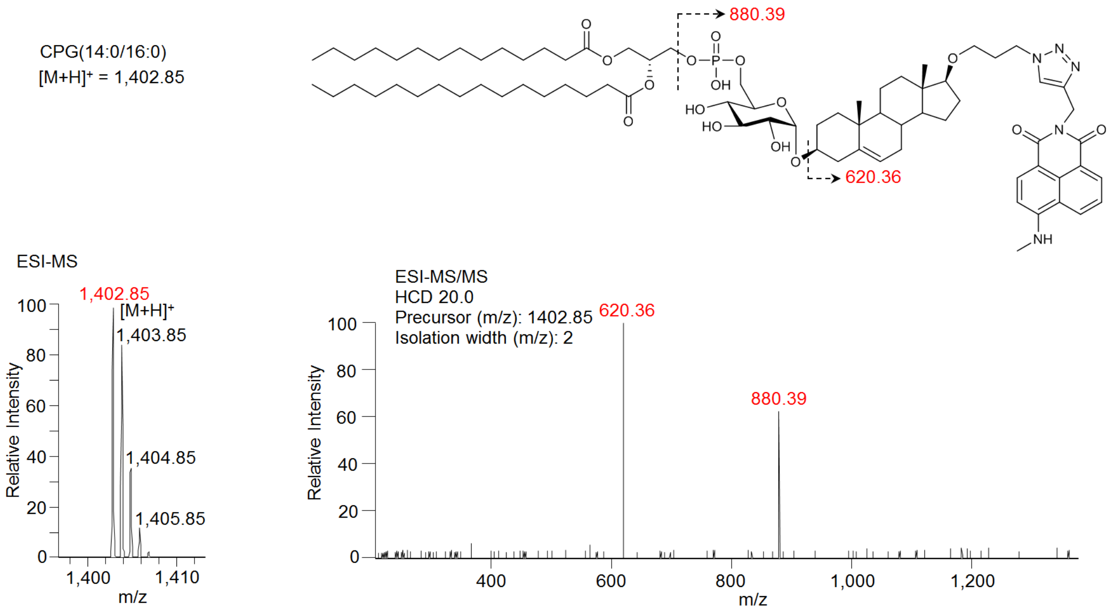
CPG(14:0/16:0)
[M+H]+ = 1,402.85
880.39
620.36
ESI-MS
1,402.85
[M+H]+
1,403.85
1,404.85
1,405.85
Relative Intensity
m/z
ESI-MS/MS
HCD 20.0
Precursor (m/z): 1402.85
Isolation width (m/z): 2
620.36
880.39
Relative Intensity
m/z

M

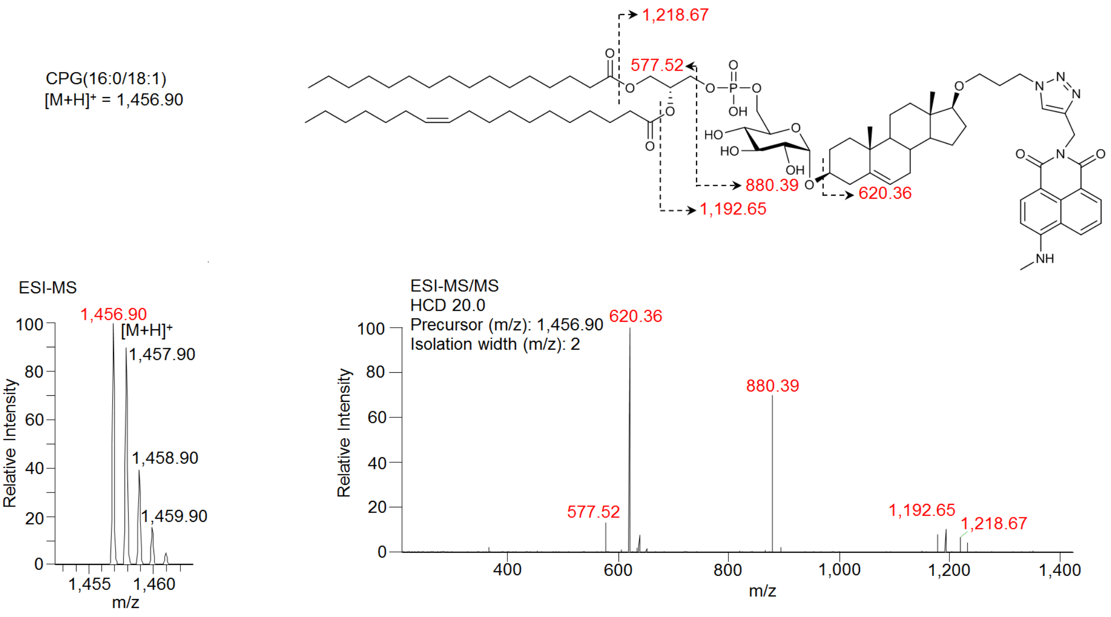
CPG(16:0/18:1)
[M+H]+ = 1,456.90
1,218.67
577.52
880.39
620.36
1,192.65
ESI-MS
1,456.90
[M+H]+
1,457.90
1,458.90
1,459.90
Relative Intensity
m/z
ESI-MS/MS
HCD 20.0
Precursor (m/z): 1,456.90
Isolation width (m/z): 2
620.36
880.39
577.52
1,192.65
1,218.67
Relative Intensity
m/z

N

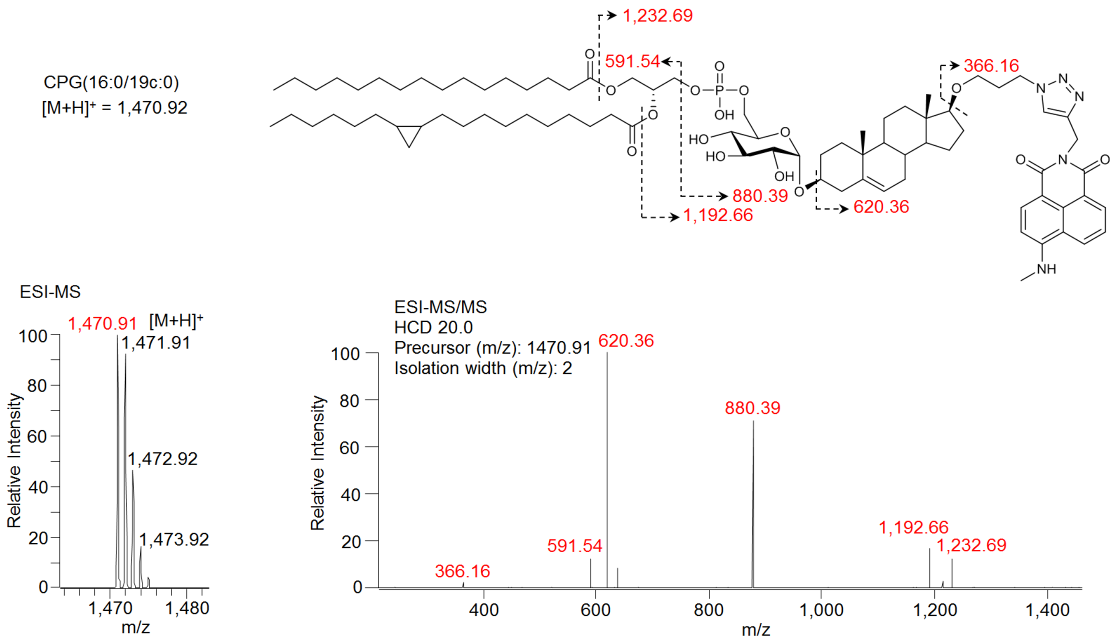

CPG(16:0/19c:0)
[M+H]+ = 1,470.92
1,232.69
591.54
366.16
880.39
620.36
1,192.66
ESI-MS
1,470.91 [M+H]+
1,471.91
1,472.92
1,473.92
Relative Intensity
m/z
ESI-MS/MS
HCD 20.0
Precursor (m/z): 1470.91
Isolation width (m/z): 2

O

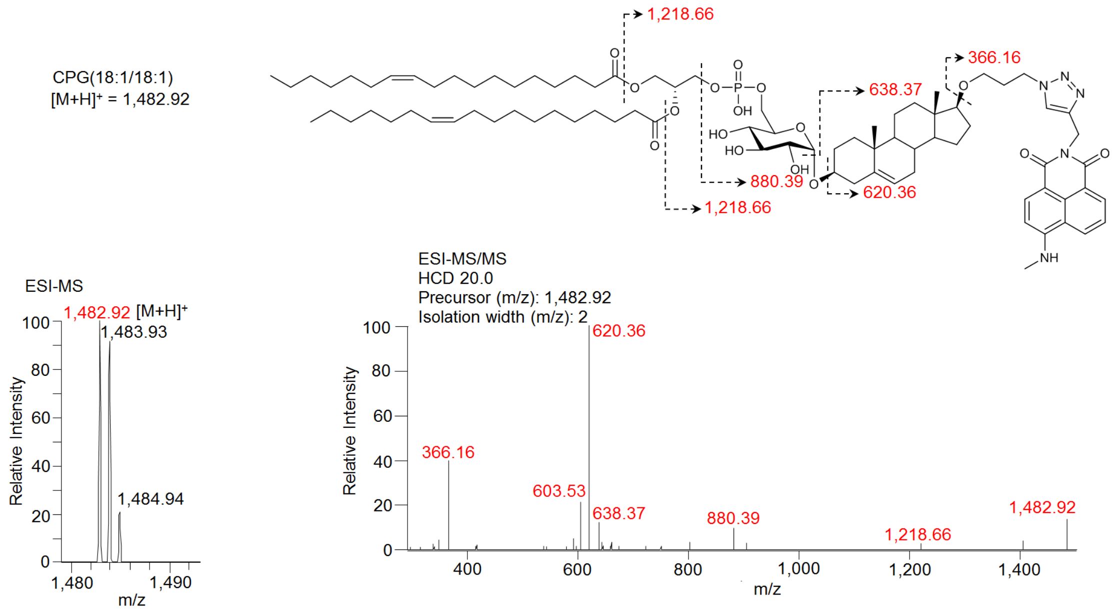

CPG(18:1/18:1)
[M+H]+ = 1,482.92
1,218.66
366.16
638.37
880.39
620.36
1,218.66
ESI-MS
1,482.92 [M+H]+
1,483.93
1,484.94
Relative Intensity
m/z
ESI-MS/MS
HCD 20.0
Precursor (m/z): 1,482.92
Isolation width (m/z): 2
620.36
366.16
603.53
638.37
880.39
1,482.92
1,218.66

P

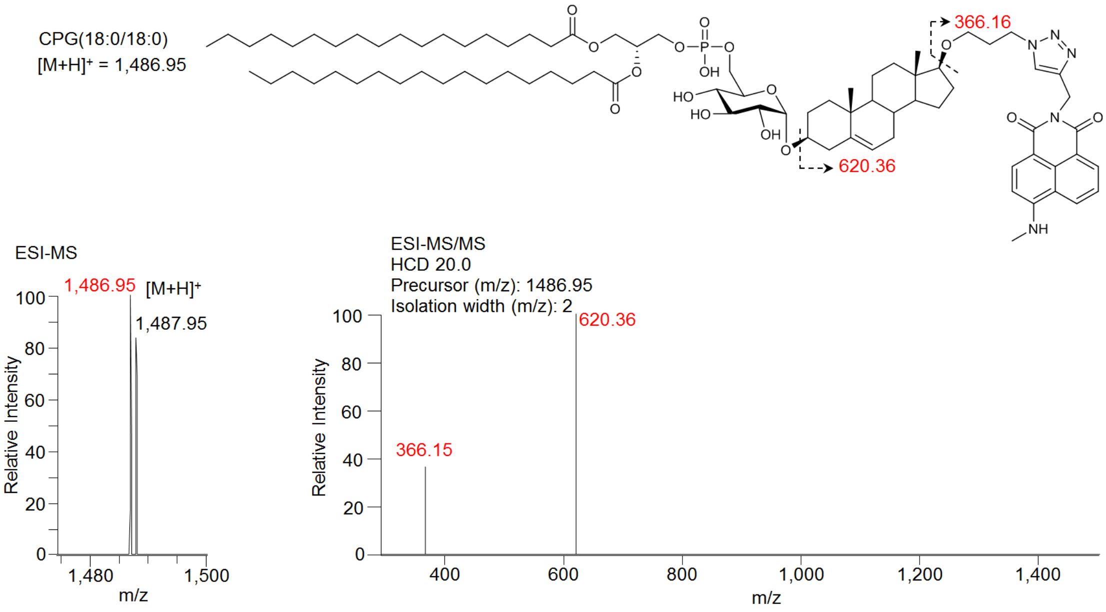
CPG(18:0/18:0)
[M+H]+ = 1,486.95
366.16
620.36
ESI-MS
1,486.95 [M+H]+
1,487.95
Relative Intensity
m/z
ESI-MS/MS
HCD 20.0
Precursor (m/z): 1486.95
Isolation width (m/z): 2
620.36
366.15
Relative Intensity
m/z

Q

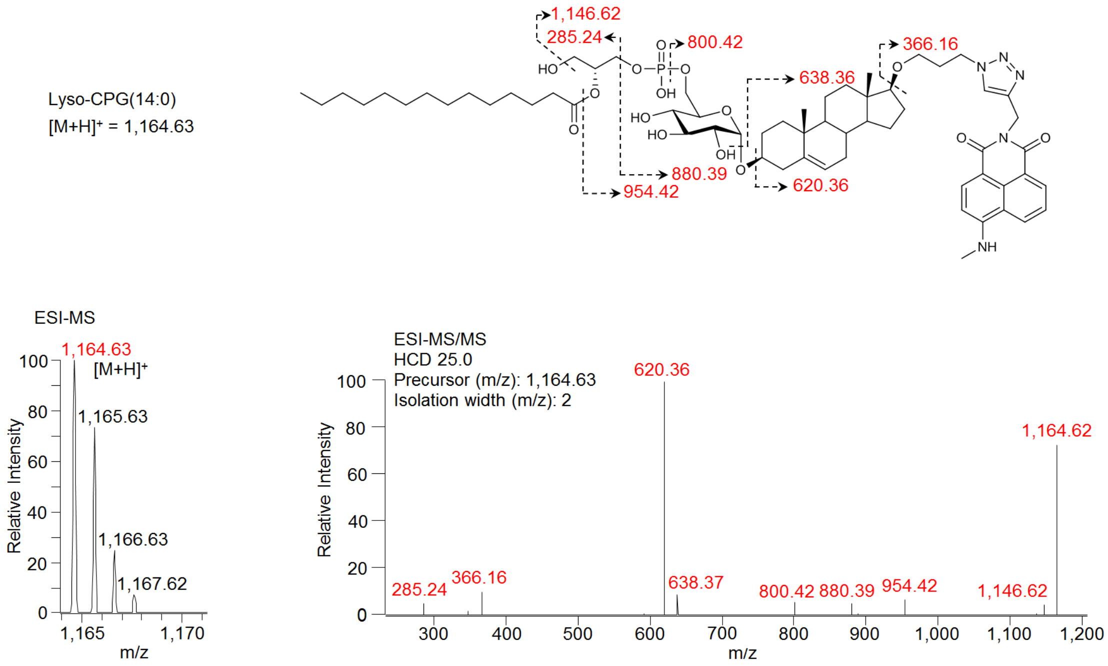
Lyso-CPG(14:0)
[M+H]+ = 1,164.63
1,146.62
285.24
800.42
638.36
366.16
880.39
954.42
620.36
ESI-MS
1,164.63 [M+H]+
1,165.63
1,166.63
1,167.62
Relative Intensity
m/z
ESI-MS/MS
HCD 25.0
Precursor (m/z): 1,164.63
Isolation width (m/z): 2
620.36
1,164.62
285.24
366.16
638.37
800.42
880.39
954.42
1,146.62
Relative Intensity
m/z

R

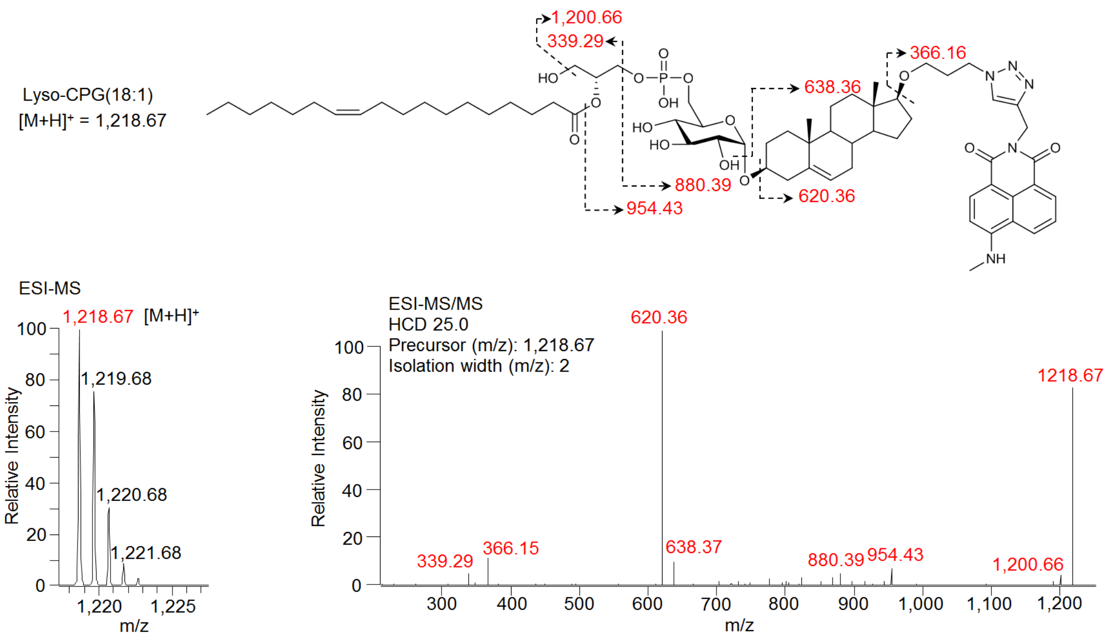
Lyso-CPG(18:1)
[M+H]+ = 1,218.67
1,200.66
339.29
366.16
638.36
880.39
954.43
620.36
ESI-MS
1,218.67 [M+H]+
1,219.68
1,220.68
1,221.68
Relative Intensity
m/z
ESI-MS/MS
HCD 25.0
Precursor (m/z): 1,218.67
Isolation width (m/z): 2
620.36
1218.67
339.29
366.15
638.37
880.39
954.43
1,200.66

S

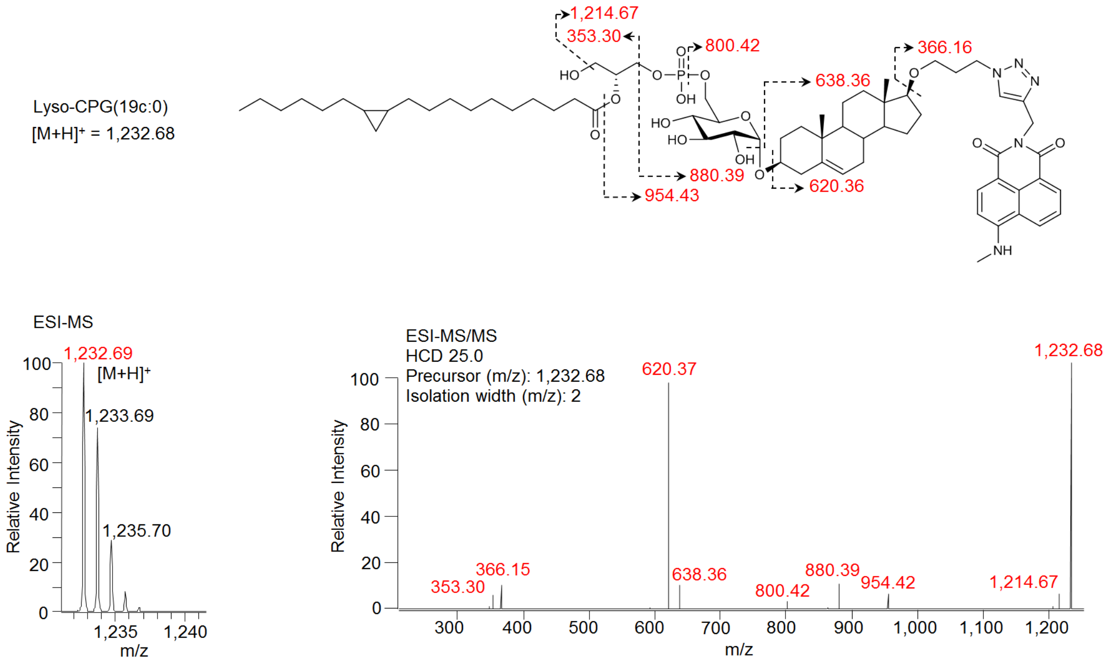
Lyso-CPG(19c:0)
[M+H]+ = 1,232.68
1,214.67
353.30
800.42
366.16
638.36
880.39
954.43
620.36
ESI-MS
1,232.69
[M+H]+
1,233.69
1,235.70
Relative Intensity
m/z
ESI-MS/MS
HCD 25.0
Precursor (m/z): 1,232.68
Isolation width (m/z): 2
620.37
1,232.68
366.15
353.30
638.36
880.39
800.42
954.42
1,214.67

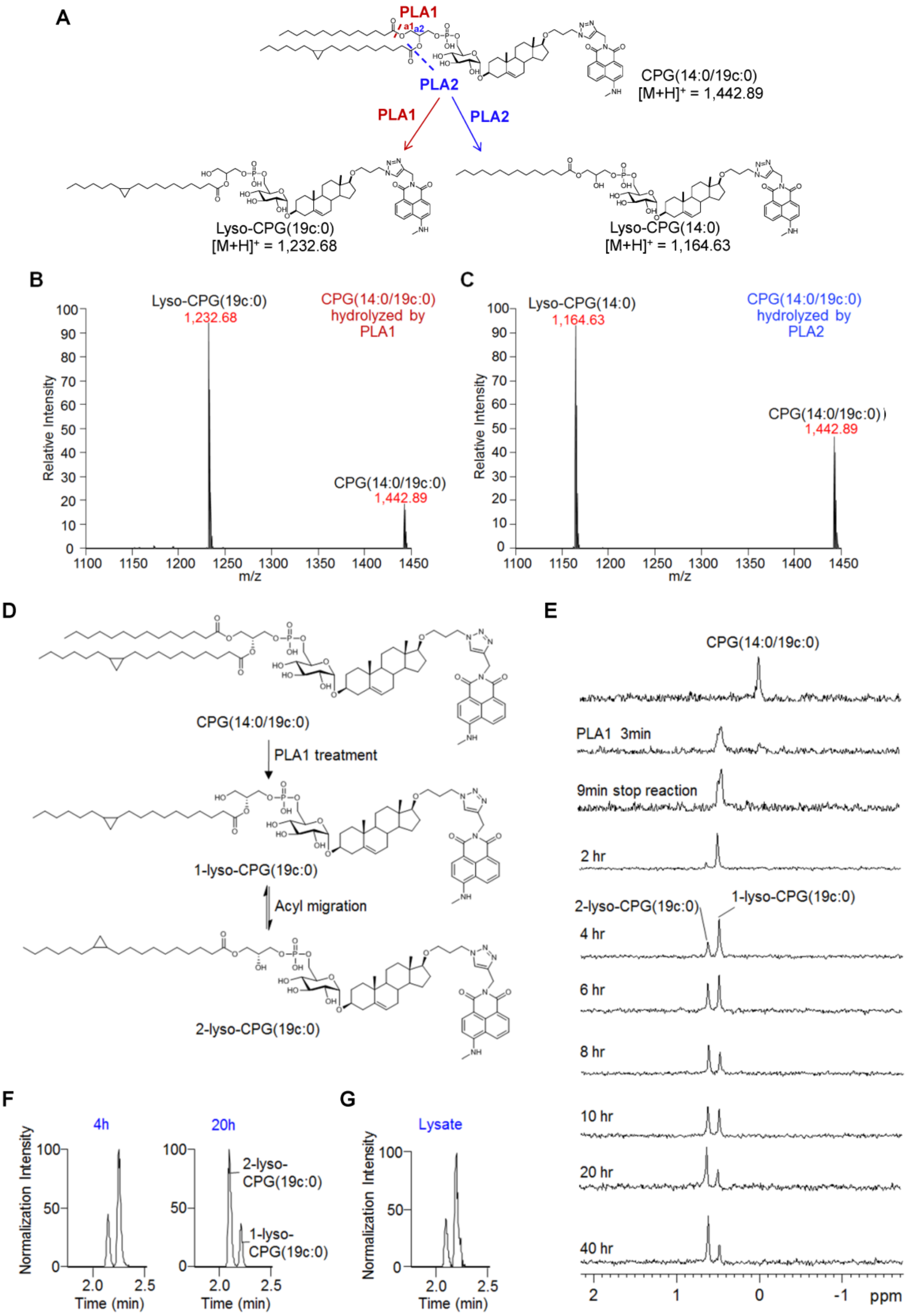

Fig. S4. Structural determination of CPG(14:0/19c:0) by using enzymatic hydrolysis (A) A scheme presents the regioselective hydrolysis of CPG(14:0/19c:0) by phospholipase A1 (PLA1, left) or phospholipase A2 (PLA2, right). (B) and (C) show the UPLC-MS spectra of the products of PLA1 and PLA2 reactions, respectively. (D-G) ^{31}P NMR employed to monitor the phospholipase A1 (PLA1)-catalyzed hydrolysis of CPG(14:0/19c:0) (D) The scheme shows PLA1-catalyzed hydrolysis of

CPG(14:0/19c:0) to give 1-lyso-CPG(19c:0), followed by acyl migration to additionally produce 2-lyso-CPG(19c:0). (E) ^{31}P NMR spectra were used to monitor the formation of 1-lyso-CPG(19c:0) (the chemical shift is ~δ 0.5) and 2-lyso-CPG(19c:0) (~δ 0.6). (F) The signals in the ^{31}P NMR spectra were further confirmed by UPLC-MS analysis. For instance, the previous samples at 4 h and 20 h were analyzed by UPLC-MS to indicate the consistent signal ratios. (G) The mixture of lyso-CPG(19c:0) obtained from the lysate was analyzed by UPLC-MS.

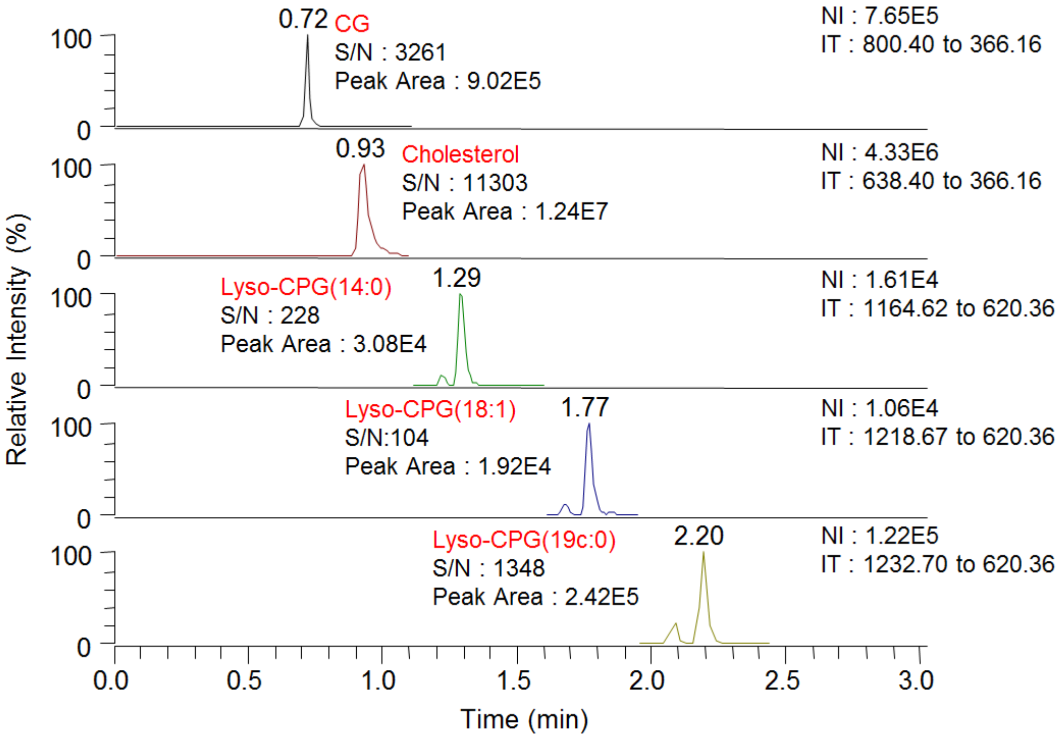

0.72
CG
S/N : 3261
Peak Area : 9.02E5
NI : 7.65E5
IT : 800.40 to 366.16
0.93
Cholesterol
S/N : 11303
Peak Area : 1.24E7
NI : 4.33E6
IT : 638.40 to 366.16
Lyso-CPG(14:0)
S/N : 228
Peak Area : 3.08E4
1.29
NI : 1.61E4
IT : 1164.62 to 620.36
Lyso-CPG(18:1)
S/N:104
Peak Area : 1.92E4
1.77
NI : 1.06E4
IT : 1218.67 to 620.36
Lyso-CPG(19c:0)
S/N : 1348
Peak Area : 2.42E5
2.20
NI : 1.22E5
IT : 1232.70 to 620.36
Relative Intensity (%)
Time (min)

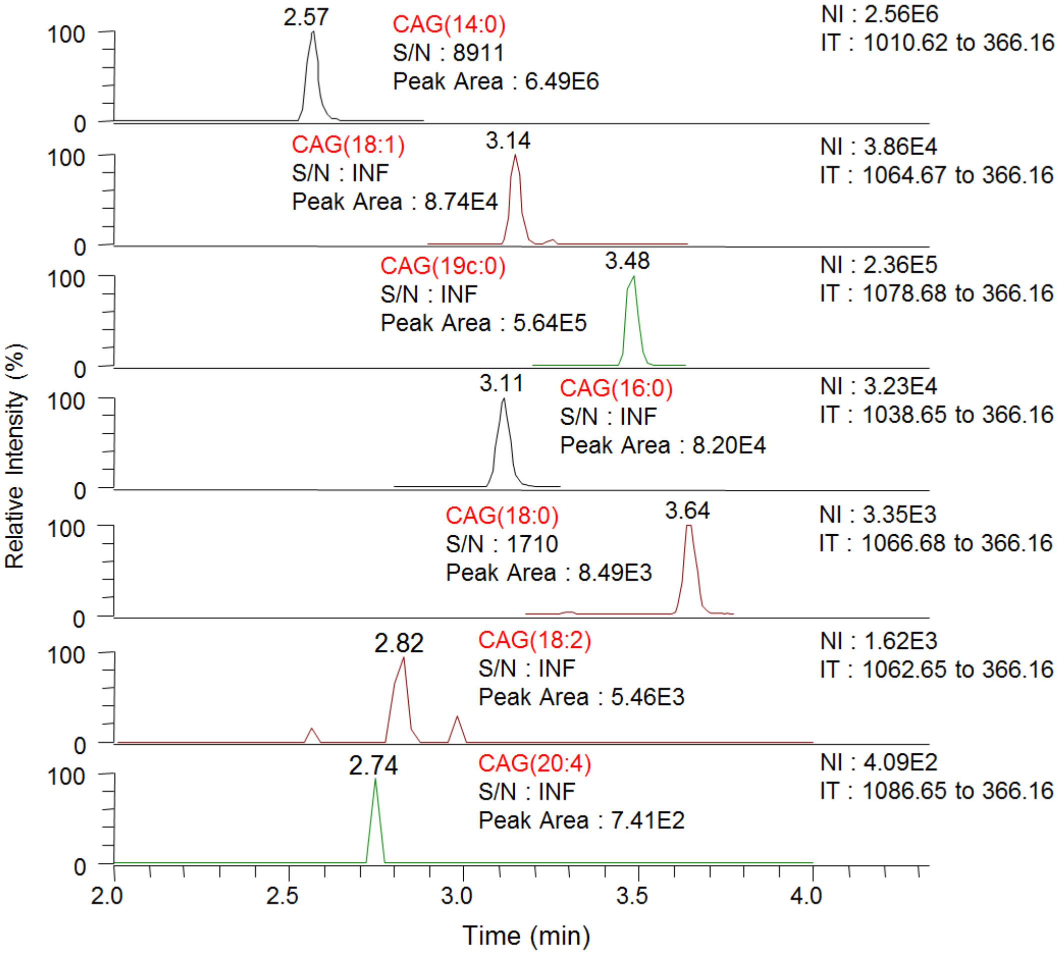

2.57
CAG(14:0)
S/N : 8911
Peak Area : 6.49E6
NI : 2.56E6
IT : 1010.62 to 366.16
CAG(18:1)
S/N : INF
Peak Area : 8.74E4
3.14
NI : 3.86E4
IT : 1064.67 to 366.16
CAG(19c:0)
S/N : INF
Peak Area : 5.64E5
3.48
NI : 2.36E5
IT : 1078.68 to 366.16
3.11
CAG(16:0)
S/N : INF
Peak Area : 8.20E4
NI : 3.23E4
IT : 1038.65 to 366.16
CAG(18:0)
S/N : 1710
Peak Area : 8.49E3
3.64
NI : 3.35E3
IT : 1066.68 to 366.16
2.82
CAG(18:2)
S/N : INF
Peak Area : 5.46E3
NI : 1.62E3
IT : 1062.65 to 366.16
2.74
CAG(20:4)
S/N : INF
Peak Area : 7.41E2
NI : 4.09E2
IT : 1086.65 to 366.16
Relative Intensity (%)
Time (min)

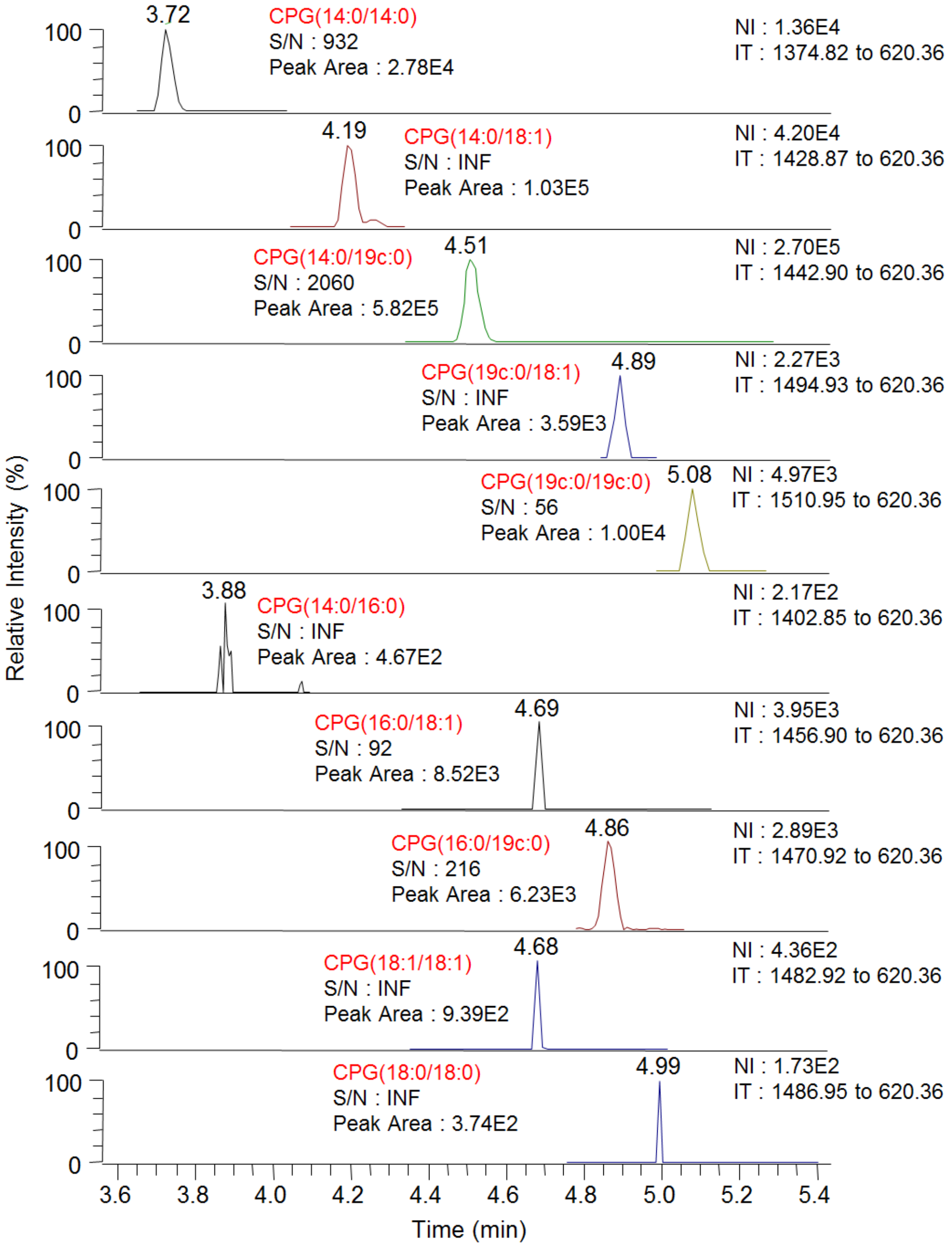

Fig. S5. Selected ion chromatograms for specific transitions of different fluorophore-labeled CGds MAN-labeled CGds were analyzed by UPLC-MS/MS in product ion scan mode. IT, ion transition, denotes the specific fragmentation peaks (m/z of 366.16 or 620.36) that are disintegrated from various target parent ions. S/N denotes signal-to-noise ratio. INF denotes infinity. NI denotes normalization intensity. Peak area was obtained from the specific fragmentation peak and used to quantify MAN-labeled CGds by the calibration curves.

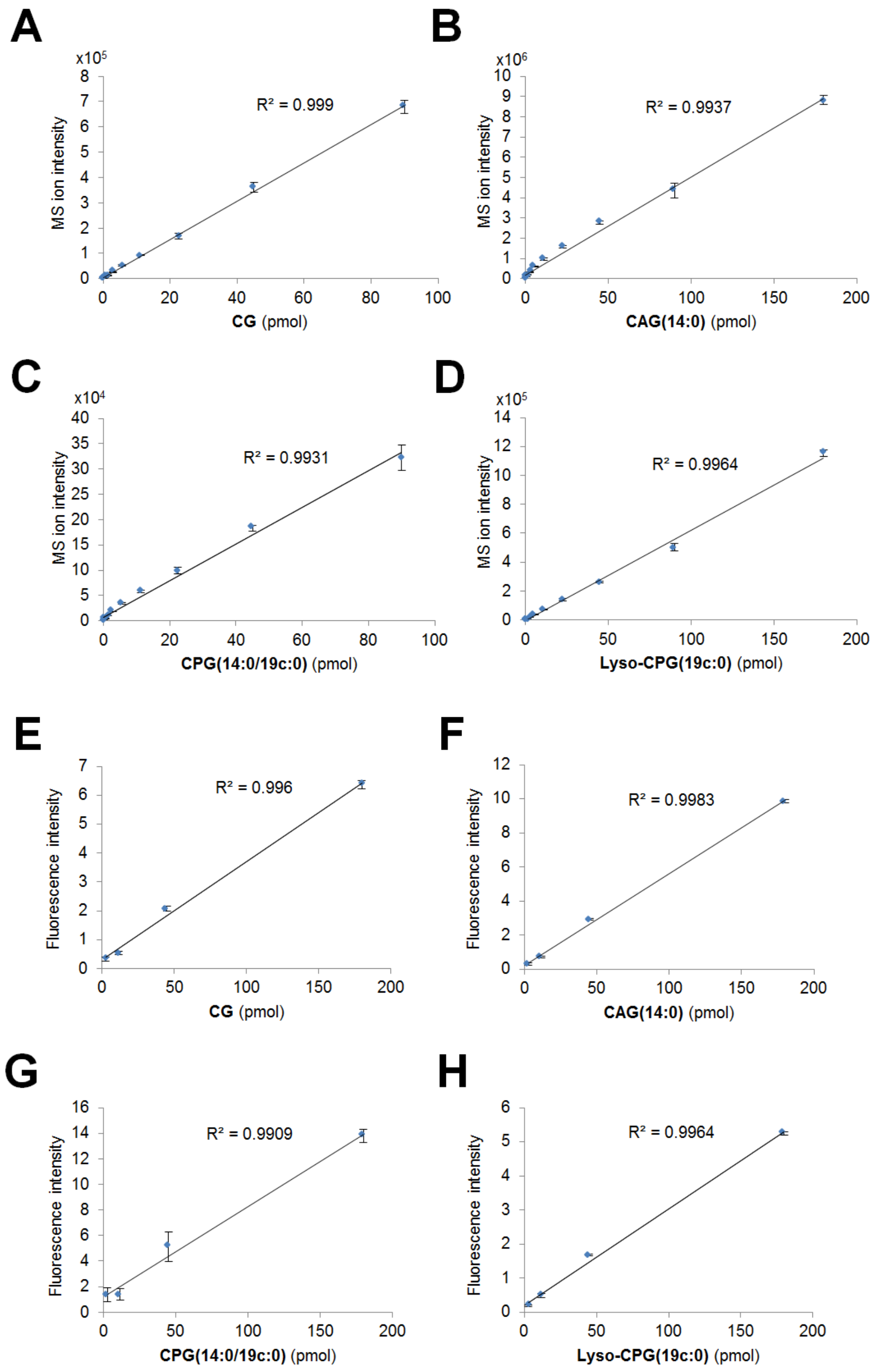

Fig. S6. Calibration curves of MAN-labeled CG, CAG, CPG and lyso-CPG analyzed by UPLC-MS/MS and HPLC-fluorescence. (A-D) Calibration curves of MAN-labeled CGds analyzed by UPLC-MS/MS. Calibration curves were obtained by analyzing the MS ion intensities of standards at 0.088, 0.176, 0.35, 0.7, 1.4, 2.8, 5.6, 11.25, 22.5, 45 and 90 pmol measured by UPLC-MS operated in product ion scan mode (UPLC-MS/MS). MAN-labeled CPG(14:0/19c:0), CAG(14:0), Lyso-CPG(19c:0) and CG were purified from bacteria lysate and used as the standard compounds for establishing the calibration curves. (E-H) Calibration curves of MAN-labeled CGds analyzed by

HPLC-fluorescence. HPLC-fluorescence means HPLC analysis by using fluorescence detection for quantification (λ_{exi} and λ_{emi}= 428 and 528 nm, respectively). The calibration curves were obtained by measuring fluorescence intensities of purified CG, CAG(14:0), CPG(14:0/19c:0) and lyso-CPG(19c:0) in a series of dilutions. Data shown were from one experiment with three technical replicates. Error bars represented standard deviations.

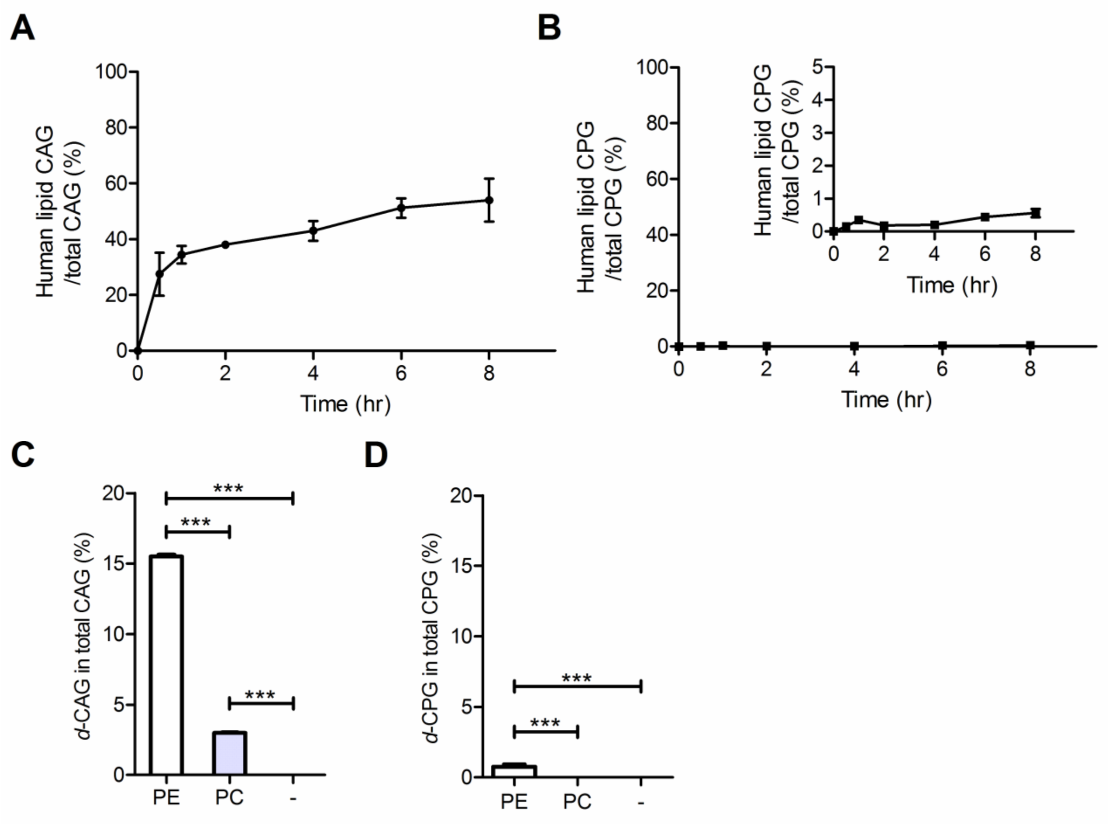

Fig. S7. Levels of the incorporated CAGs and CPGs. (A and B) Levels of the incorporated CAGs and CPGs at different times. Previously in the main text, *H. pylori* was shown to take human lipids for the biosynthesis of CAGs and CPGs in *H. pylori*. (A) Ratios of human-lipid-containing CAGs to total CAGs in *H. pylori* (26695) at 0.5, 1, 2, 4, 6, 8 h after co-culturing with AGS cells. *H. pylori* was used to infect AGS cells and the amount of CAGs containing different acyl chains was measured by UPLC-MS. Human-lipid-containing CAGs are composed of CAG(16:0), CAG(18:0), CAG(18:1), CAG(18:2) and CAG(20:4). In addition to human-lipid-containing CAGs, total CAGs also contain CAG(14:0) and CAG(19c:0) that are dominant in *H. pylori*. (B) Ratios of human-lipid-containing CPGs to total CPGs in *H. pylori* (26695) at 0.5, 1, 2, 4, 6, 8 h after co-culturing with AGS cells. Human-lipid-containing CPGs are composed of CPG(16:0/18:1), CPG(18:0/18:0) and CPG(18:1/18:1). In addition to human-lipid-containing CPGs, total CPGs also contain CPG(14:0/14:0), CPG(14:0/18:1) and CPG(14:0/19c:0) that are dominant in *H. pylori*. 5% of the y axis of (B) was expanded and shown in the inserted. (C and D) Relative levels of deuterium-labeled CAG (C) and CPG (D) in the *H. pylori*-infected AGS cells. AGS cells were first treated with deuterium-labeled phosphatidylethanolamine (PE) or phosphatidylcholine (PC) for 1 h, and then infected with *H. pylori* (26695) for 6 h. The ratios of deuterium-labeled CAG and CPG (namely *d*-CAG and *d*-CPG, respectively) to total CAGs and CPGs were determined by the area of product ion signals shown in the UPLC-MS spectra. Data shown were from three biological replicates. Error bars represented standard deviations. All statistically significant differences are indicated with asterisks, ***P < 0.001, based on Student's *t* test.

Supplementary tables

Table S1 Kinetic parameters of cholesterol and its analogues in the catalysis of cholesterol-α-glucosyltransferase[a]

substrate	k_{cat} (min^{-1})	K_m (μM)	k_{cat}/K_m ($\mu M^{-1}min^{-1}$)	Normalized catalytic efficiency (%)
Cholesterol	3.58 ± 0.37	66.33 ± 15.99	0.0539 ± 0.0141	100.0
**1**	6.13 ± 0.46	134.80 ± 19.65	0.0455 ± 0.0074	84.3
**2**	6.82 ± 0.41	193.10 ± 20.25	0.0353 ± 0.0043	65.5
**3**	10.41 ± 2.65	324.70 ± 88.90	0.0321 ± 0.0105	59.5
Ergosterol	0.30 ± 0.11	40.70 ± 17.20	0.0070 ± 0.0033	12.9
Stigmasterol	0.68 ± 0.14	186.30 ± 62.25	0.0036 ± 0.0014	6.73

[a] The measurement was carried out at 37 °C. On the saturated concentration of UDP-glucose (2mM), steady state velocities were fitted to the equation: v = V_{max} × [X]/(K_m + X), [X] represent the concentrations of cholesterol. K_m denotes the Michaelis constants of cholesterol. k_{cat} is the turnover number. The catalytic efficiency can be expressed by the term k_{cat}/K_m. Values were the means of three determinations ± standard deviations. Notably the velocities of 22-NBD-cholesterol and 25-NBD-cholesterol were not able to fit to the equation due to little or no consumption of NADH in their reaction. 22-NBD-cholesterol represents 22-(*N*-(7-nitrobenz-2-oxa-1,3-diazol-4-yl)amino) -23,24-bisnor-5 -cholen-3*β*-ol; 25-NBD- cholesterol denotes 25-[*N*-[(7-nitrobenz-2-oxa-1,3-diazol-4-yl) -methyl]amino]-27-norcholesterol.[3, 4, 9]

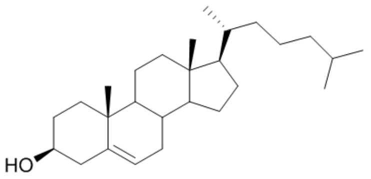

Cholesterol

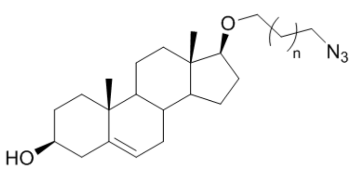

1 (n = 1), **2** (n = 4), and **3** (n = 7)

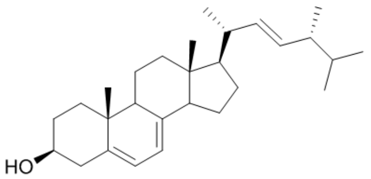

Ergosterol

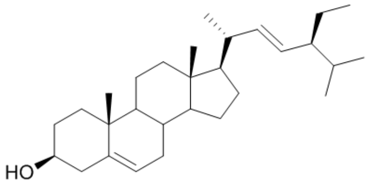

Stigmasterol

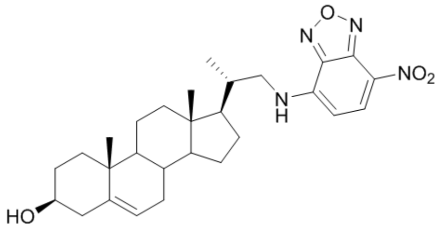

22-NBD-cholesterol

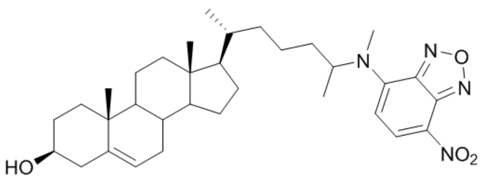

25-NBD-cholesterol

Table S2. The theoretical and observed m/z (mass-to-charge ratio) and RIA (relative isotopic abundance) errors of the molecular ions of CGds detected in UPLC-MS analysis

MAN-labeled CG compounds	Ion formula	Theor. m/z $[M+H]^+$	Observed m/z $[M+H]^+$	MS1 Δppm	RIA error (%)
CAG(14:0)	$C_{58}H_{84}N_5O_{10}$	1010.621	1010.619	2.25	4.36
CAG(18:1)	$C_{62}H_{90}N_5O_{10}$	1064.668	1064.665	3.02	4.43
CAG(19c:0)	$C_{63}H_{92}N_5O_{10}$	1078.684	1078.683	0.81	4.15
CAG(16:0)	$C_{60}H_{88}N_5O_{10}$	1038.653	1038.657	3.97	3.19
CAG(18:0)	$C_{62}H_{92}N_5O_{10}$	1066.684	1066.684	0.49	2.15
CAG(18:2)	$C_{62}H_{88}N_5O_{10}$	1062.653	1062.657	4.07	1.77
CAG(20:4)	$C_{64}H_{88}N_5O_{10}$	1086.653	1086.654	1.21	4.84
CPG(14:0/14:0)	$C_{75}H_{117}N_5O_{16}P$	1374.823	1374.818	3.45	4.37
CPG(14:0/18:1)	$C_{79}H_{123}N_5O_{16}P$	1428.870	1428.870	0.21	3.85
CPG(14:0/19c:0)	$C_{80}H_{125}N_5O_{16}P$	1442.885	1442.890	3.22	4.64
CPG(19c:0/18:1)	$C_{84}H_{131}N_5O_{16}P$	1496.932	1496.930	1.29	2.28
CPG(19c:0/19c:0)	$C_{85}H_{133}N_5O_{16}P$	1510.948	1510.946	1.54	3.21
CPG(14:0/16:0)	$C_{77}H_{121}N_5O_{16}P$	1402.854	1402.849	3.62	1.11
CPG(16:0/18:1)	$C_{81}H_{127}N_5O_{16}P$	1456.901	1456.900	0.88	1.08
CPG(16:0/19c:0)	$C_{82}H_{129}N_5O_{16}P$	1470.917	1470.911	3.59	2.20
CPG(18:1/18:1)	$C_{83}H_{129}N_5O_{16}P$	1482.917	1482.924	4.75	3.00
CPG(18:0/18:0)	$C_{83}H_{133}N_5O_{16}P$	1486.948	1486.951	2.15	2.15
Lyso-CPG(14:0)	$C_{61}H_{91}N_5O_{15}P$	1164.624	1164.626	1.39	3.44
Lyso-CPG(18:1)	$C_{65}H_{97}N_5O_{15}P$	1218.671	1218.668	2.90	3.76
Lyso-CPG(19c:0)	$C_{66}H_{99}N_5O_{15}P$	1232.687	1232.685	1.61	3.01

Table S3. Parameters for the targeted measurement of CGds using UPLC-MS/MS[a]

MAN-labeled compounds	Parent m/z [b]	Daughter m/z	Type of ion dissociation	CE (%)
CG	800.40	366.16	CID	40
Cholesterol	638.40	366.16	CID	30
Lyso-CPG(14:0)	1164.62	620.36	HCD	30
Lyso-CPG(18:1)	1218.67	620.36	HCD	30
Lyso-CPG(19c:0)	1232.70	620.36	HCD	30
CAG(14:0)	1010.62	366.16	HCD	30
CAG(18:1)	1064.67	366.16	HCD	30
CAG(19c:0)	1078.68	366.16	HCD	30
CAG(16:0)	1038.65	366.16	HCD	30
CAG(18:0)	1066.68	366.16	HCD	30
CAG(18:2)	1062.65	366.16	HCD	30
CAG(20:4)	1086.65	366.16	HCD	30
CPG(14:0/14:0)	1374.82	620.36	HCD	30
CPG(14:0/18:1)	1428.87	620.36	HCD	30
CPG(14:0/19c:0)	1442.90	620.36	HCD	30
CPG(19c:0/18:1)	1494.93	620.36	HCD	30
CPG(19c:0/19c:0)	1510.95	620.36	HCD	30
CPG(14:0/16:0)	1402.85	620.36	HCD	30
CPG(16:0/18:1)	1456.90	620.36	HCD	30
CPG(16:0/19c:0)	1470.92	620.36	HCD	30
CPG(18:1/18:1)	1482.92	620.36	HCD	30
CPG(18:0/18:0)	1486.95	620.36	HCD	30

[a] This table provides a summary of the MS transitions and collision mode/energy of MAN-labeled CGds analyzed by UPLC-MS/MS. Figure S5 shows the detailed data. CE: collision energy, CID: collision-induced dissociation, HCD: higher-energy collisional dissociation, [b]: Isolation width for parent Ion was set to 2.0 Th

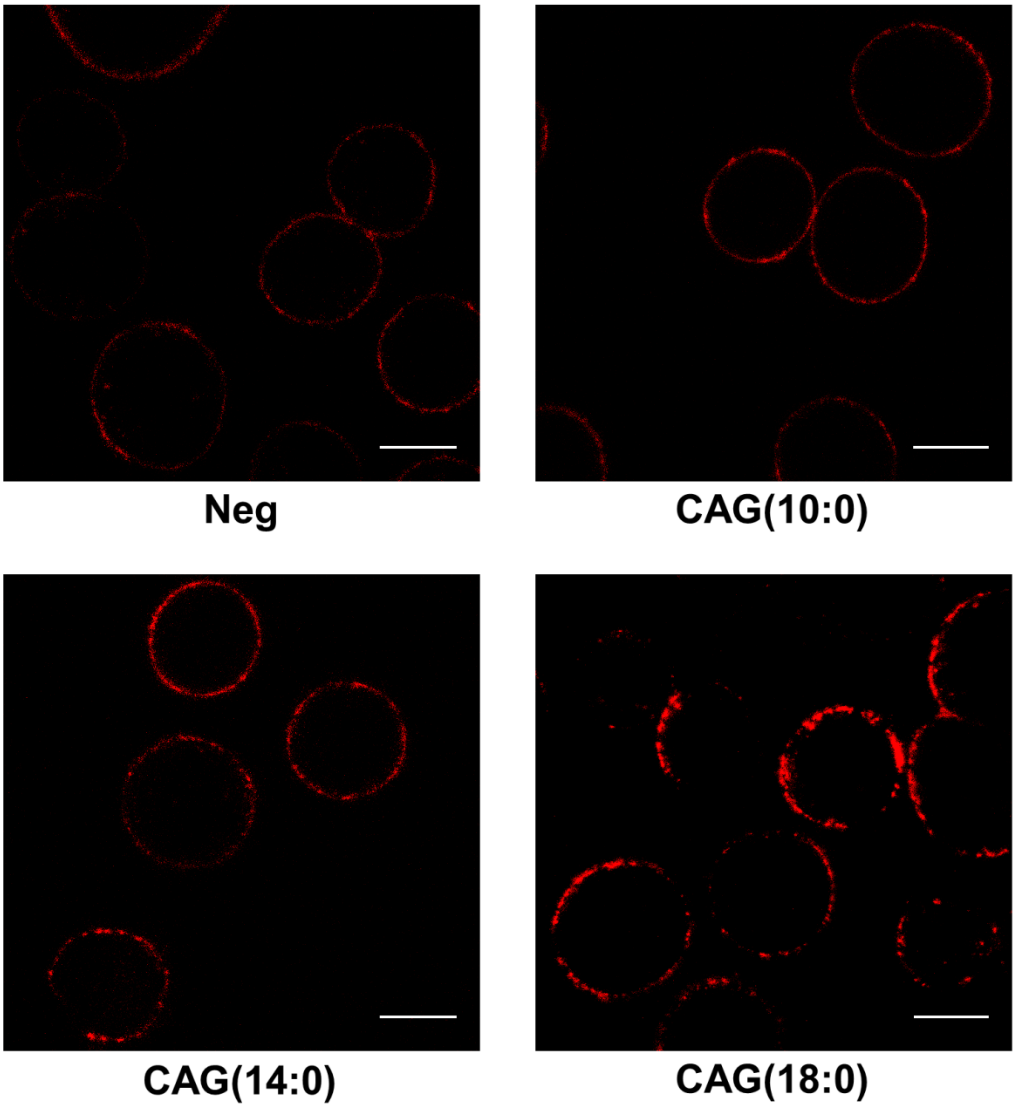

Supplementary movies. Movies of AGS cells that were treated with CAGs (**(10:0)**, **(14:0)** or **(18:0)**). Lipid rafts (GM1) in AGS cells were first labelled with Alexa Fluor 594-conjugated cholera toxin subunit β, followed by the treatment with indicated CAGs (10:0, 14:0 or 18:0) at 37 °C. Time-lapse images were collected under a Zeiss LSM 510 confocal microscope at 0, 5, 10, 15, 20, 25, 30 and 35 min. The figures shown in here represented the images that collected at 35 min. Scale bars, 10 μm.

1. H. Q. Nguyen, R. A. Davis and J. Gervay-Hague, *Angew. Chem., Int. Ed.*, 2014, **53**, 13400-13403.
2. H. J. Wang, W. C. Cheng, H. H. Cheng, C. H. Lai and W. C. Wang, *Mol. Microbiol.*, 2012, **83**, 67-84.
3. P. Gonzalo, B. Sontag, D. Guillot and J. P. Reboud, *Anal. Biochem.*, 1995, **225**, 178-180.
4. H. Lee, P. Wang, H. Hoshino, Y. Ito, M. Kobayashi, J. Nakayama, P. H. Seeberger and M. Fukuda, *Glycobiology*, 2008, **18**, 549-558.
5. X. Zhou and G. Arthur, *J. Lipid Res.*, 1992, **33**, 1233-1236.
6. H. Kuge, K. Akahori, K. Yagyu and K. Honke, *J. Biol. Chem.*, 2014, **289**, 26783-26793.
7. M. K. Mishra, T. Kumaraguru, G. Sheelu and N. W. Fadnavis, *Tetrahedron-Asymmetry*, 2009, **20**, 2854-2860.
8. J. Lessig, J. Schiller, J. Arnhold and B. Fuchs, *J. Lipid Res.*, 2007, **48**, 1316-1324.
9. A. H. Lebrun, C. Wunder, J. Hildebrand, Y. Churin, U. Zahringer, B. Lindner, T. F. Meyer, E. Heinz and D. Warnecke, *J. Biol. Chem.*, 2006, **281**, 27765-27772.
